# Primary amines from lignocellulose by direct amination of alcohol intermediates, catalyzed by RANEY® Ni†

**DOI:** 10.1039/d2cy00864e

**Published:** 2022-08-19

**Authors:** Xianyuan Wu, Mario De Bruyn, Katalin Barta

**Affiliations:** Stratingh Institute for Chemistry, University of Groningen Nijenborgh 4 9747 AG The Netherlands; Institute for Chemistry, University of Graz Heinrichstrasse 28/II 8010 Graz Austria katalin.barta@uni-graz.at

## Abstract

Primary amines are crucially important building blocks for the synthesis of a wide range of industrially relevant products. Our comprehensive catalytic strategy presented here allows diverse primary amines from lignocellulosic biomass to be sourced in a straightforward manner and with minimal purification effort. The core of the methodology is the efficient RANEY® Ni-catalyzed hydrogen-borrowing amination (with ammonia) of the alcohol intermediates, namely alkyl-phenol derivatives as well as aliphatic alcohols, obtained through the two-stage *LignoFlex* process*.* Hereby the ***first stage*** entails the copper-doped porous metal oxide (Cu20PMO) catalyzed reductive catalytic fractionation (**RCF**) of pine lignocellulose into a crude bio-oil, rich in dihydroconiferyl alcohol (**1G**), which could be converted into dihydroconiferyl amine (**1G amine**) in high selectivity using ammonia gas, by applying our selective amination protocol. Notably also, the crude **RCF**-oil directly afforded **1G amine** in a high 4.6 wt% isolated yield (based on lignin content). Finally it was also shown that the here developed Ni-catalysed heterogeneous catalytic procedure was equally capable of transforming a range of aliphatic linear/cyclic primary/secondary alcohols – available from the ***second stage*** of the *LignoFlex* procedure – into their respective primary amines.

## Abstract 2

A highly efficient RANEY® Ni-based amination methodology using ammonia that is capable of converting lignocellulose-derived alcohol intermediates into their corresponding primary amines in high selectivity, following hydrogen borrowing methodology.

## Graphical abstract

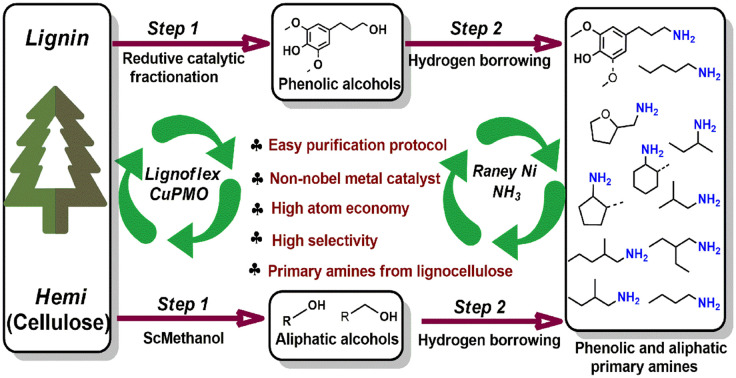

## 1. Introduction

Primary amines hold vast importance for the manufacturing of a wide range of industrially relevant compounds, among which are polymers and pharmaceuticals.^1–5^ Their efficient and sustainable synthesis from widely available alcohols that may be obtained from renewable lignocellulosic biomass and this preferentially by direct coupling with ammonia as an abundant nitrogen source, and utilizing heterogeneous catalysts, is a highly desired objective.^6–9^

An attractive, highly atom-economic method for the direct conversion of alcohols into their respective amines is *via* the “hydrogen borrowing” strategy. Typically such procedure comprises three consecutive stages,^10–12^ namely a) the dehydrogenation of a alcohol to its corresponding carbonyl compound, b) imine formation by reaction of the carbonyl intermediate with the amine reaction partner, accompanied with the release of water as only side-product, and c) reduction of the imine to the desired amine product, using the hydrogen equivalents ‘borrowed’ in the first step. The dehydrogenation and hydrogenation steps are mediated by a suitable transition metal catalyst. In fact, the challenge of finding such suitable catalytic systems to accomplish efficient ‘hydrogen-borrowing’ transformations in high selectivity, especially when using ammonia, has been met by a number of research groups.^5–12^ Exemplary in this matter are the use of Ni/SiO_2_–Al_2_O_3_,^13^ Ni/θ-Al_2_O_3_ (or Ni/γ-Al_2_O_3_),^14^ Ni/CaSiO_3_ (ref. 15) NiAl hydrotalcite,^16^ PdCo/CeO_2_,^17^ and Ru–MgO/TiO_2_ (ref. 18) as heterogeneous catalytic agents. However, while these hydrogen-borrowing catalysts tend to display great activity/selectivity for the amination of aliphatic alcohols, they are markedly less performing in the amination of lignin-derived substituted primary alcohols also comprising an phenol moiety, prone to preferential coordination, especially with supported metal catalysts comprising basic supports.^19^ In parallel, notable cases of the direct reductive amination of phenols to cyclohexylamines have been reported by the De Vos' group.^20,21^

Reductive catalytic fractionation (**RCF**) has recently emerged as a powerful tool^22–25^ to the depolymerization of lignin into lignin-oil that contains aromatic monomers in high selectivity. However, due to the high purification cost and the tedious downstream processing, commercializing of these monomer-rich lignin oils has proven very challenging^26,27^ and targeting higher value products offers to be a beneficial mitigation strategy.^28–30^

**Fig. 1:**
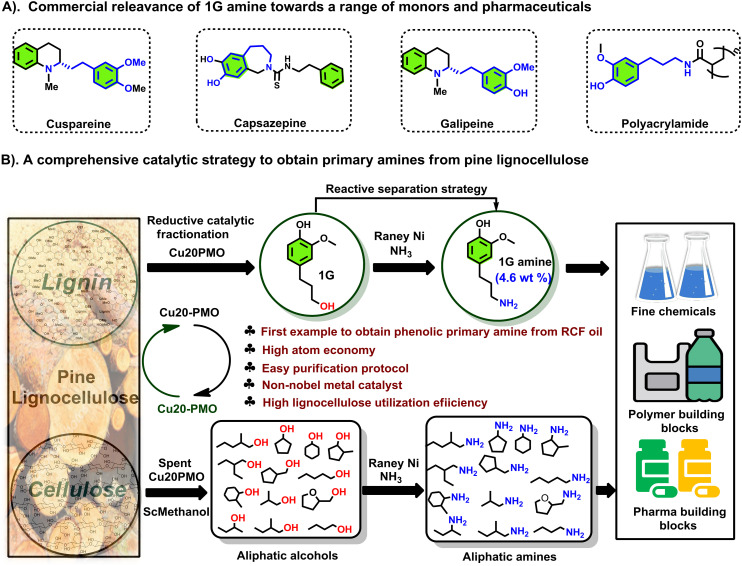
Strategy for the formation of primary amines from both cellulose- and lignin-derived aromatic/aliphatic alcohols, and this with specific attention to real life applications.

We have previously reported on the flexible use of Cu20PMO^28,31,32^ in a unique two stage catalytic lignocellulose disassembly process. In the first step, a reductive catalytic fractionation is performed at 140–180 °C, to deliver lignin-oil particularly rich (up to 90% selectivity) in dihydroconyferyl alcohol (**1G**).^28^ In the second step, the solid residues mainly consisting of cellulose(s) and unreacted lignin, were converted to a mixture of aliphatic alcohols in supercritical methanol, whereby the reducing equivalents for these processes originated from the solvent upon reforming.^33,34^ Strikingly, both these catalytic steps have delivered aliphatic alcohols, which are ideally suited for the development of sustainable amination protocols by the borrowing hydrogen strategy. As a central and representative example, we attempted the direct conversion of dihydroconiferyl alcohol (**1G**) to dihydroconiferyl amine (**1G amine**), an interesting building block for the making of polymeric materials^35^ as well as the synthesis of a range of pharmaceuticals (*e.g.* cuspareine,^36^ capsazepine,^37^ galipeine^36^) (Fig. 1A). This is a particularly challenging transformation as a) the obtained primary amine is prone to overalkylation, that way creating a secondary, or tertiary amine and b) dehydrogenated **1G** (= the corresponding aldehyde product of the first step of the hydrogen borrowing procedure) is susceptible to decarbonylation, that way forming undesired 4-ethyl guaiacol.^28^

Notably, the here developed heterogeneous catalytic procedure using RANEY® Ni as catalyst delivered excellent product selectivities. This was further validated in the amination of two other lignin-derived phenolic primary alcohols, namely dihydrosinapyl alcohol (**1S**) and 4-(3-hydroxypropyl)phenol (**1H**) as well as a range of aliphatic alcohols (primary and secondary), available from the second step of the *LignoFlex* procedure.^28^ In summary, this work presents a catalytic strategy for the depolymerization and upgrading of pine lignocellulose to primary amines (Fig. 1B).

## 2. Experimental

### 2.1 Catalyst preparation

The Cu20PMO catalyst was prepared according to our previously reported procedure.^28^ In a typical procedure, a solution containing AlCl_3_·6H_2_O (12.07 g, 0.05 mol), Cu(NO_3_)_2_·2.5H_2_O (6.98 g, 0.03 mol) and MgCl_2_·6H_2_O (24.4 g, 0.12 mol) in deionized water (200 mL) was dropwise added to a solution containing Na_2_CO_3_ (5.30 g, 0.05 mol) in water (300 mL) at 60 °C under vigorous stirring. The pH value was always kept between 9 and 10 by addition of small portions of a 1 M solution of NaOH. The mixture was vigorously stirred at 60 °C for 72 h. After cooling to room temperature, the light blue solid was filtered and resuspended in a 2 M solution of Na_2_CO_3_ (300 mL) and stirred overnight at 40 °C. The catalyst precursor was filtered and washed with deionized water until chloride free. After drying the solid for 6 h at 100 °C followed by the calcination at 460 °C for 24 h in air, 9.5 g of Cu20PMO was obtained.

### 2.2 Characterizations

Gas Chromatography (**GC**) was used for products identification as well as determination of conversion and selectivity values.

Products identification was performed by GC-MS (5975C MSD) equipped with an HP-5MS column, and helium as carrier gas. The temperature program started at 50 °C for 5 min, heated by 10 °C min^−1^ to 325 °C and held for 5 min. Conversion and products selectivity were determined by GC-FID (Shimadzu Agilent 8890 GC) equipped with an HP-5MS column using nitrogen as carrier gas. Nuclear Magnetic Resonance (**NMR**) spectroscopy: ^1^H, and ^13^C NMR spectra were recorded on a Bruker Avance III 300 MHz (300 and 75 MHz, respectively) and 2D NMR spectra were recorded on a Bruker Avance III 700 MHz with Cryoplatform and a 5 mm Triple-Resonance cryoprobe (700 and 175 MHz, respectively). ^1^H,^13^C NMR and 2D NMR spectra were recorded at RT. Chemical shift values are reported in ppm with the solvent resonance as the internal standard (CDCl_3_: 7.26 for ^1^H, 77.0 for ^13^C; CD_3_OD: 3.31 for ^1^H, 49.0 for ^13^C; DMSO-d_6_: 2.50 for ^1^H, 39.5 for ^13^C). Data are reported as follows: chemical shifts, multiplicity (s = singlet, d = doublet, t = triplet, q = quartet, br. = broad, m = multiplet), coupling constants (Hz), and integration.

### 2.3 Method

#### Reductive catalytic fractionation of lignocellulosic biomass

The mild depolymerization of pine lignocellulose was carried out in a high-pressure Parr autoclave equipped with an overhead stirrer. Typically, the autoclave was charged with 0.4 g of Cu20PMO catalyst, 2 g of pine lignocellulose and methanol (20 mL) as a solvent. The reactor was sealed and pressurized with H_2_ (40 bar) at room temperature. The reactor was heated to 180 °C and stirred at 400 rpm for 18 h. After completion of the reaction, the reactor was cooled to room temperature. Then 0.1 mL solution was collected through a syringe and injected to GC-MS or GC-FID after filtration through a PTFE filter (0.45 μm). The solid was separated from the solution by centrifugation and subsequent decantation and additionally washed with methanol (3 × 20 mL). The methanol washings were combined in a round bottom flask and the solvent was removed *in vacuo*. The crude product was dried in a desiccator *in vacuo* overnight and was further used as specified below.

#### Fractionation procedure

To the obtained crude aromatic bio-oil, EtOAc (20 mL) was added and it was stirred overnight at room temperature, which resulted in the precipitation of brownish colored solid (31 mg). The suspension was then transferred into a 20 mL centrifuge tube. The solids were separated by centrifugation and decantation and additionally washed with EtOAc (3 × 20 mL) and dried *in vacuo* until constant weight. The EtOAc washings were combined in a separating funnel and were washed with small amount of saturated NaHCO_3_ (1 × 10 mL) and brine (2 × 10 mL) and the organic phase was dried over anhydrous MgSO_4_. After filtration, the whole organic phase was removed *in vacuo* to give yellow crude product (EtOAc extracts, 148 mg) for further use, as specified below.

#### Reductive amination of model alkyl-phenols and simpler aliphatic alcohols

The direct catalytic amination of alcohols into the corresponding amines was performed in 10 mL high pressure autoclave equipped with magnetic stirring bar. Typically, a 4 mL vial was charged with 0.5 mmol substrate, 100 mg RANEY® Ni catalyst and 2.5 mL *t*-amyl alcohol as solvent. Then the vial was placed inside an autoclave and the reactor was subsequently sealed and pressurized with 7 bar NH_3_. The reactor was heated at the indicated temperature, typically 120–160 °C and stirred at 400 rpm for 18 h. After completion of the reaction, the reactor was cooled down to RT. Then, 0.1 mL solution was collected through a syringe and injected to GC-MS or GC-FID after filtration through a PTFE filter (0.45 μm). Then the mixture was resolubilized in 0.5 ml *t*-amyl alcohol and 20 mL diethyl ether, followed by the addition of 2 N HCl in diethyl ether. The solid was then isolated by filtration and washed with diethyl ether (3 × 20 mL) to provide the pure amine salts.

## 3. Results and discussion

### 3.1 Catalytic amination of lignin-derived 1G to 1G amine

**Fig. 2:**
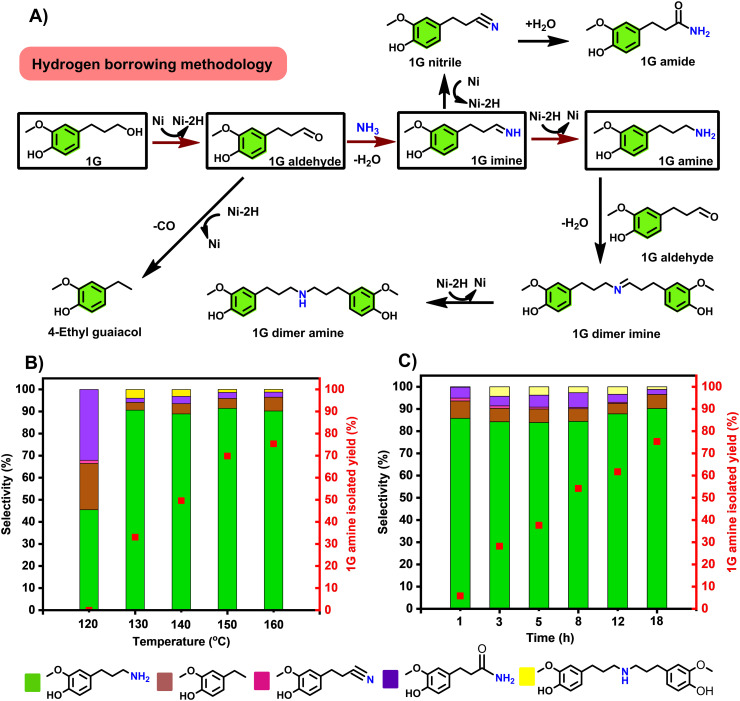
A) A proposed reaction network detailing desired main reactions towards the **1G amine** and typical expected side-reactions, in relation to the experimentally observed major and minor products; B) influence of the reaction temperature on the reaction selectivity and the **1G amine** isolated yield. Reaction time: 18 h; C) influence of the reaction time on reaction selectivity and the **1G amine** isolated yield. Reaction temperature: 160 °C; general reaction conditions: 0.5 mmol **1G**, 100 mg RANEY® Ni, 2.5 mL *t*-amyl alcohol, 120–160 °C, 1–18 h, 7 bar NH_3_. Selectivity and conversion were determined by GC-FID based on the peak area (see calculation methods in supplementary information). **1G amine** was isolated as its HCl salt. For numerical values see Tables S2 and S3.†

Our first goal was to accomplish the highly selective amination of the 4-(3-propanol) phenol derivatives, products of reductive catalytic fractionation (**RCF**) of lignocellulose, directly with ammonia gas. While this transformation appears straightforward, achieving high selectivity towards the primary amine is very challenging, due to a series of side reactions that may take place under the operating conditions, as summarized on Fig. 2A. An ideal catalytic system should favour alcohol dehydrogenation and facilitate imine-to amine reduction, both crucial steps in the hydrogen borrowing sequence. With this goal in mind, we have evaluated a range of commercially available heterogeneous catalysts towards the direct amination of **1G** to **1G amine**. Here, *t*-amyl alcohol was selected because it has been proved effective as a solvent towards the production of primary amines.^19,38^ Surprisingly, very low **1G** conversions (typically under 10%) were observed with a range of catalysts at 150 °C, notably Pd/C, Pt/C, Rh/C, Ru/Al_2_O_3_, Ni/SiO_2_–Al_2_O_3_, Ni/SiO_2_, indicating low efficiency of alcohol dehydrogenation with these catalysts, under these conditions. Notably, in these runs, rather the corresponding **1G nitrile** was seen as main product in no less than 90% selectivity (Table S1,† entries 1–6), which could be attributed to higher dehydrogenation activity than hydrogenation of **1G imine** over these catalysts in the absence of H_2_ pressure. Best selectivity towards **1G nitrile** (up to 99%) was observed with group VIII containing heterogeneous catalysts – notably Pd/C, Pt/C, Rh/C and Ru/Al_2_O_3_ (Table S1,† entries 1–4).

The formation of **1G nitrile** likely involved **1G imine** dehydrogenation which could also undergo subsequent hydration to form **1G amide** – the other side product.^39^ With the base metal catalysts Ni/SiO_2_–Al_2_O_3_ and Ni/SiO_2_, the 4-ethylguaiacol was the dominant side product (Table S1,† entries 5 and 6). The latter compound derives from the decarbonylation of **1G aldehyde**.^28^ The inactivity of supported metal catalyst was possibly attributed to preferred adsorption of phenol moiety of **1G** on supports such as Al_2_O_3_ and SiO_2_, leaving aliphatic –OH moiety uncoordinated.^40^

Interestingly, in sharp contrast to all the other catalysts tested, the application of RANEY® Ni gave rise to the formation of **1G amine** in 91.4% selectivity at a conversion level of 87.6% (Table S1, entry 7, Fig. S1†). From this reaction mixture **1G amine** could be obtained as its HCl salt in up to 70% isolated yield. Overalkylation of **1G amine** to **1G dimer amine** was only detected in very small amounts (Table S1,† entry 7). The high catalytic reactivity was mainly ascribed to the fact that RANEY® Ni is highly efficient in a range of hydrogen transfer transformations, including amination by hydrogen-borrowing reactions.^41^ It has to be noticed that using atmospheric pressure of NH_3_ shifted towards the production of 4-ethyl guaiacol, with a selectivity of >99%. (Table S4†) Therefore this catalyst system was selected for detailed optimization over a wide temperature range (120–160 °C), the results having been summarized in Fig. 2B. A high **1G amine** selectivity was obtained in all these runs. As though the lower temperature range represented incomplete conversions (Tables S2 and S3†), the isolated **1G amine** yield was found to increase with the applied reaction temperature, reaching a maximum of 75.8% at 160 °C (Fig. 2B). While the **1G amine** selectivity was found independent of the applied reaction time, the isolated **1G amine** yield increased with the reaction time (Fig. 2C). In addition, catalyst loading was also investigated (Table S5†). It was found the optimal catalyst loading is 100 mg, which gives the best **1G** conversion of 94.6% and isolated yield (75.8%) to **1G amine**. While using decreased catalyst loading only lower the **1G** conversion, proceeding 25.8 and 66.2%, respectively. Upscaling the reaction using 500 mg **1G** led to slightly decreased catalytic reactivity, with a conversion of 86.6% and 80.5% selectivity (Table S6†).

**Fig. 3:**
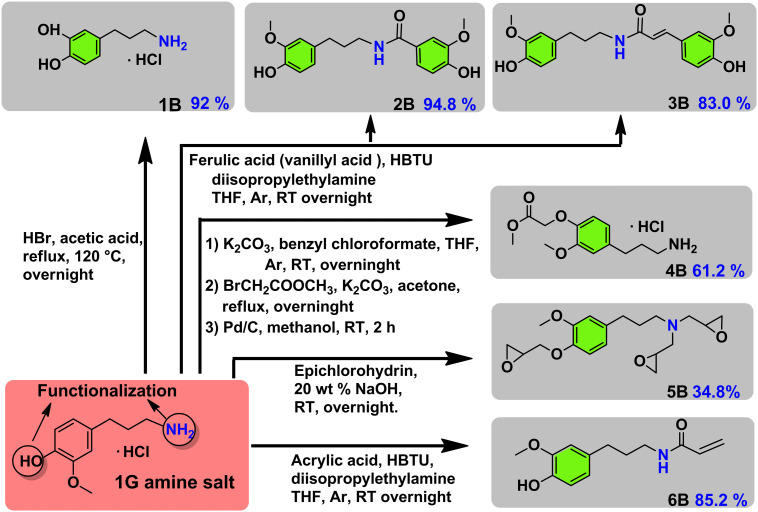
Potential usage of **1G amine** as a synthetic building block for a range of pharmaceutical and polymeric building blocks.

**Fig. 4:**
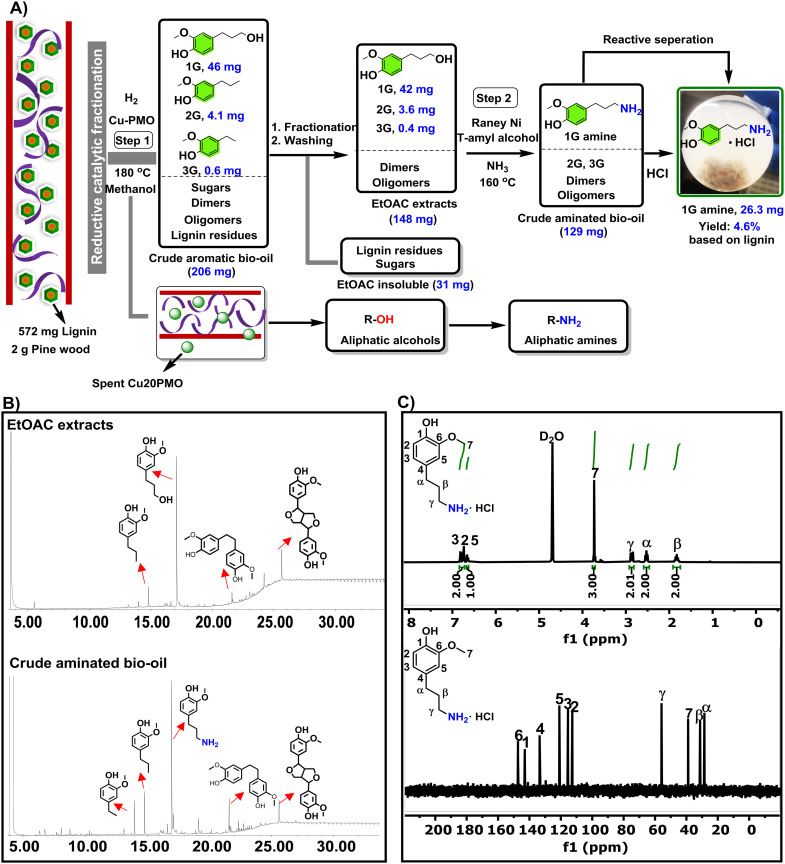
Methodology to obtain **1G amine** from pine lignocellulose. A) Established protocol for the **1G amine** formation from pine lignocellulose; step 1: 2 g pine lignocellulose, 400 mg Cu20PMO, 20 mL methanol, 180 °C, 18 h; step 2: 200 mg RANEY® Ni, 2.5 ml *t*-amyl alcohol, 7 bar NH_3_, 160 °C, 18 h; B) GC-FID traces of the EtOAc extracts of the original crude RCF bio-oil and the crude aminated bio-oil C) ^1^H NMR and ^13^C NMR spectra of the **1G amine** salt isolated from the crude aminated lignin oil using HCl.

**Fig. 5:**
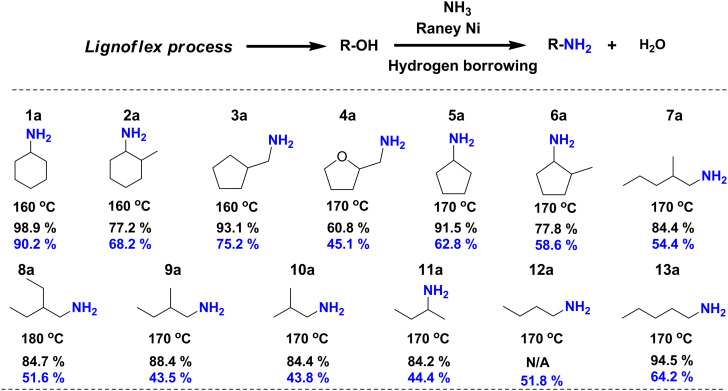
Catalytic direct amination of polysaccharide-derived aliphatic primary/secondary linear/cyclic alcohols to their corresponding primary/secondary amines. All aliphatic alcohols present here were separately treated by catalytic amination procedure to corresponding primary amines. Reaction conditions: 0.5 mmol substrates, 100 mg RANEY® Ni catalyst, 2.5 mL *t*-amyl alcohol, 7 bar NH_3_, 160–180 °C, 18 h. Yield: the black numbers refer to the yield determined by GC-FID based on the peak area (see calculation methods in ESI†) while the blue numbers relate to isolated yields. For compound **12a**, the GC-FID peak is overlapped with solvent peak.

In a further advancement we studied the synthetic possibilities of **1G amine** towards the formation of important pharmaceutical and polymeric building blocks (see Fig. 3 for an overview) and proposed suitable future applications, inspired by recent literatures. First, demethylation of **1G amine** to **dopa 1G amine** (Fig. 3-**1B**) was achieved in 92% isolated yield. The latter compound is an essential building block to the medicine capsazepine^37^ (see also Fig. 1A). Next, **1G amine** was further reacted with vanillyl acid and ferulic acid which can be in principle sourced from lignin, that way providing for a range of polymerizable bio-based bisguaiacols^42,43^ (Fig. 3-**2B** and **3B**). The respective isolated yields of **2B** and **3B** are 94.8% and 83.0%. Furthermore, the functionalization of **1G amine** with methyl bromoacetate gave a potential polyamide building block **4B**,^44^ while the reaction of **1G amine** with epichlorohydrin yields a multiepoxy containing monomer **5B** obtained in 34.8% isolated yield (Fig. 3-**5B**), which is highly suitable for the creation of epoxy resins. Alternatively, functionalization of **1G amine** with acrylic acid yields a suitable monomer (**6B**) for (controlled) radical and cationic polymerization.^35^ The isolated yield of **6B** was 85.2%.

Next to **1G amine** formation, the usage of RANEY® Ni as a hydrogen borrowing amination catalyst was also tested, under optimal reaction conditions, in the amination of two other lignin-derived platform molecules that may originate from the **RCF** of hardwood, **1S** and **1H**. This leads respectively to the formation of dihydrosinapyl amine (**1SA**) and 4-(3-aminopropyl)phenol (**1HA**), two structural motives omnipresent in pharmaceuticals (*e.g.* trimethoprim).^45^ In comparing Table 1, it can be seen that the presence an additional methoxy group *vis-à-vis***1G**, as in compound **1S**, leads to a marked drop in conversion, going from 94.6% for **1G** to 66.4% for **1S**. Importantly though, the selectivity for **1S amine** is maintained at 91.2%, slightly higher than the one observed for **1G amine** (90.2%). Conversely, with compound **1H** as substrate near quantitative conversion is obtained (99.5%) at a similarly high selectivity (89.4%) as observed with the formation of **1G amine**. With **1S** the conversion level could be further increased from 66.4% (seen at 160 °C) to 89.2% by using a somewhat higher reaction temperature of 180 °C, with only a slight decrease in **1S amine** selectivity (89.4%). Isolated yields of the HCl salts of **1S amine** and **1H amine** were 64.5% and 53.4% respectively.

**Table 1:** Investigations of reactivity for catalytic amination of **1S** and **1H** with ammonia over RANEY® Ni catalyst^a^

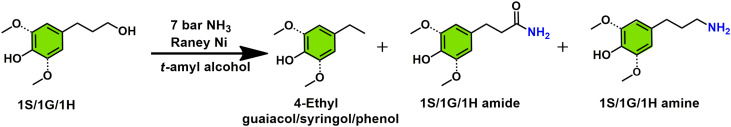							
Substrate	*T* (°C)	Conv. (%)	Sel. (%)			GC yield (%)	Isolated yield (%)
			4-Ethyl/guaiacol syringol	**1S**/**1G**/**1H amide**	**1S**/**1G**/**1H amine**		
**1H**	160	99.5	4.1	6.4	89.4	90.0	53.4
**1G**	160	94.6	5.0	2.5	90.2	85.3	75.8
**1S**	160	66.4	3.2	1.8	91.2	60.6	32.4
**1S**	180	89.2	3.5	2.2	89.4	79.7	64.5

a Reaction conditions: 0.5 mmol substrate, 100 mg RANEY® Ni, 2.5 mL *t*-amyl alcohol, 18 h, 7 bar NH_3_. Selectivity, conversion and yield were determined by GC-FID based on the peak area (see calculation methods in ESI†).

### 3.2 Catalytic upgrading of native-lignin into 1G amine

Next, we attempted to apply our RANEY® Ni-based amination protocol developed on model compounds, directly to the crude depolymerized lignin oil obtained by **RCF** of pine lignocellulose over previously developed procedure using 2 g pine lignocellulose and Cu20PMO as catalyst at reaction conditions (2 g pine, 0.4 g Cu20PMO, 20 mL methanol, 40 bar H_2_, 180 °C, 18 h). When applying the here developed amination procedure directly to crude aromatic bio-oil (see Fig. 4A), consisting mainly of monomers (**1G**, 46 mg, **2G**, 4.1 mg, **3G**, 0.6 mg) but also a range of other compounds typical for such bio-oil such as dimers, oligomers and sugars,^28,46^ no amination could be observed. This is tentatively attributed to the presence of oligomers/polysaccharides and a small amount of organic acids.^46,47^ To alleviate these problems the crude aromatic bio-oil was extracted with ethyl acetate (EtOAc), followed by a treatment with small amount of NaHCO_3_/brine, that way yielding a purified bio-oil (EtOAc extracts) rich in **1G** and lesser amounts of **2G** and **3G**, as characterized by GC-FID (Fig. 4B). Gratifyingly in applying the amination protocol to such EtOAc extracts, **1G** amination was found to proceed cleanly (Fig. 4B). Moreover, inherent purification of **1G amine** was found possible by precipitation as its HCl salt, characterized by ^1^H NMR and ^13^C NMR (see Fig. 4C), giving a 4.6 wt% isolated yield on lignin basis.

### 3.3 Direct amination aliphatic alcohol intermediates originating from cellulose residues

As the second step of the *LignoFlex* procedure converts the remaining polysaccharides over Cu20PMO in supercritical methanol, into a mixture consisting of predominantly aliphatic primary/secondary linear/cyclic alcohols,^28^ our heterogeneous catalytic amination methodology was also evaluated on individual model compounds representing the main compounds of these lignocellulose-derived product mixtures. As can be inferred from Fig. 5, most depicted amination reactions gave GC determined yields larger than 80%, and isolated yields between 40–70%. It can further be seen that steric hindrance adjacent to the alcohol moiety tends to decrease the obtained yield (1a*versus*2a, 5a*versus*6a, 7a*versus*13a). Also, the substitution of a cyclopentane ring for a tetrahydrofuran one markedly influenced the obtained amine yield, reaching 93.1% and 60.8%, respectively. No distinct difference in reactivity between primary and secondary aliphatic alcohols could be discerned.

We note, that a gap between isolated yield and GC yield is observed especially for linear/branched short chain aliphatic amine salts (*e.g.*7a–13a) compared to a lower than 20% gap for amine products bearing cyclic aliphatic or aromatic moieties. We attribute this to the more challenging or incomplete isolation of the respective amine salts, rather than selectivity reasons.

The rationale of using single model alcohol compounds over artificial/real mixtures of alcohols lies in the fact that future alcohol producing biorefineries – such as those based on the *Lignoflex* procedure – would be able to separate pure product streams by distillation of such alcohol mixtures, before converting them to amines. This is rationalized by the fact that 1) mixtures of amines have lower boiling points as well as a narrower spread of the boiling points than mixtures of alcohols and 2) in converting alcohol mixtures to their corresponding amines one often creates undesired azeotropes. In these respects it is also noteworthy that industrial amine separation revolves around multi-distillation trains, extensive extraction steps and recycling of various streams, which all comes at high energy and materials costs.^48^ Nonetheless, the direct application of our method to alcohol mixtures to create mixtures of amines for certain specific applications (*e.g.* surfactants, lubricants) may also be an industrially relevant scenario.

## 4. In conclusion

We have developed a RANEY® Ni-based amination methodology capable of transforming lignin-derived phenolic primary alcohols with differing aromatic substitution patterns into their corresponding primary amines in high selectivities (∼90%) at high conversions (>90%) while keeping the phenol moiety intact. Moreover, this was achieved using an atom efficient hydrogen borrowing approach, the only side product being water. Furthermore, provided a first extraction step, RANEY® Ni was found capable to aminate a bio-oil rich in **1G** (as obtainable through the *LignoFlex* procedure) yielding the corresponding amino-alkyl phenol (**1G amine)** in 4.6% isolated yield on lignin basis. To the best of our knowledge the capability of aminating phenolic primary alcohols obtained from real RCF oil directly using ammonia, with a heterogeneous catalyst, and following an atom economic hydrogen borrowing methodology, has not yet been reported on. Furthermore, by providing relevant synthetic pathways, we have concisely shown that such phenolic primary amines can serve as suitable pharmaceutical and polymeric building blocks. Moreover, applying the developed amination methodology to a range of model aliphatic primary/secondary linear/cyclic alcohols, such as those obtained from the *LignoFlex* process, we demonstrated the applicability of the same method on converting cellulose-derived platform chemicals into primary amines in high overall yield.

Overall, the capability to derive a broad range of amines from different biomass constituents: lignin as well as (hemi)cellulose, using a non-noble heterogeneous catalyst, an atom economic hydrogen borrowing methodology, and involving minimal purification efforts (minimal extraction and facile isolation), holds potential to broadening the scope of sustainable biorefineries producing a range of value-added amines.

## Conflicts of interest

There are no conflicts to declare.

## Supplementary Material

CY-012-D2CY00864E-s001
