# Supplementary material for: Primary amines from lignocellulose by direct amination of alcohol intermediates, catalyzed by RANEY® Ni
Source: Catal Sci Technol. 2022 Aug 19;12(19):5908–16. doi: 10.1039/d2cy00864e (PMC9528992; doi:10.1039/d2cy00864e)
Supplement: CY-012-D2CY00864E-s001 [file CY-012-D2CY00864E-s001.pdf]

Electronic Supplementary Material (ESI) for Catalysis Science & Technology.
This journal is © The Royal Society of Chemistry 2022

Supplementary information

Primary amines from lignocellulose by direct amination of alcohol intermediates, catalyzed by Raney Ni

Xianyuan Wu[1], Mario De bruyn[2], and Katalin Barta[1,2*]

[1] Stratingh Institute for Chemistry, University of Groningen, Nijenborgh 4, 9747 AG, The Netherlands

[2] Institute for Chemistry, University of Graz, Heinrichstrasse 28/II, 8010, Graz, Austria

*Correspondence: Katalin Barta (k.barta@rug.nl, katalin.barta@uni-graz.at)

Table of contents

1. **Calculation of conversion, selectivity and yield** 3

2. **Additional supporting information to the identification of the optimal reaction conditions for the Raney nickel catalyzed 1G amination** 3

3. **Assigned GC-FID traces for the Raney nickel catalyzed 1G amination** 5

4. **Supporting ^{1}H and ^{13}C NMR spectra** 6

5. **List of the spectral and spectrometric data of the isolated compounds** 30

1. Calculation of conversion, selectivity and yield

<u>For the calculation of conversion based on GC:</u>

Conversion (%) = $\frac{Sum\ of\ all\ the\ product\ peak\ areas}{[(Sum\ of\ all\ the\ product\ peak\ areas)\ +\ the\ peak\ area\ of\ the\ remaini\text{[illegible]}} \times$ 100%

<u>For the calculation of selectivity based on GC:</u>

Selectivity (%) = $\frac{The\ peak\ area\ of\ the\ target\ product}{Sum\ of\ all\ the\ product\ peak\ areas} \times$ 100%

<u>For the calculation of yield based on GC:</u>

Yield (%) =selectivity $\times\ conversion$

Isolated yield (%) is based on the effective dry weight of an HCl salt of target amine

2. Additional supporting information to the identification of the optimal reaction conditions for the Raney nickel catalyzed 1G amination

Table S1. Performance of a range of commercially available heterogeneous catalysts in the catalytic amination of **1G** to **1G amine** using ammonia.[a]

Entry	catalyst	Conv.[b] (%)	Sel. (%)[b]					GC/isolated Yield[b] (%)
			**1G amine**	**4-Ethy guaiacol**	**1G nitrile**	**1G amide**	**1G dimer amine**	
1	Pd/C	2.7	-	-	98.8	1.2	-	-
2	Pt/C	1.8	-	-	97.6	2.4	-	-
3	Rh/C	1.2	-	-	98.5	1.5	-	-
4	Ru/Al_2O_3	0.8	-	-	98.5	1.5	-	-
5	Ni/SiO_2-Al_2O_3	6.6	-	7.2	92.8	-	-	-
6	Ni/SiO_2	5.9	-	6.7	93.3	-	-	-
7	Raney Ni	87.6	91.4	4.5	-	1.0	1.4	74.7 (69.8)[c]

[a]. Reaction conditions: 0.5 mmol **1G**, 100 mg catalyst, 2.5 mL *t*-amyl alcohol, 150 °C, 7 bar NH_3, 18 h
[b]. Conversion, selectivity, and yield were all determined by GC-FID
[c]. Isolated yield in parentheses

Table S2. Influence of the reaction temperature on the catalytic amination of **1G** to **1G amine** using ammonia.[a]

Entry	Temp. (°C)	Conv.[b] (%)	Sel. (%)[b]					Isolated yield[c] (%)
			**1G amine**	**4-ethy guaiacol**	**1G nitrile**	**1G amide**	**1G dimer amine**	
1	120	1.4	45.2	21.0	1.4	32.4	-	0
2	130	44.6	90.6	3.5	-	1.9	4.0	33.1
3	140	73.7	88.9	4.7	-	3.3	3.1	49.6
4	150	87.6	91.4	4.5	-	2.7	1.4	69.8
5	160	94.6	90.2	6.3	-	2.3	1.2	75.8

[a]. Reaction conditions: 0.5 mmol **1G**, 100 mg Raney Ni, 2.5 mL *t*-amyl alcohol, 120-160 °C, 7 bar NH_3, 18 h [b]. Conversion and selectivity were all determined by GC-FID

Table S3. Influence of the reaction time on the catalytic amination of **1G** to **1G amine** using ammonia.[a]

Entry	Time (h)	Conv.[b] (%)	Sel. (%)[b]					Isolated yield[c] (%)
			**1G amine**	**4-ethy guaiacol**	**1G nitrile**	**1G amide**	**1G dimer amine**	
1	1	15.9	85.8	7.8	1.3	4.9	0.2	5.8
2	3	45.5	84.2	6.1	1.1	4.3	4.3	28.2
3	5	58.2	83.9	6.1	0.8	5.4	3.8	37.6
4	8	69.5	84.4	5.7	0.6	6.6	2.7	54.2
5	12	86.6	87.8	4.7	0.5	3.6	3.4	61.7
6	18	94.6	90.2	6.3	-	2.3	1.2	75.8

[a]. Reaction conditions: 0.5 mmol **1G**, 100 mg Raney Ni, 2.5 mL *t*-amyl alcohol, 160 °C, 7 bar NH_3, 1-18 h
[b]. Conversion and selectivity were all determined by GC-FID

Table S4. Influence of ammonia pressure on the catalytic amination of **1G** to **1G amine** using ammonia.[a]

Entry	Ammonia pressure (Bar)	Conv.[b] (%)	Sel. (%)[b]					Isolated yield[c] (%)
			**1G amine**	**4-ethy guaiacol**	**1G nitrile**	**1G amide**	**1G dimer amine**	
1	Atmospheric Pressure	70	-	> 99	-	-	-	-
2	7	94.6	90.2	6.3	-	2.3	1.2	75.8

[a]. Reaction conditions: 0.5 mmol **1G**, 100 mg Raney Ni, 2.5 mL *t*-amyl alcohol, 160 °C, atmospheric pressure and 7 bar NH_3, 18 h.
[b]. Conversion and selectivity were all determined by GC-FID

Table S5. Influence of catalyst loading on the catalytic amination of **1G** to **1G amine** using ammonia.[a]

Entry	Catalys loading	Conv.[b] (%)	Sel. (%)[b]					Isolated yield[c] (%)
			**1G amine**	**4-ethy guaiacol**	**1G nitrile**	**1G amide**	**1G dimer amine**	
1	20	25.8	88.6	6.0	-	3.8	1.6	10.7
2	50	66.2	87.8	5.6	-	4.6	2.0	40.3
3	100	94.6	90.2	6.3	-	2.3	1.2	75.8

[a]. Reaction conditions: 0.5 mmol **1G**, 20-100 mg Raney Ni, 2.5 mL *t*-amyl alcohol, 160 °C, 7 bar NH_3, 18 h.
[b]. Conversion and selectivity were all determined by GC-FID

Table S6. Influence of substrate loading on the catalytic amination of **1G** to **1G amine** using ammonia.[a]

Entry	substrate loading (mg)	Conv.[b] (%)	Sel. (%)[b]					Isolated yield[c] (%)
			**1G amine**	**4-ethy guaiacol**	**1G nitrile**	**1G amide**	**1G dimer amine**	
1	500	86.6	80.5	14.4	0	2.8	4.4	55.8

[a]. Reaction conditions: 500 mg **1G**, 500 mg Raney Ni, 8 mL *t*-amyl alcohol, 160 °C and 7 bar NH_3, 18 h.

[b]. Conversion and selectivity were all determined by GC-FID

3. Assigned GC-FID traces for the Raney nickel catalyzed 1G amination

Figure S1. **GC-FID** traces for the Raney nickel catalyzed **1G** amination. Reaction conditions: A) 100 mg Raney nickel catalyst, 0.5 mmol **1G**, 2.5 mL *t*-amyl alcohol, 7 bar NH_3, 160 °C, 3 h. B) 100 mg Raney nickel catalyst, 0.5 mmol **1G**, 2.5 mL *t*-amyl alcohol, 7 bar NH_3, 160 °C, 18 h.

4. Supporting ^{1}H and ^{13}C NMR spectra

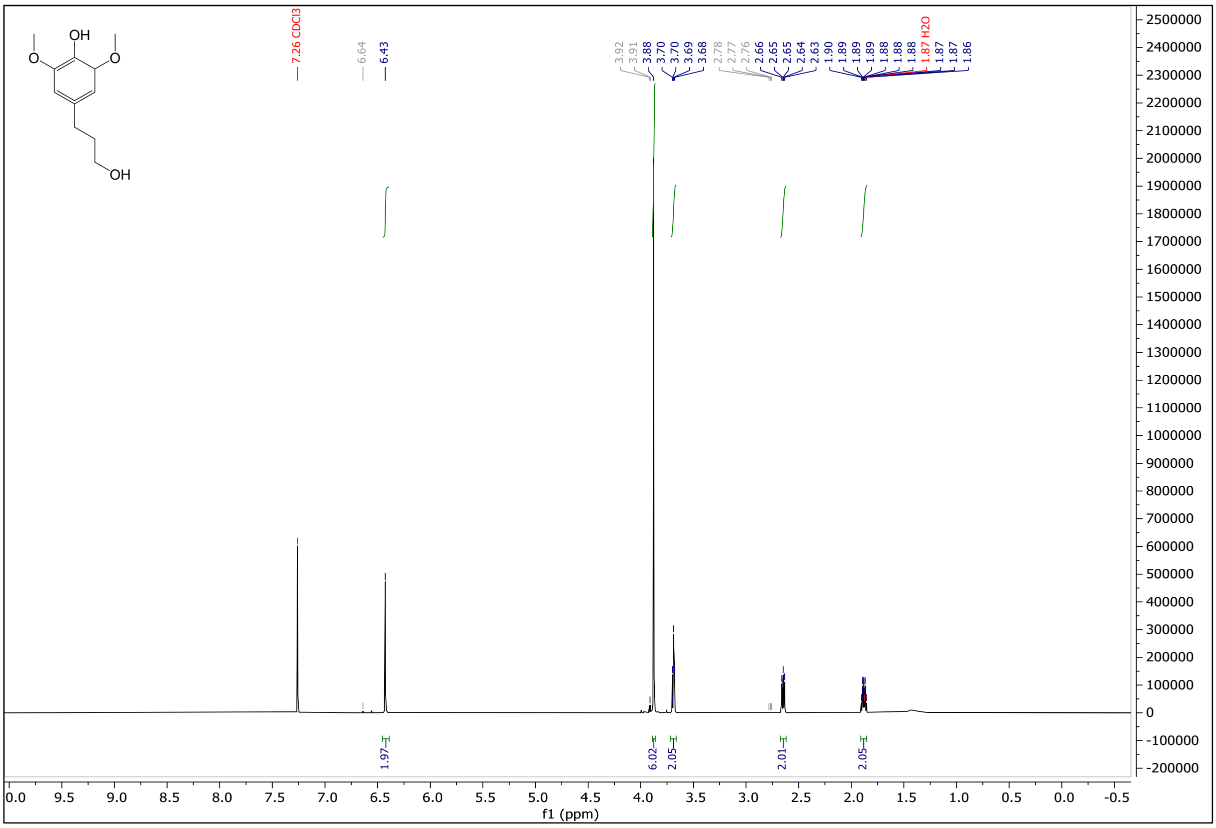

Figure S2. ^{1}H NMR spectrum of **1S**.

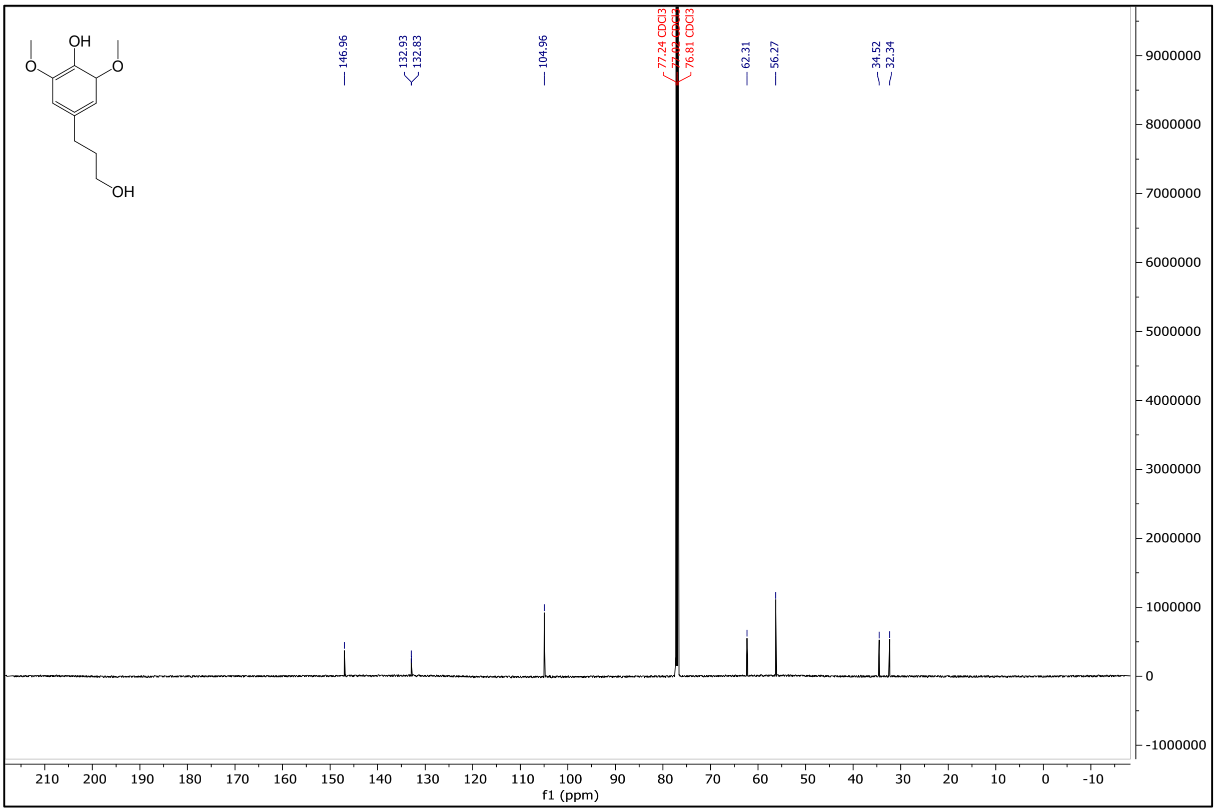

Figure S3. ^{13}C NMR spectrum of **1S**.

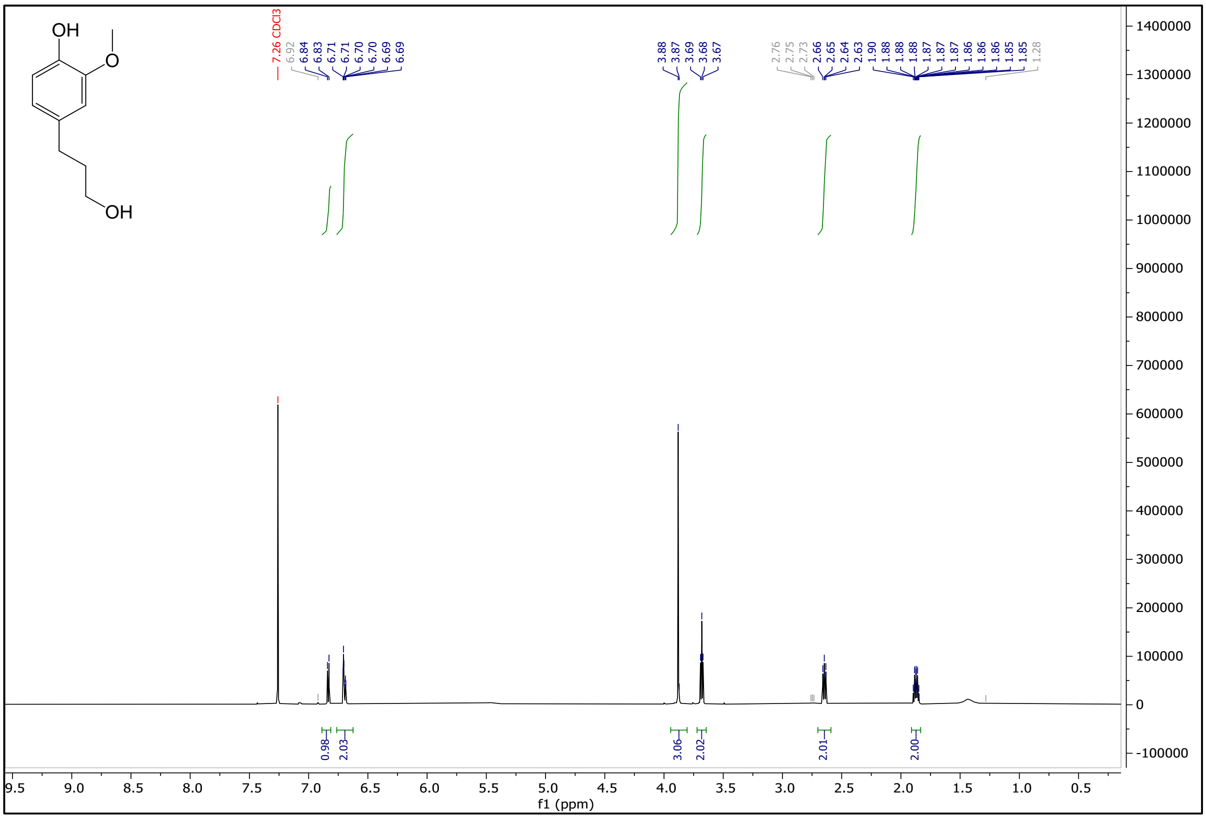

Figure S4. ^{1}H NMR spectrum of **1G**.

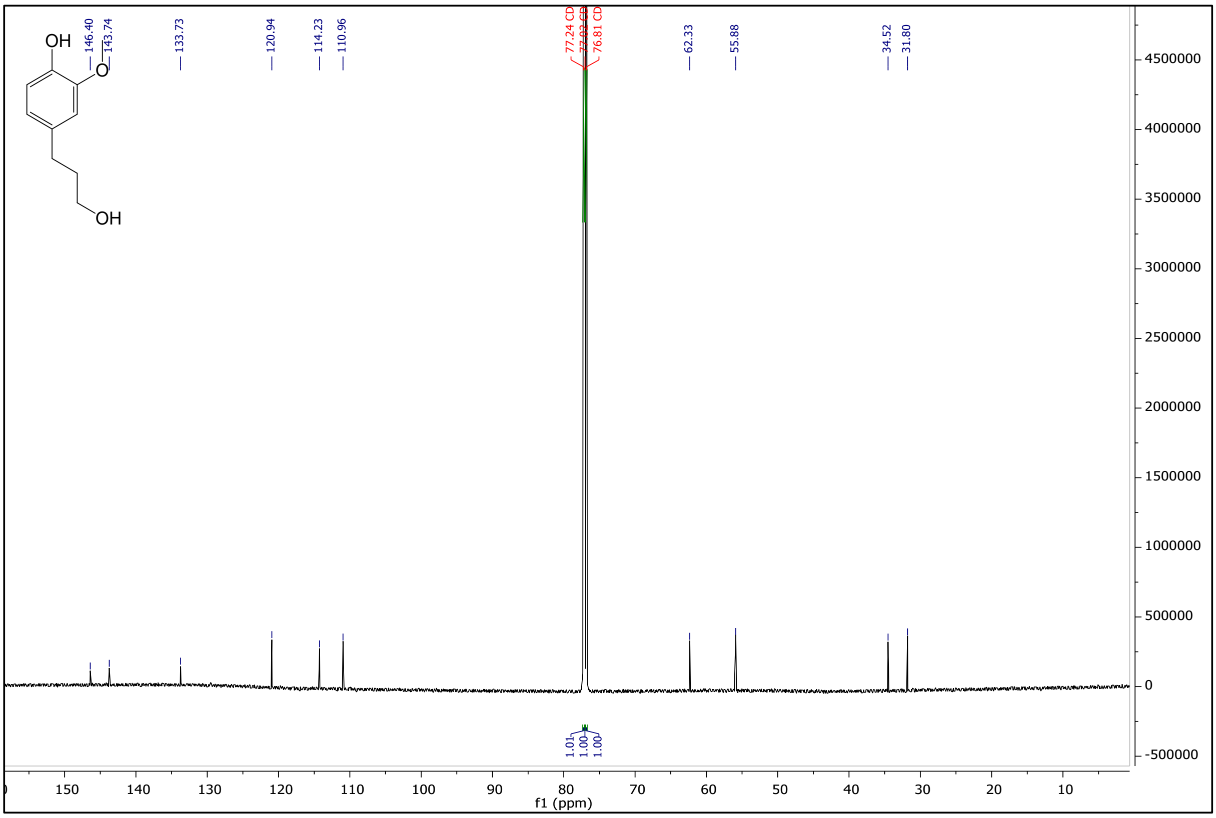

Figure S5. ^{13}C NMR spectrum of **1G**.

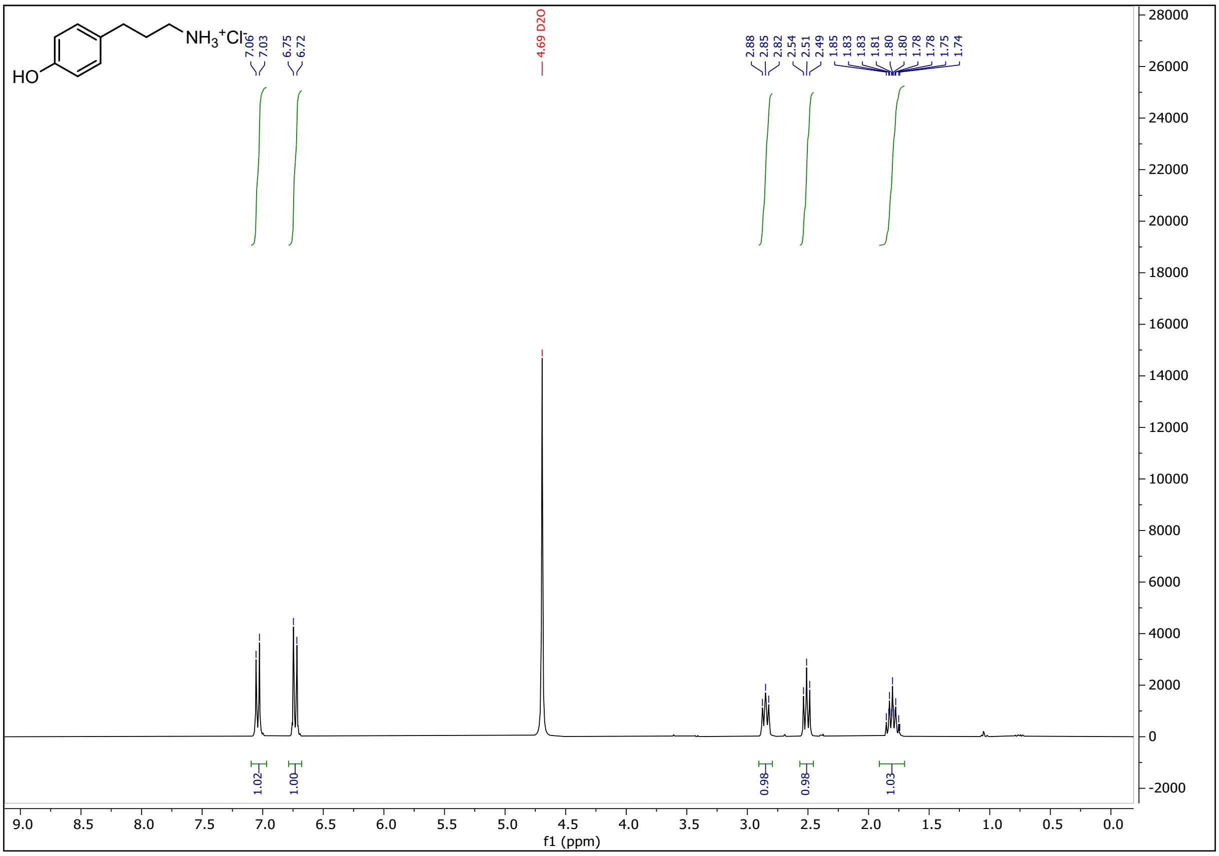

Figure S6. ^{1}H NMR spectrum of **1H amine** (as the HCl ammonium salt)

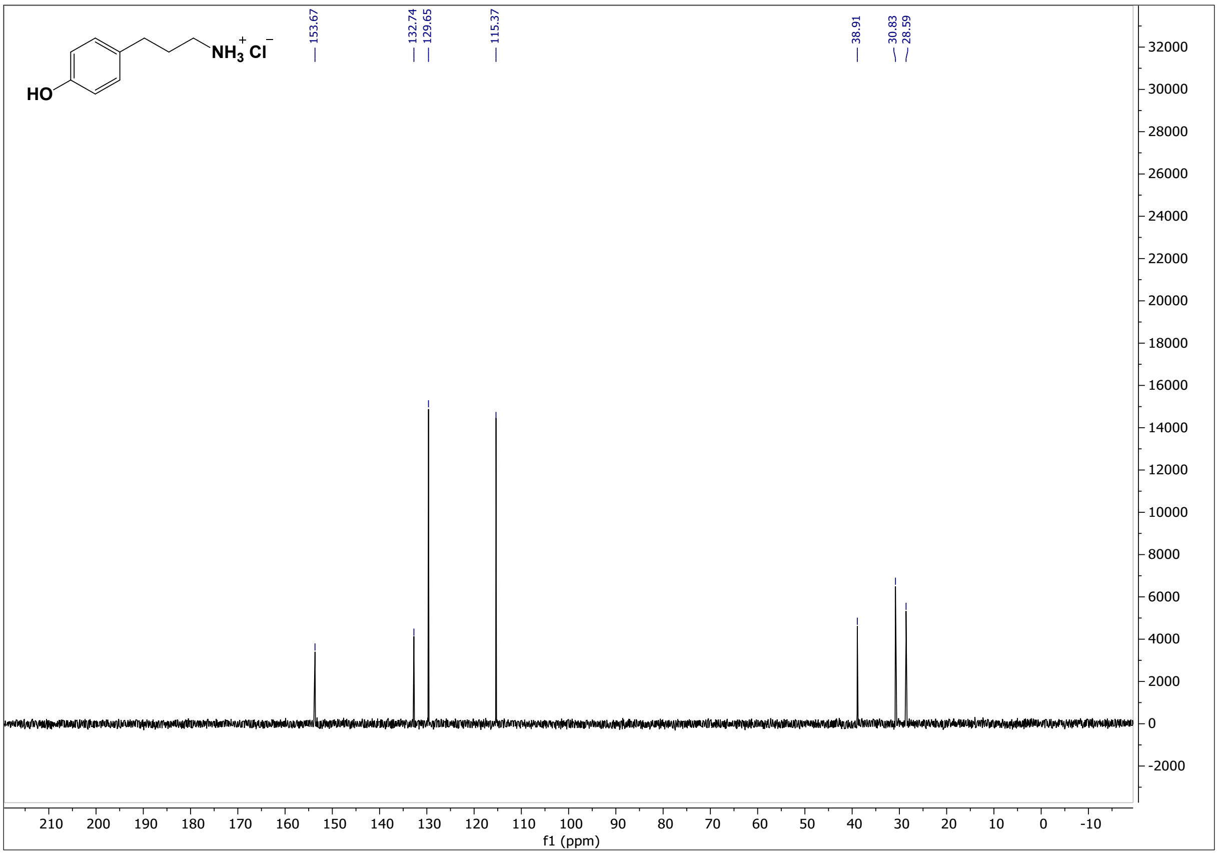

Figure S7. ^{13}C NMR spectrum of **1H amine** (as the HCl ammonium salt)

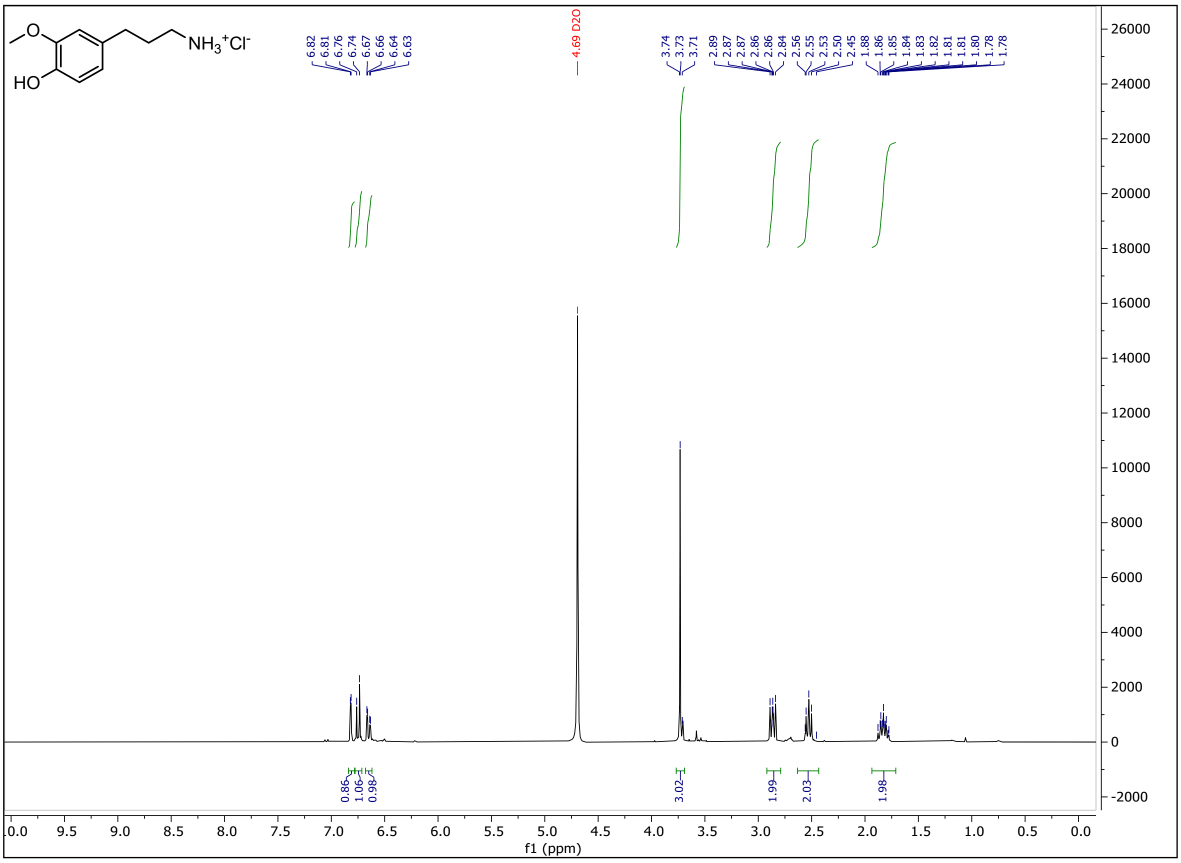

Figure S8. ^{1}H NMR spectrum of **1G amine** (as the HCl ammonium salt)

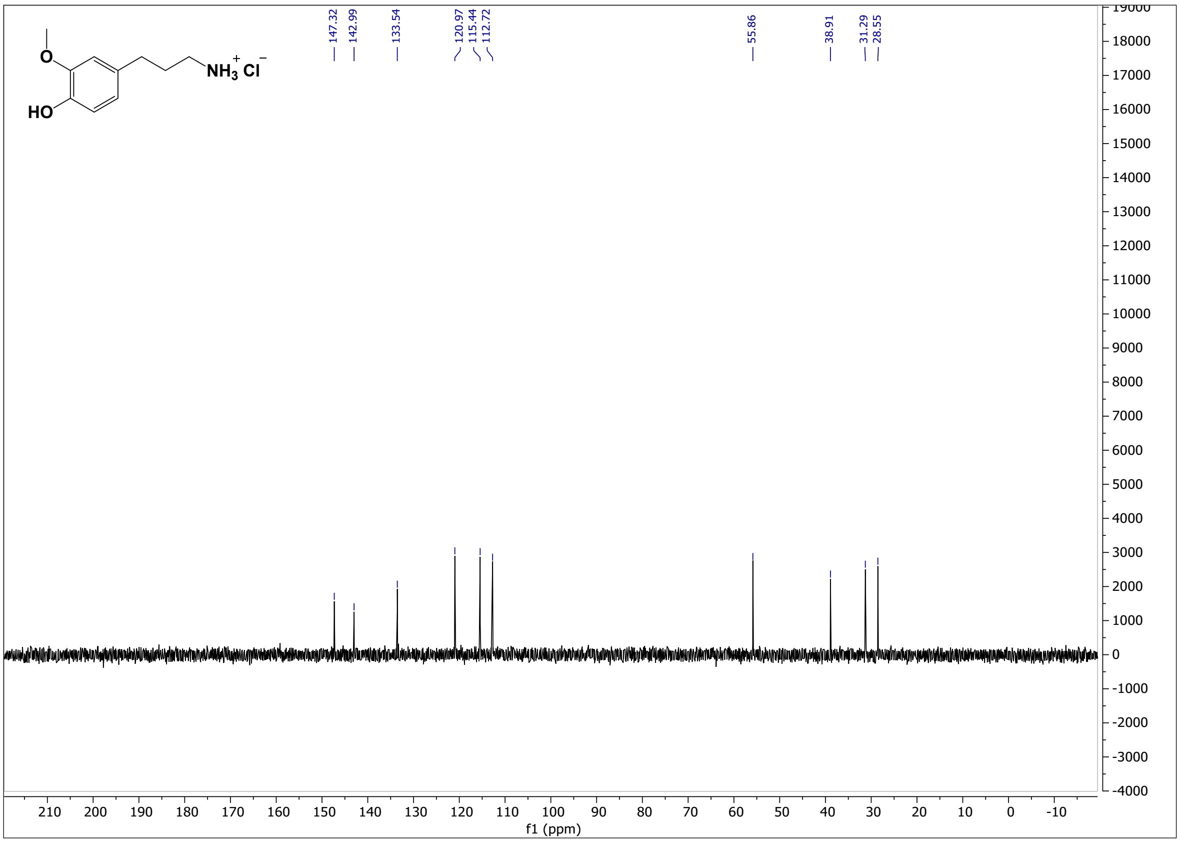

Figure S9. ^{13}C NMR spectrum of **1G amine** (as the HCl ammonium salt)

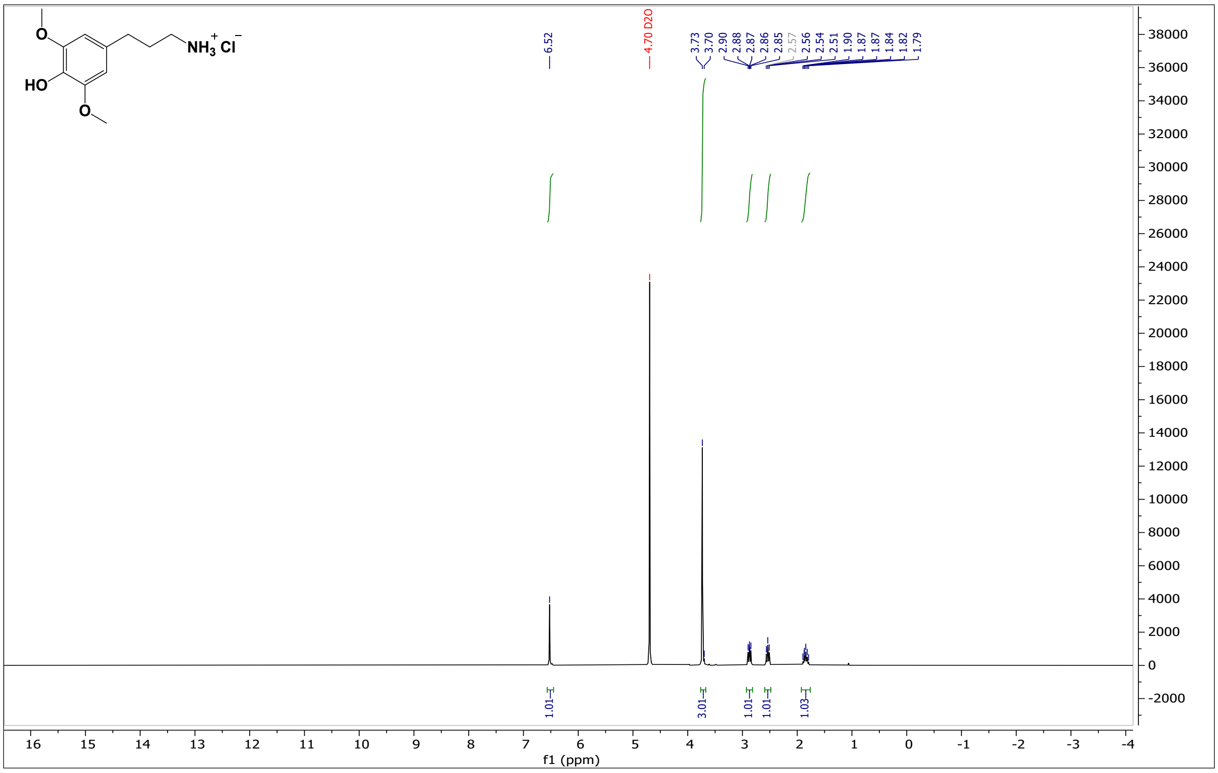

Figure S10. ^{1}H NMR spectrum of **1S amine** (as the HCl ammonium salt)

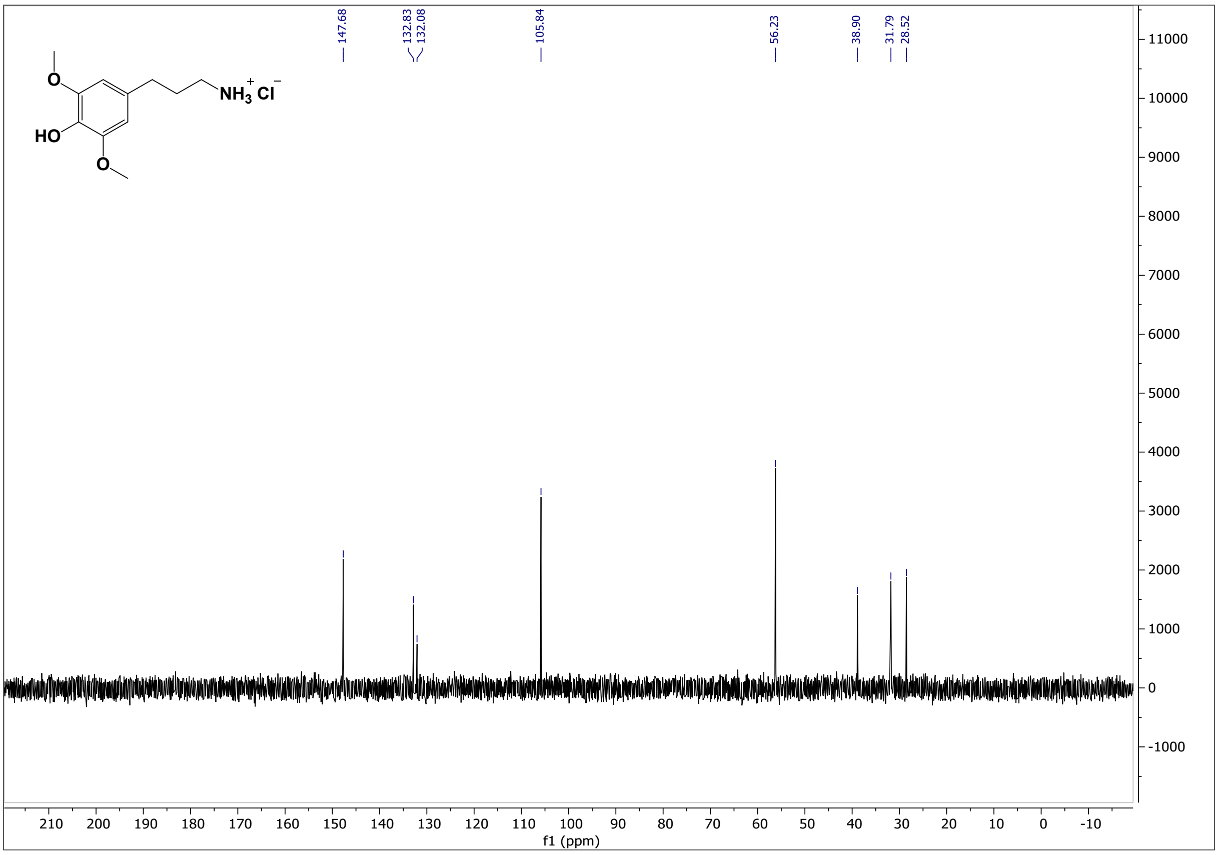

Figure S11. ^{13}C NMR spectrum of **1S amine** (as the HCl ammonium salt)

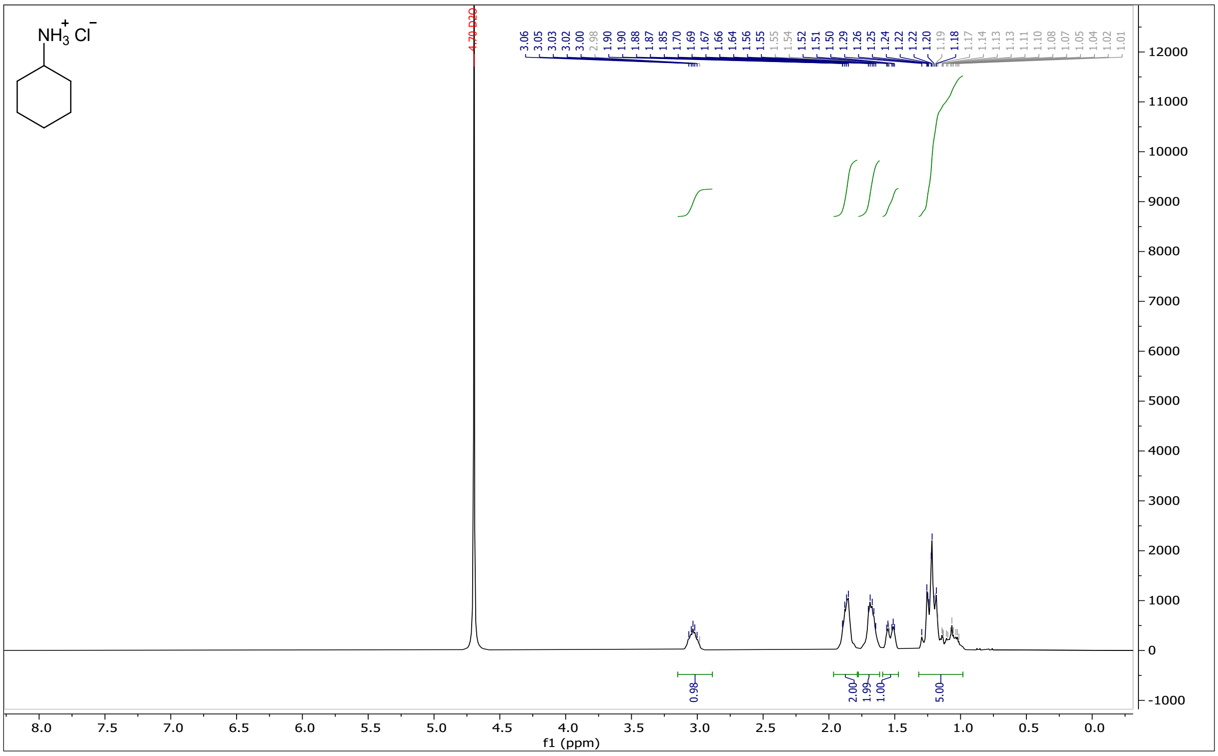

Figure S12. ^{1}H NMR spectrum of **1a** (as the HCl ammonium salt)

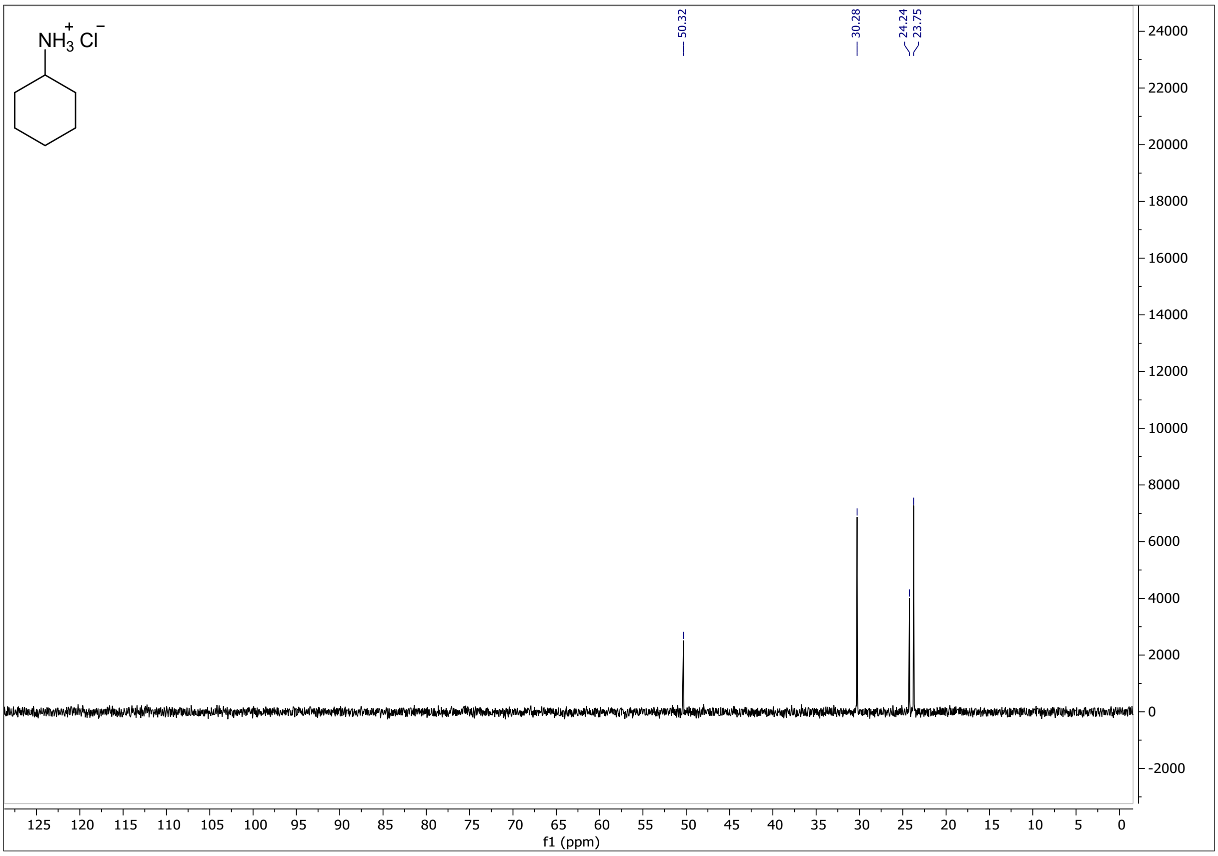

Figure S13. ^{13}C NMR spectrum of **1a** (as the HCl ammonium salt)

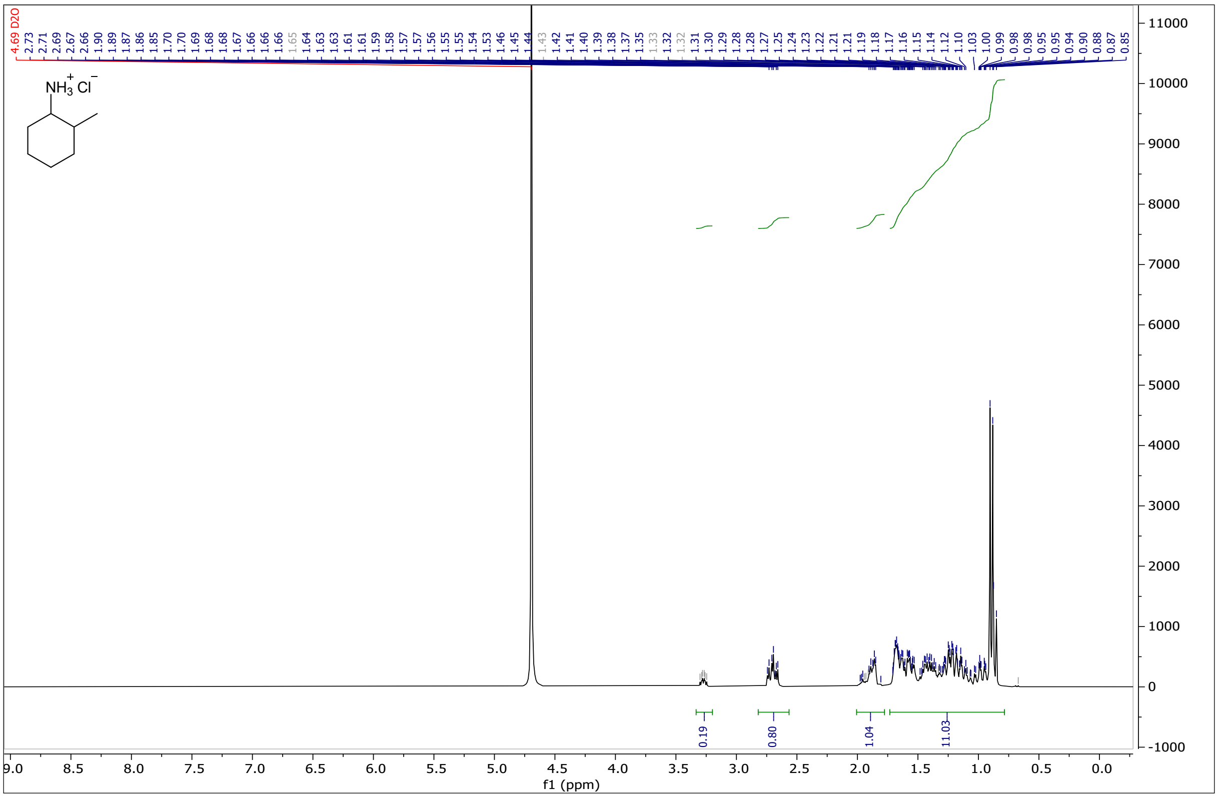

Figure S14. ^{1}H NMR spectrum of **2a** (as the HCl ammonium salt)

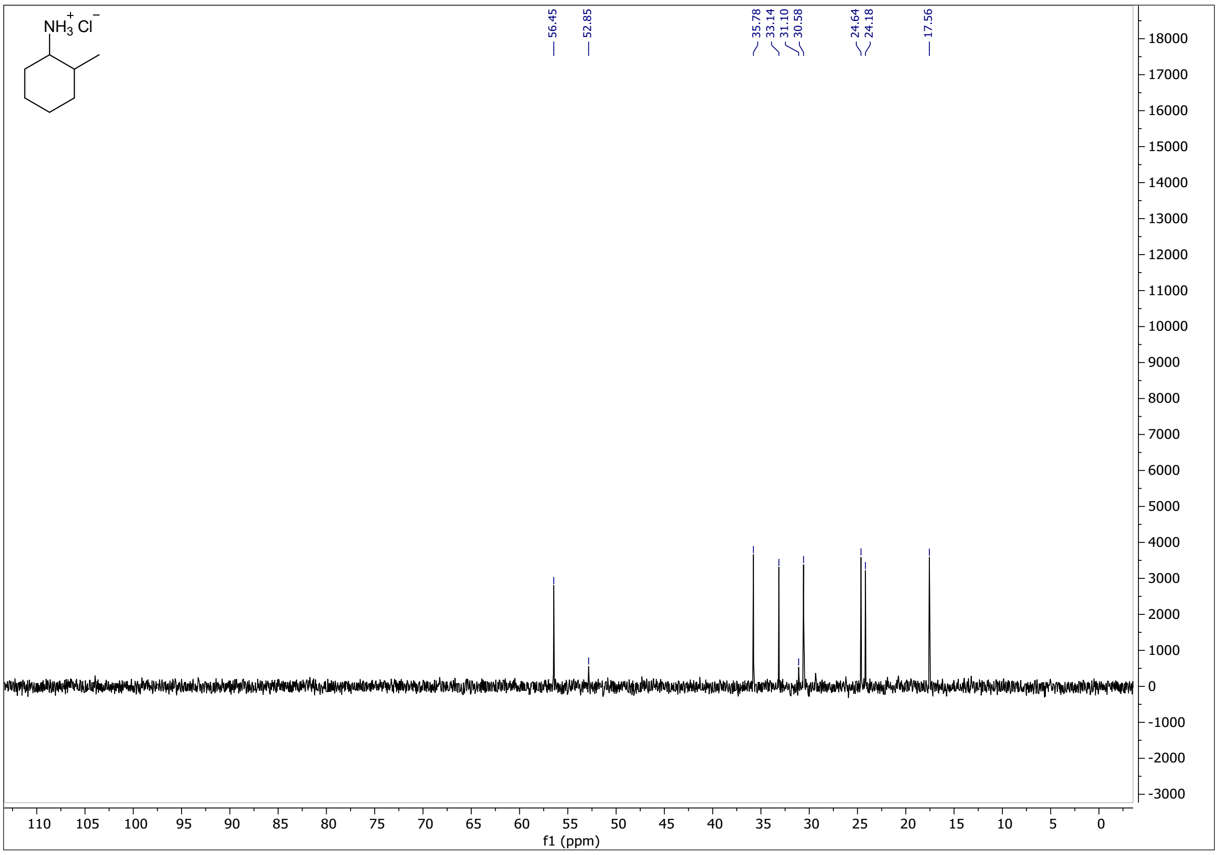

Figure S15. ^{13}C NMR spectrum of **2a** (as the HCl ammonium salt)

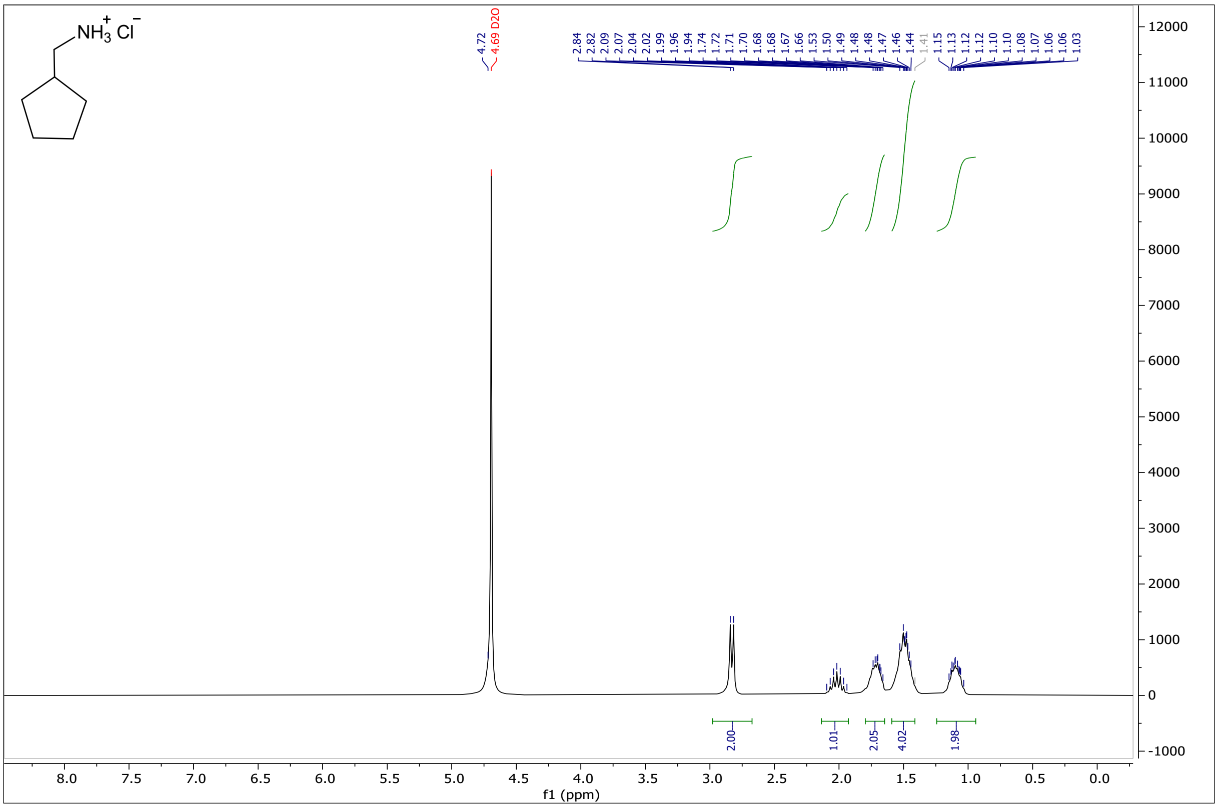

Figure S16. ^{1}H NMR spectrum of **3a** (as the HCl ammonium salt)

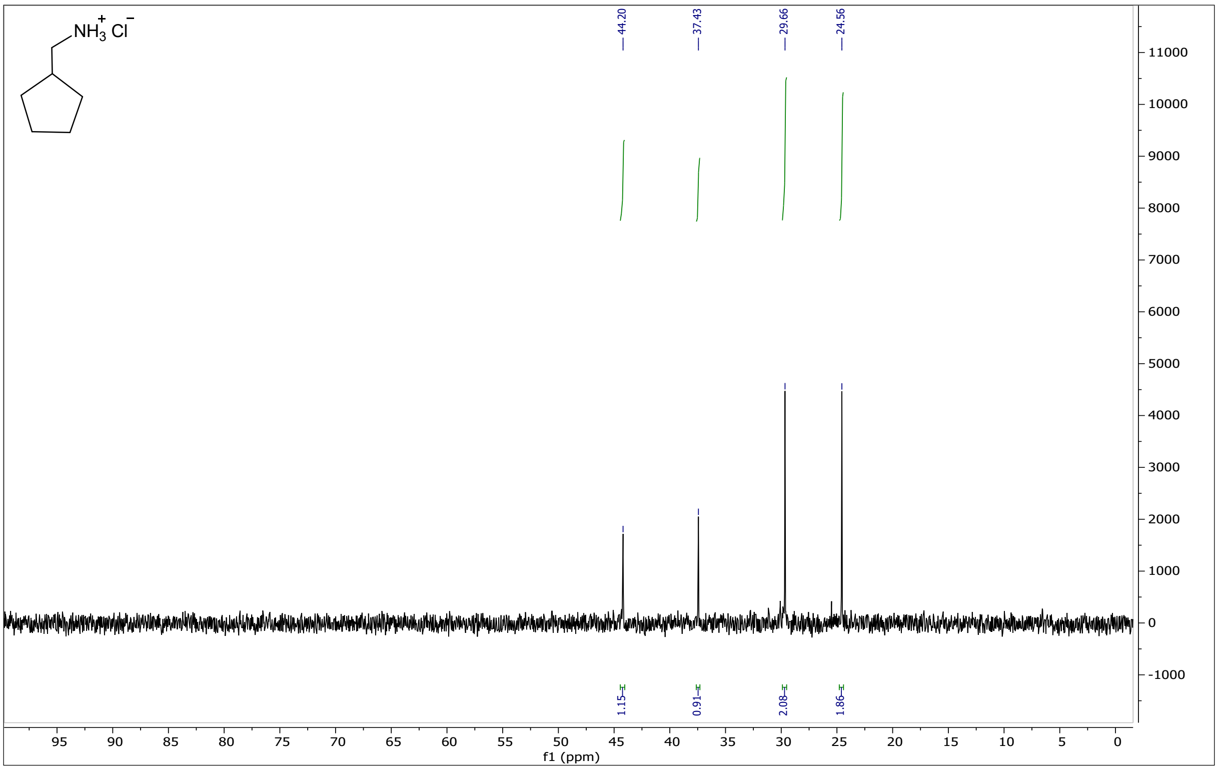

Figure S17. ^{13}C NMR spectrum of **3a** (as the HCl ammonium salt)

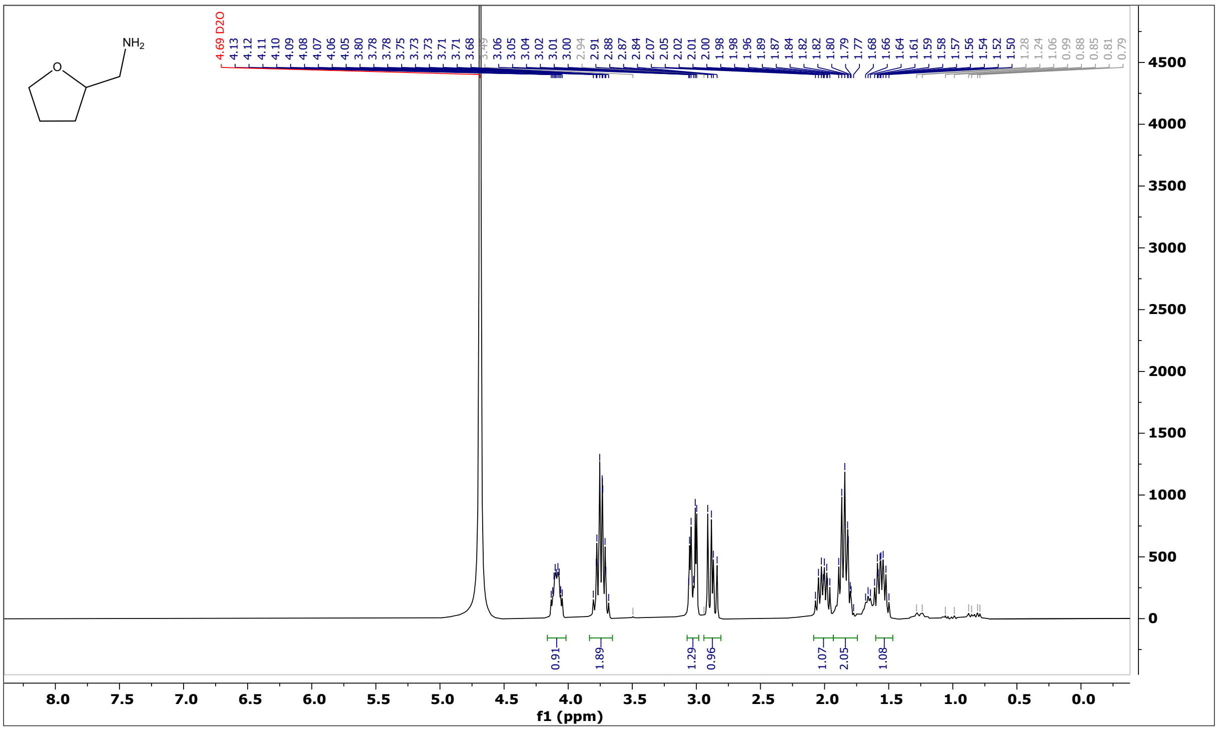

Figure S18. ^{1}H NMR spectrum of **4a**

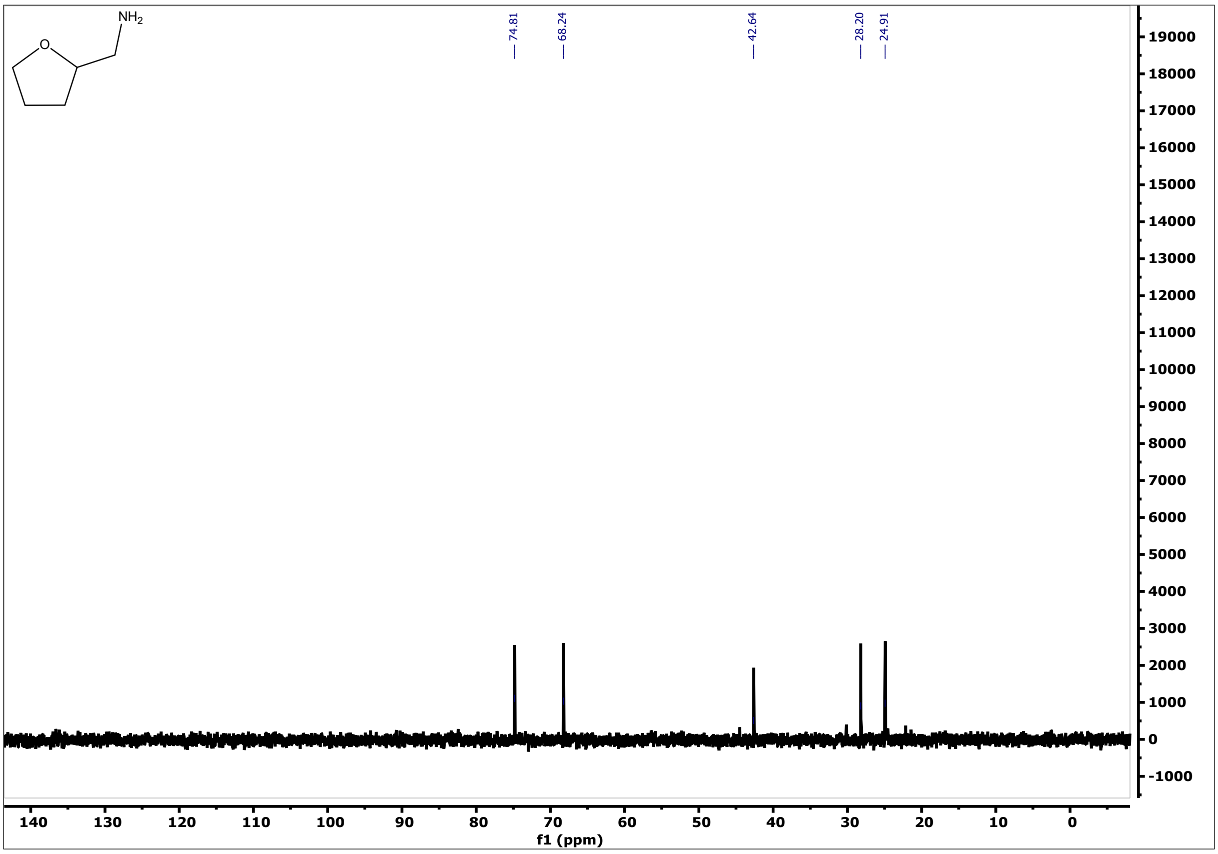

Figure S19. ^{13}C NMR spectrum of **4a**

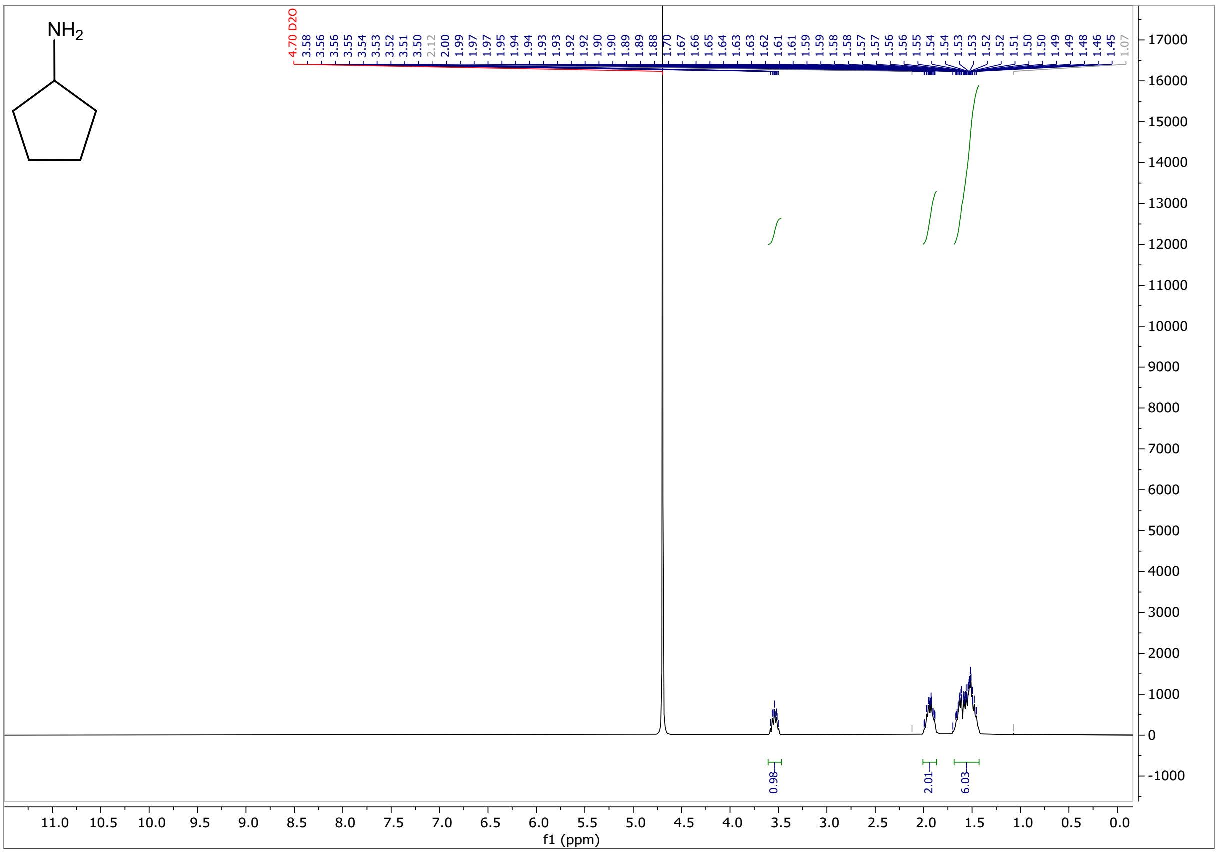

Figure S20. ^{1}H NMR spectrum of **5a**

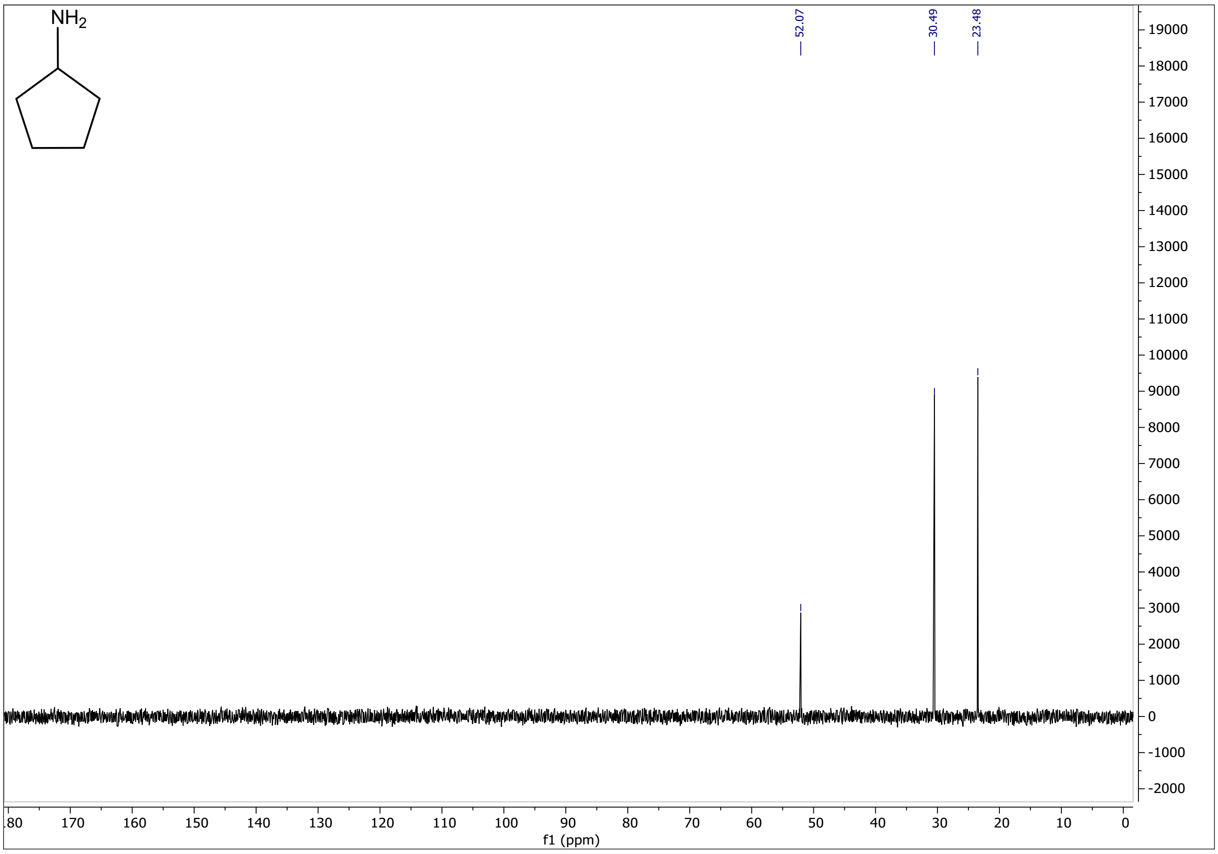

Figure S21. ^{13}C NMR spectrum of **5a**

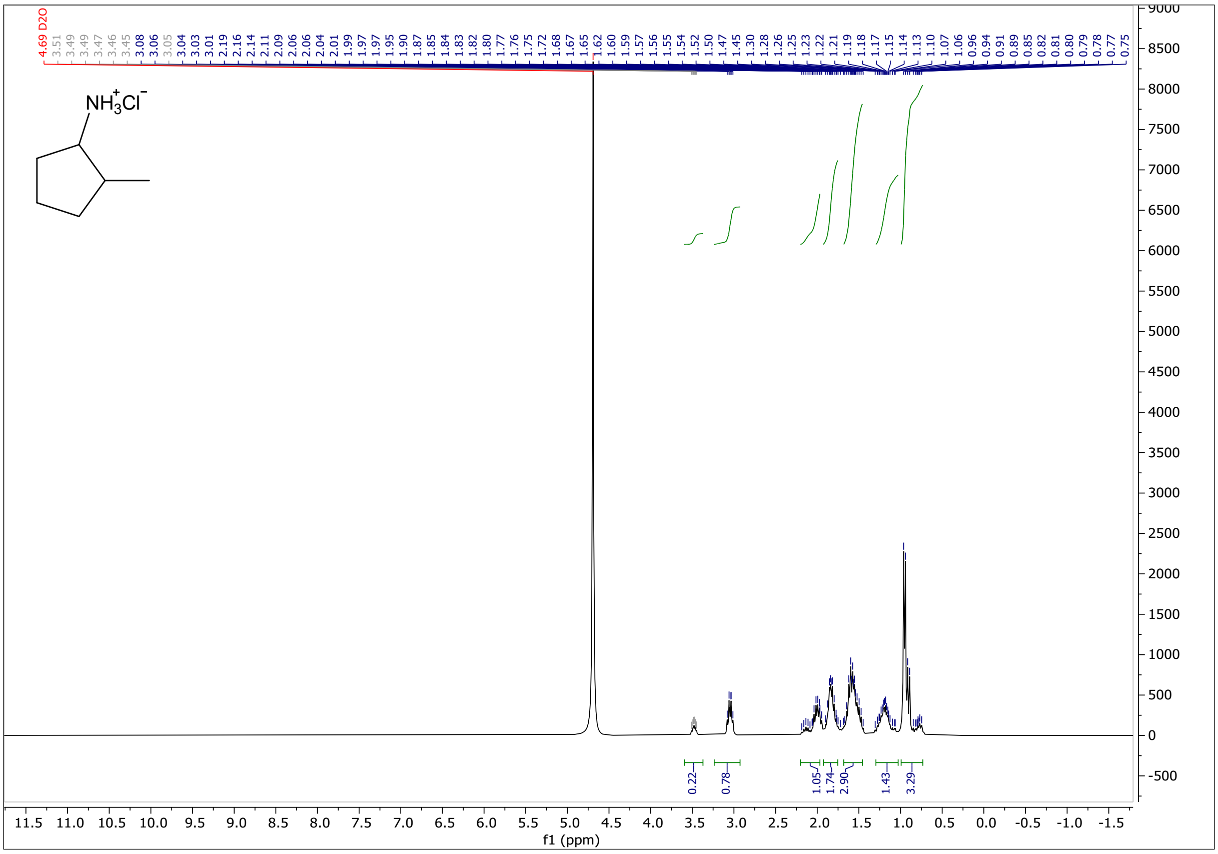

Figure S22. ^{1}H NMR spectrum of **6a** (as the HCl ammonium salt)

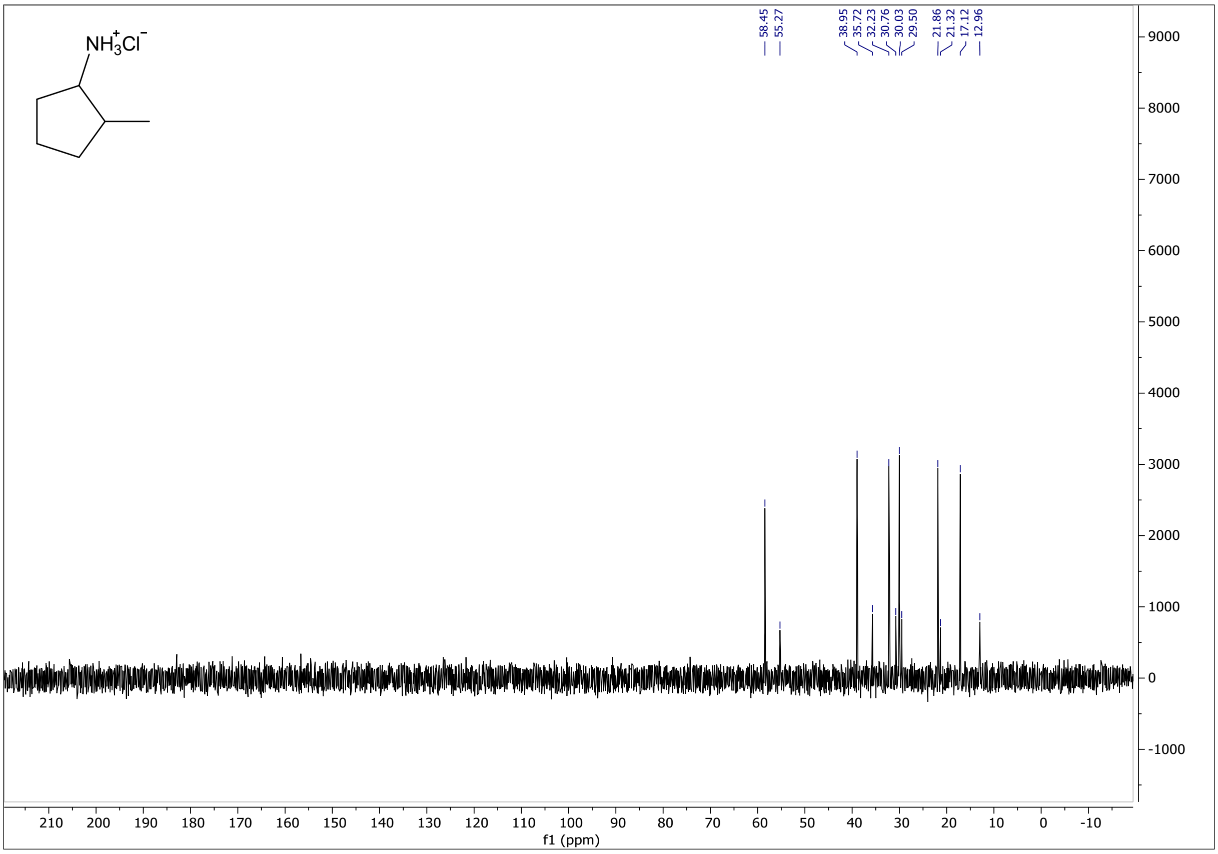

Figure S23. ^{13}C NMR spectrum of **6a** (as the HCl ammonium salt)

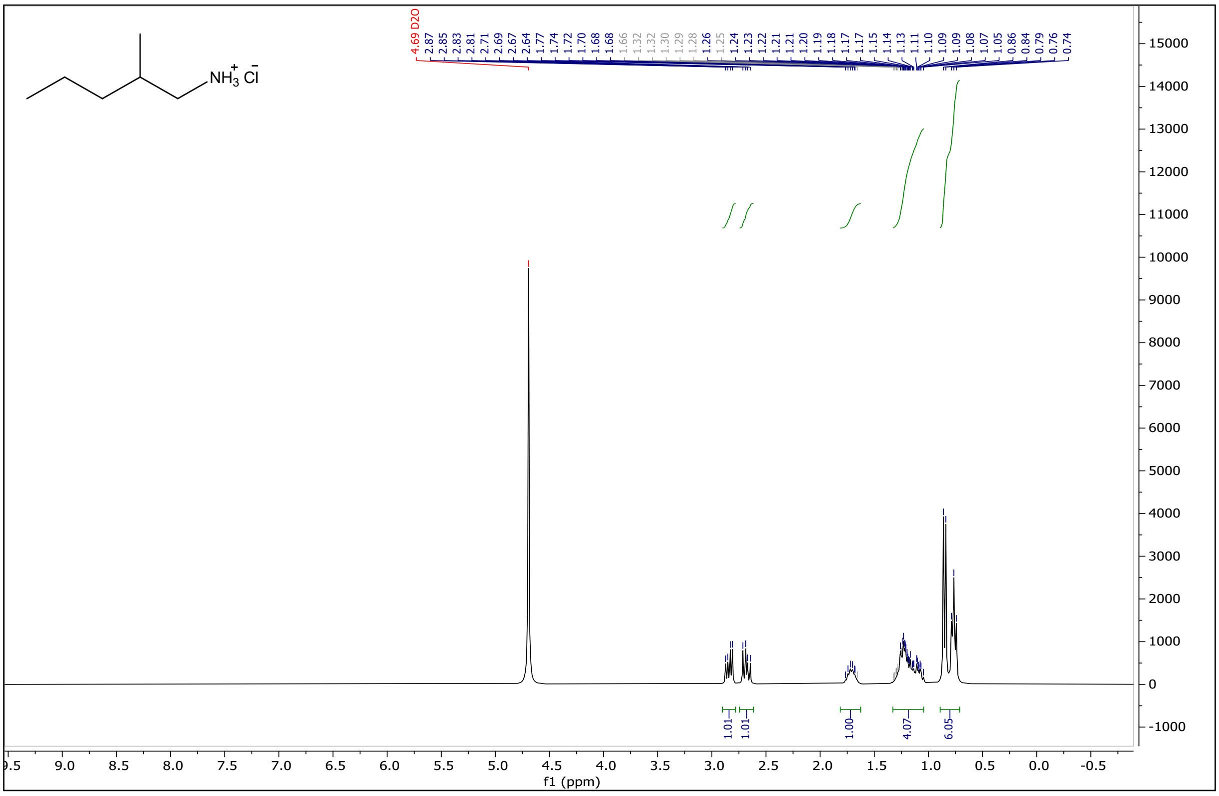

Figure S24. ^{1}H-NMR spectrum of **7a** (as the HCl ammonium salt)

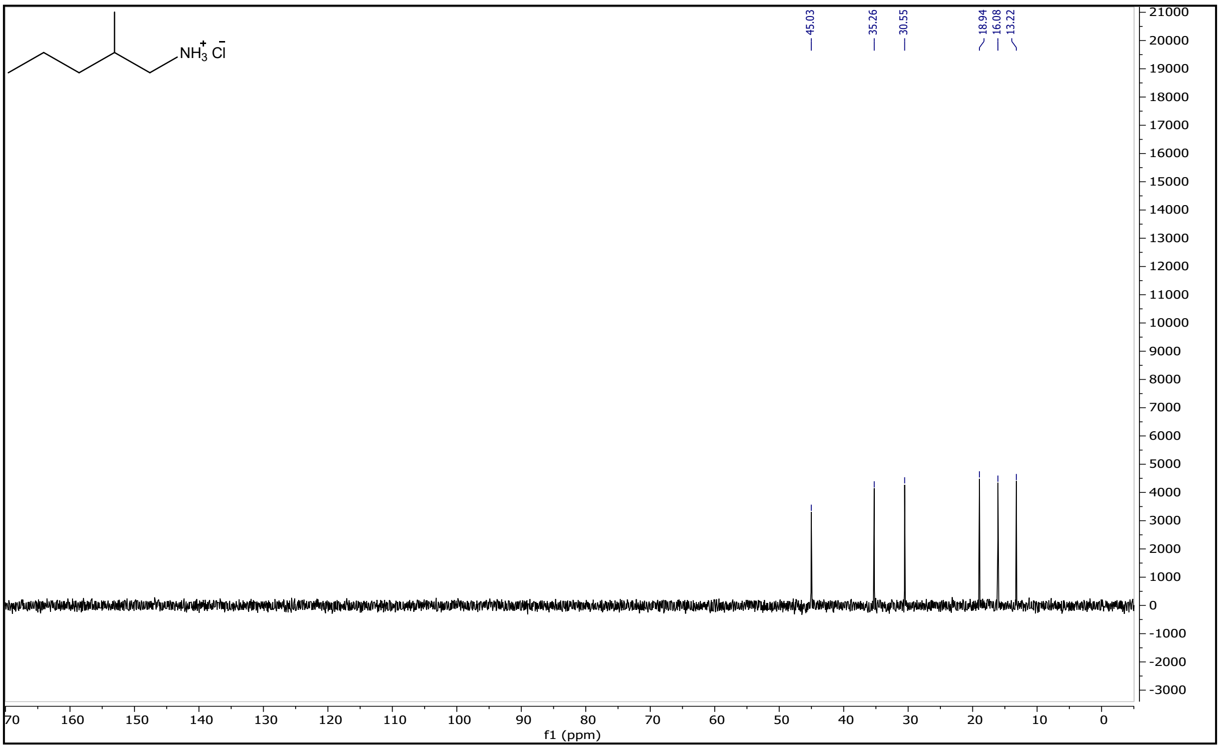

Figure S25. ^{13}C NMR spectrum of **7a** (as the HCl ammonium salt)

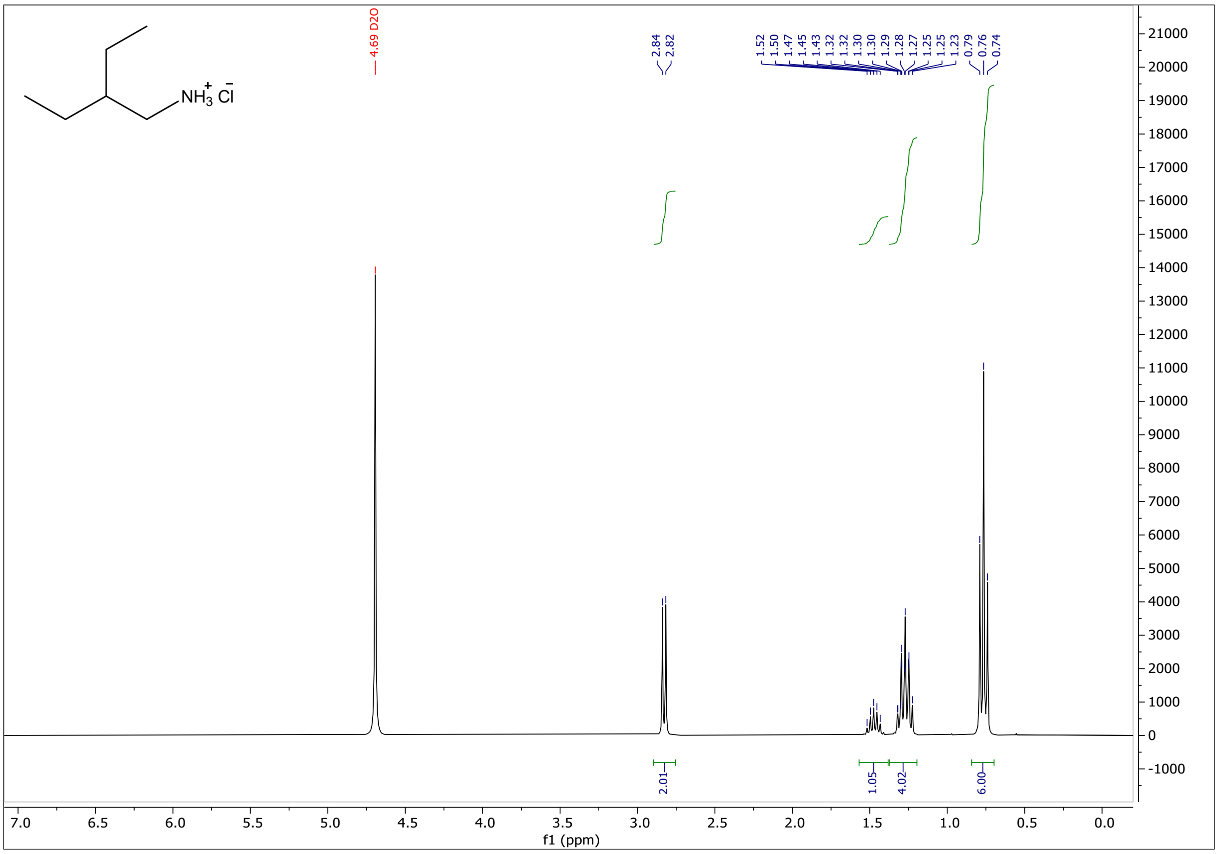

Figure S26. ^{1}H NMR spectrum of **8a** (as the HCl ammonium salt)

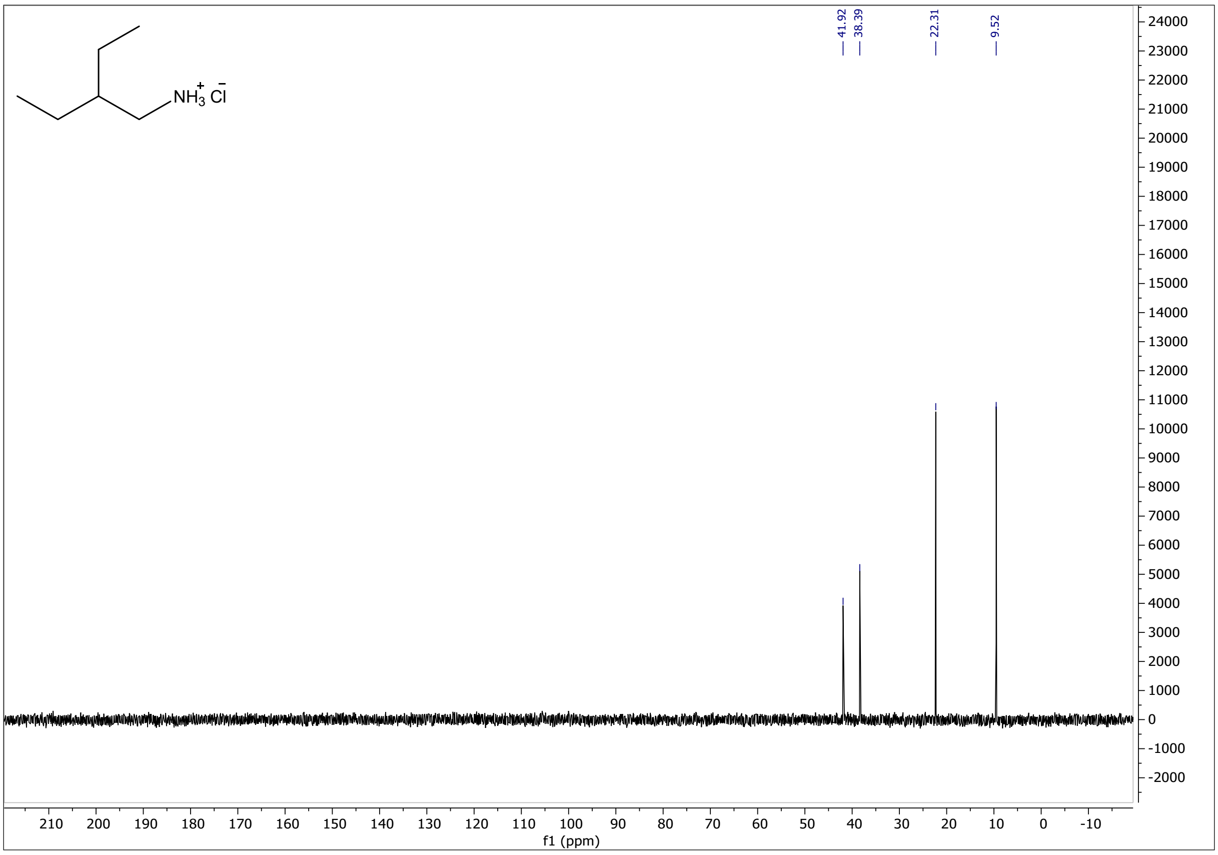

Figure S27. ^{13}C NMR spectrum of **8a** (as the HCl ammonium salt)

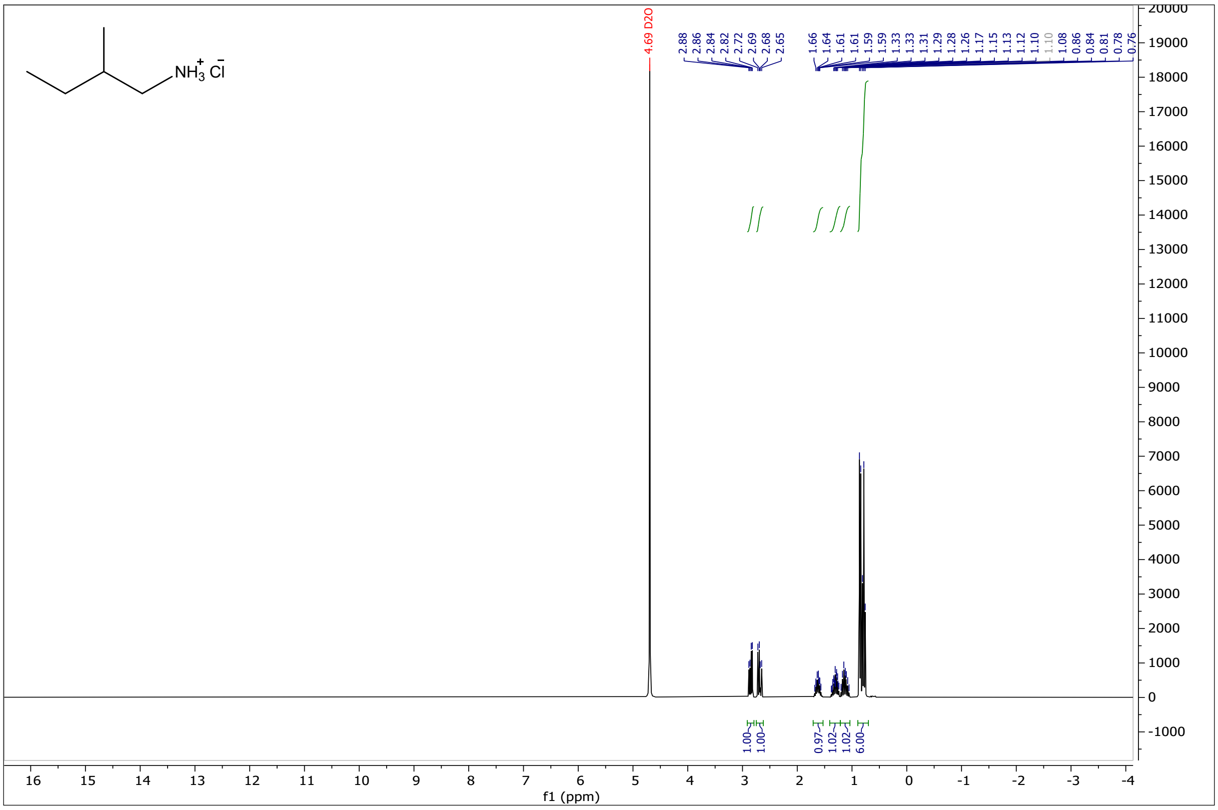

Figure S28. ^{1}H NMR spectrum of **9a** (as the HCl ammonium salt)

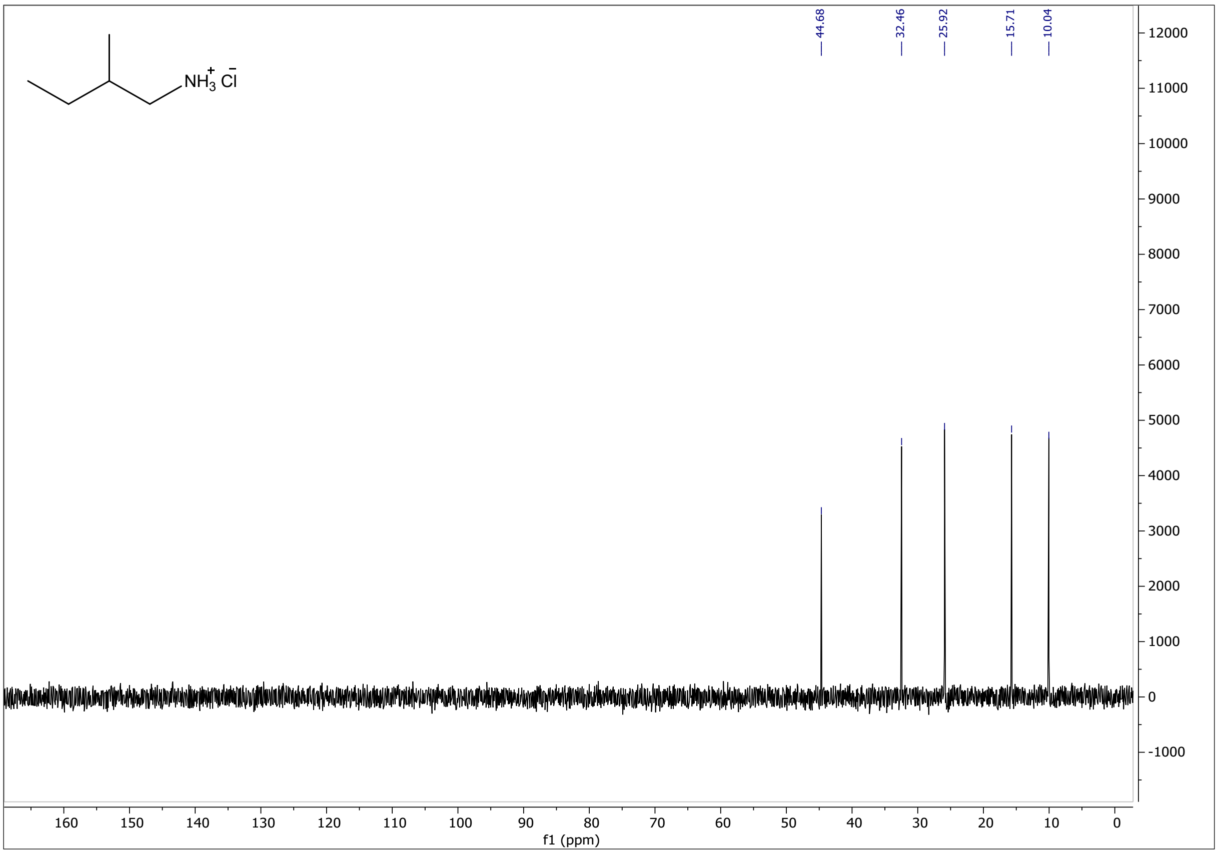

Figure S29. ^{13}C NMR spectrum of **9a** (as the HCl ammonium salt)

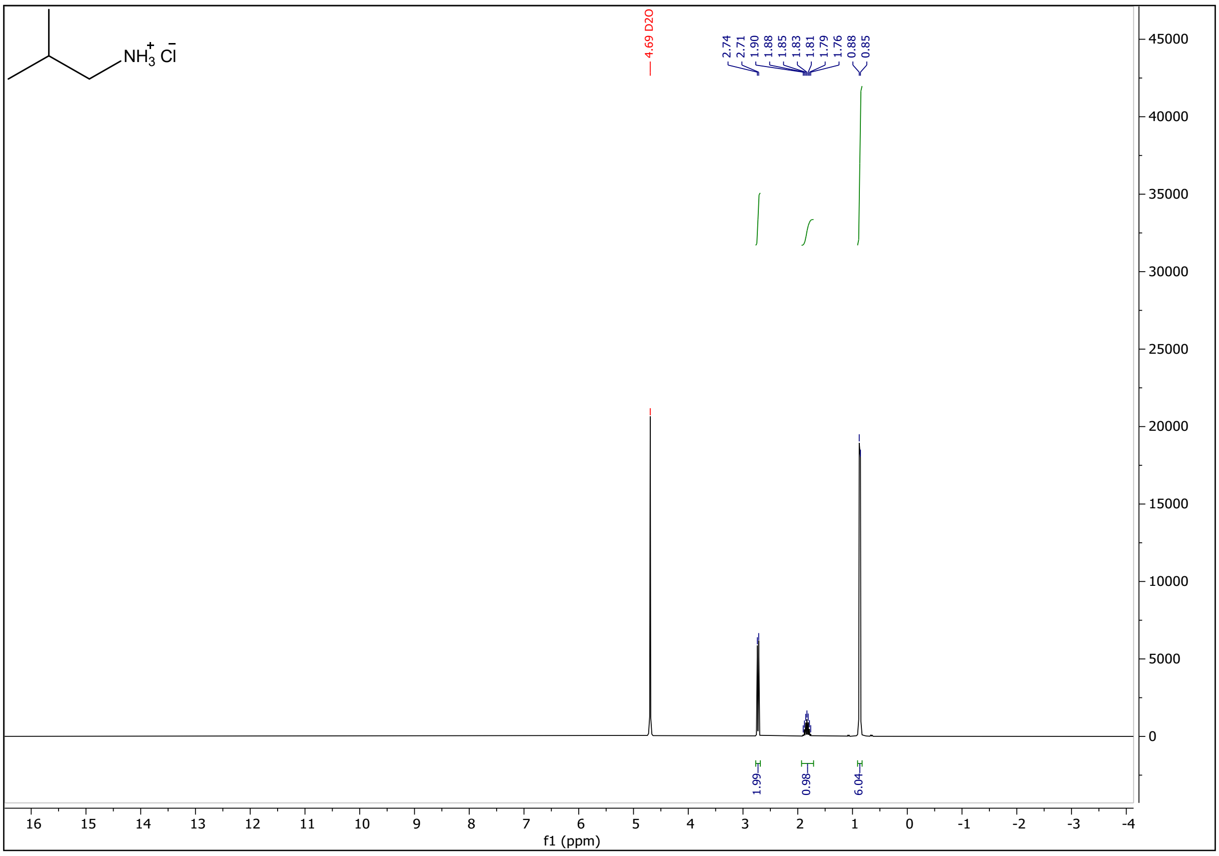

Figure S30. ^{1}H NMR spectrum of **10a** (as the HCl ammonium salt)

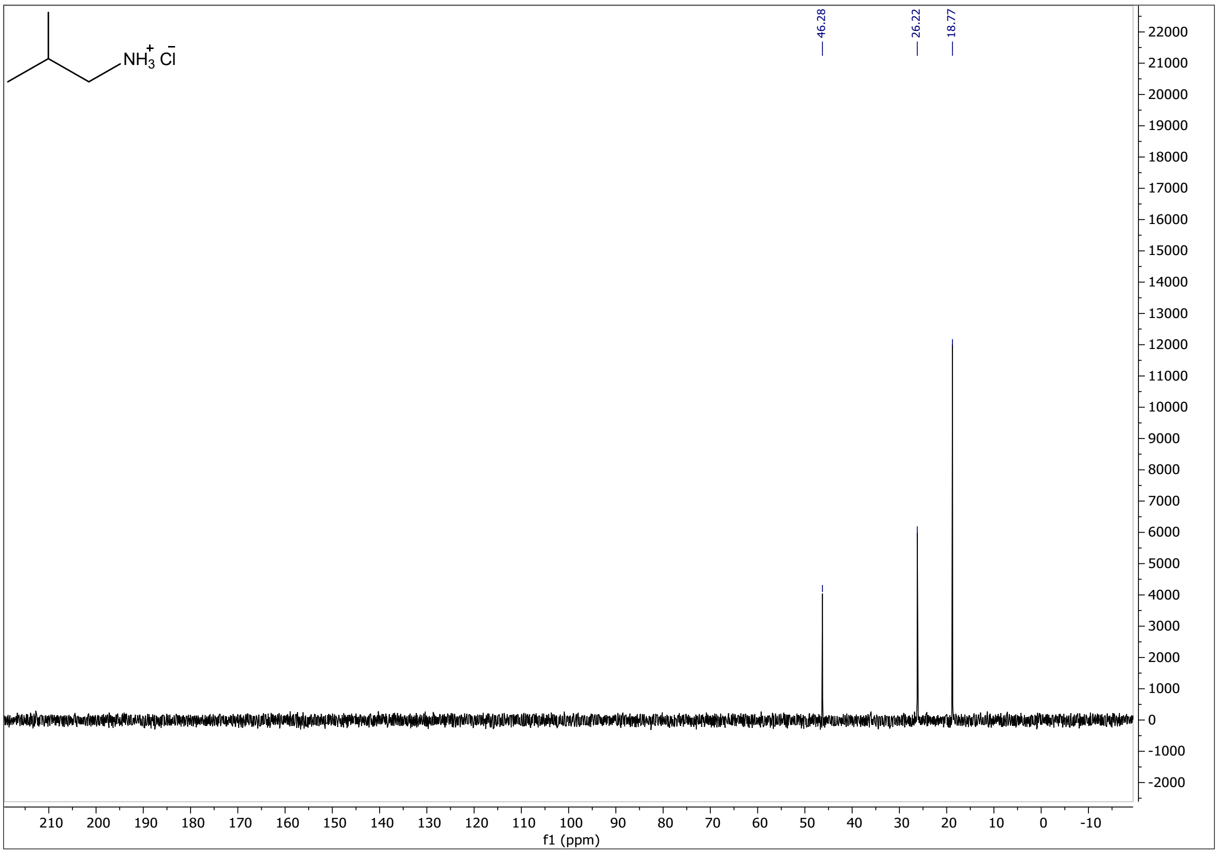

Figure S31. ^{13}C NMR spectrum of **10a** (as the HCl ammonium salt)

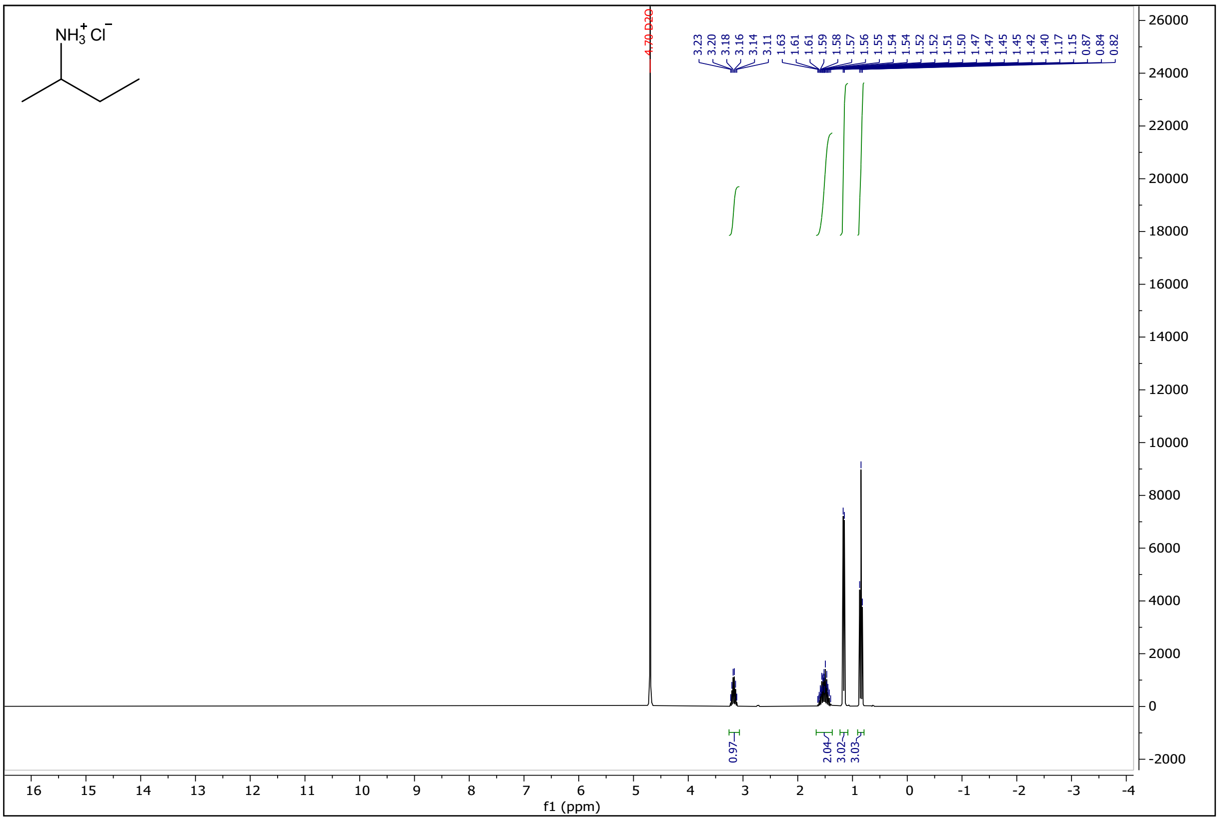

Figure S32. ^{1}H NMR spectrum of **11a** (as the HCl ammonium salt)

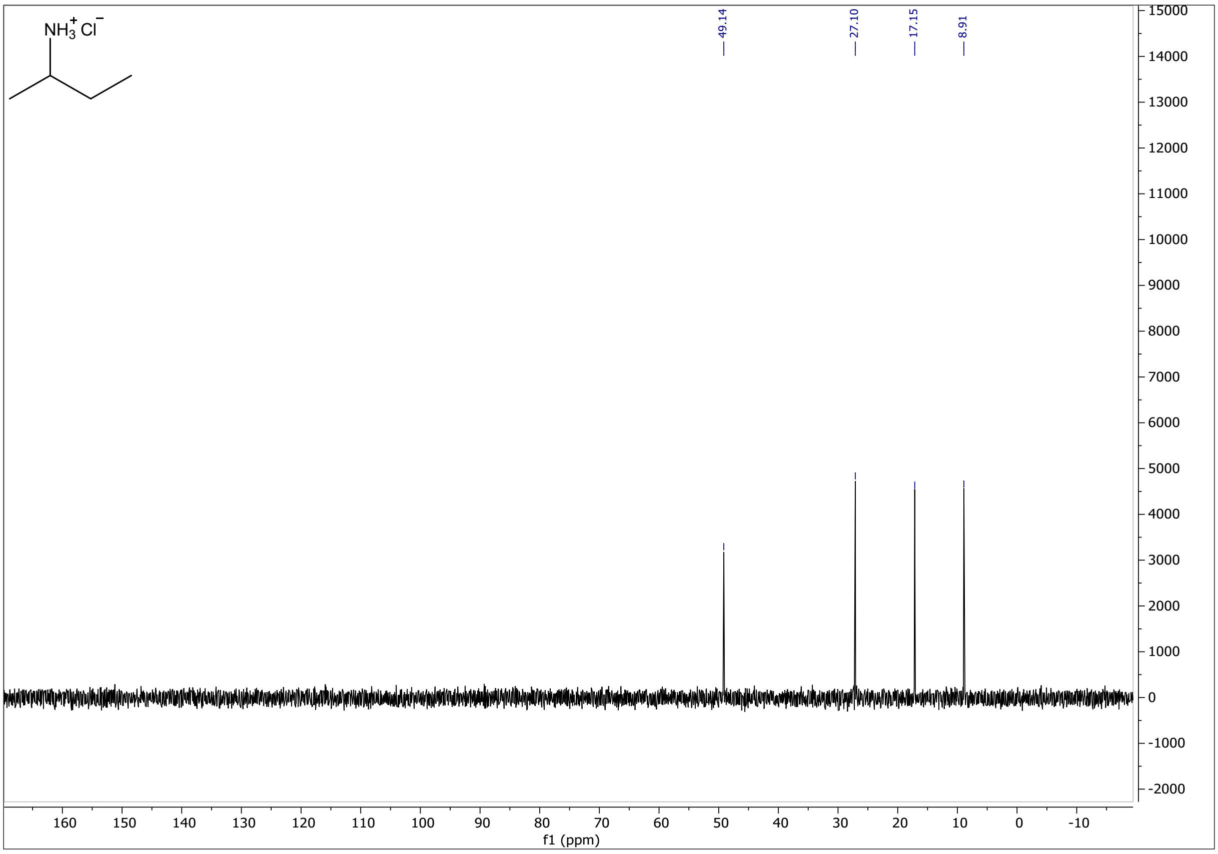

Figure S33. ^{13}C NMR spectrum of **11a** (as the HCl ammonium salt)

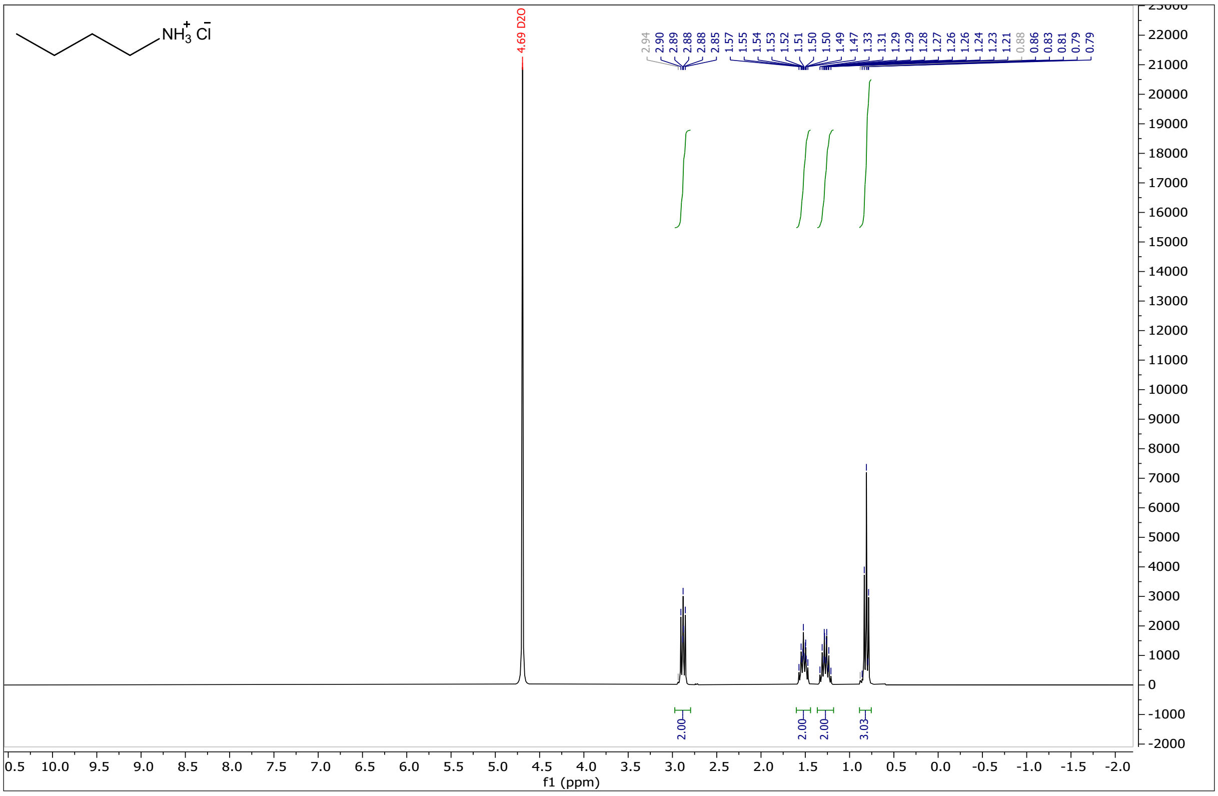

Figure S34. ^{1}H NMR spectrum of **12a** (as the HCl ammonium salt)

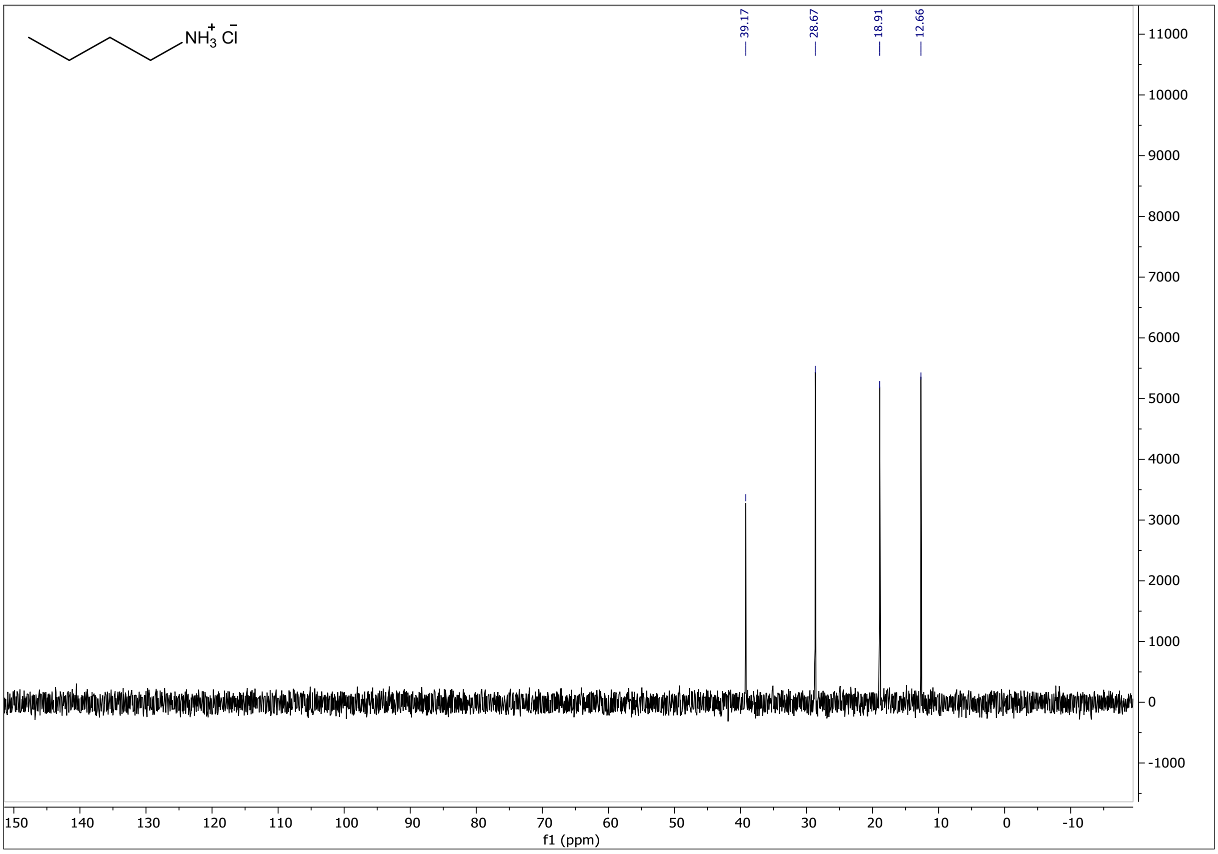

Figure S35. ^{13}C NMR spectrum of **12a** (as the HCl ammonium salt)

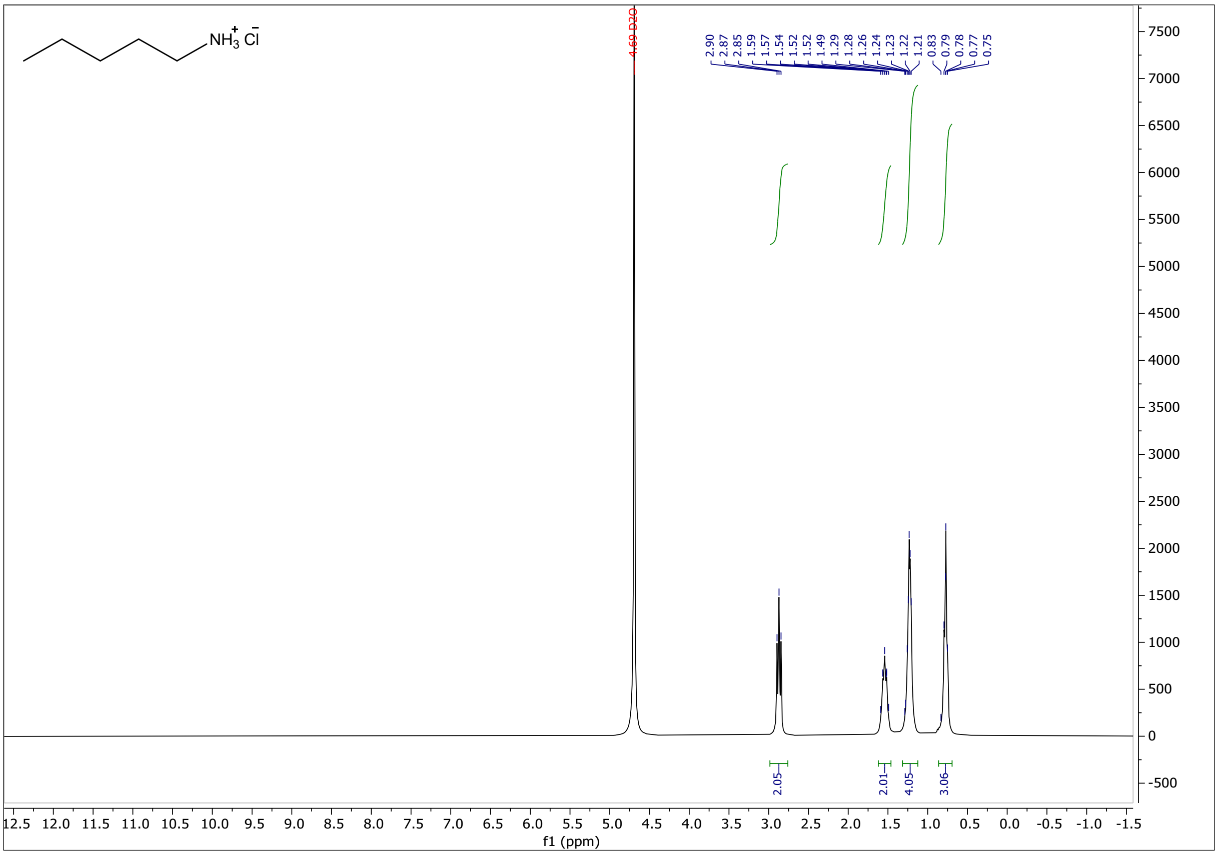

Figure S36. ^{1}H NMR spectrum of **13a** (as the HCl ammonium salt)

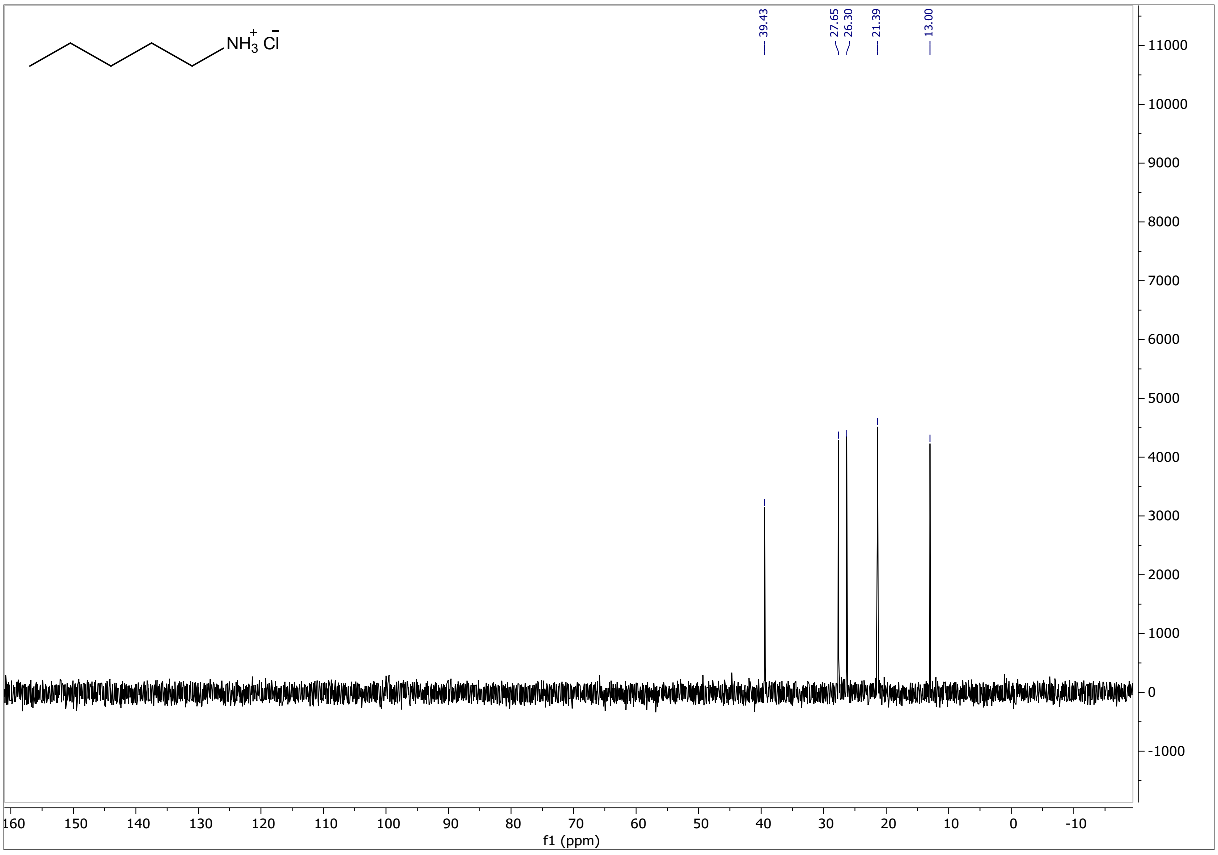

Figure S37. ^{13}C NMR spectrum of **13a** (as the HCl ammonium salt)

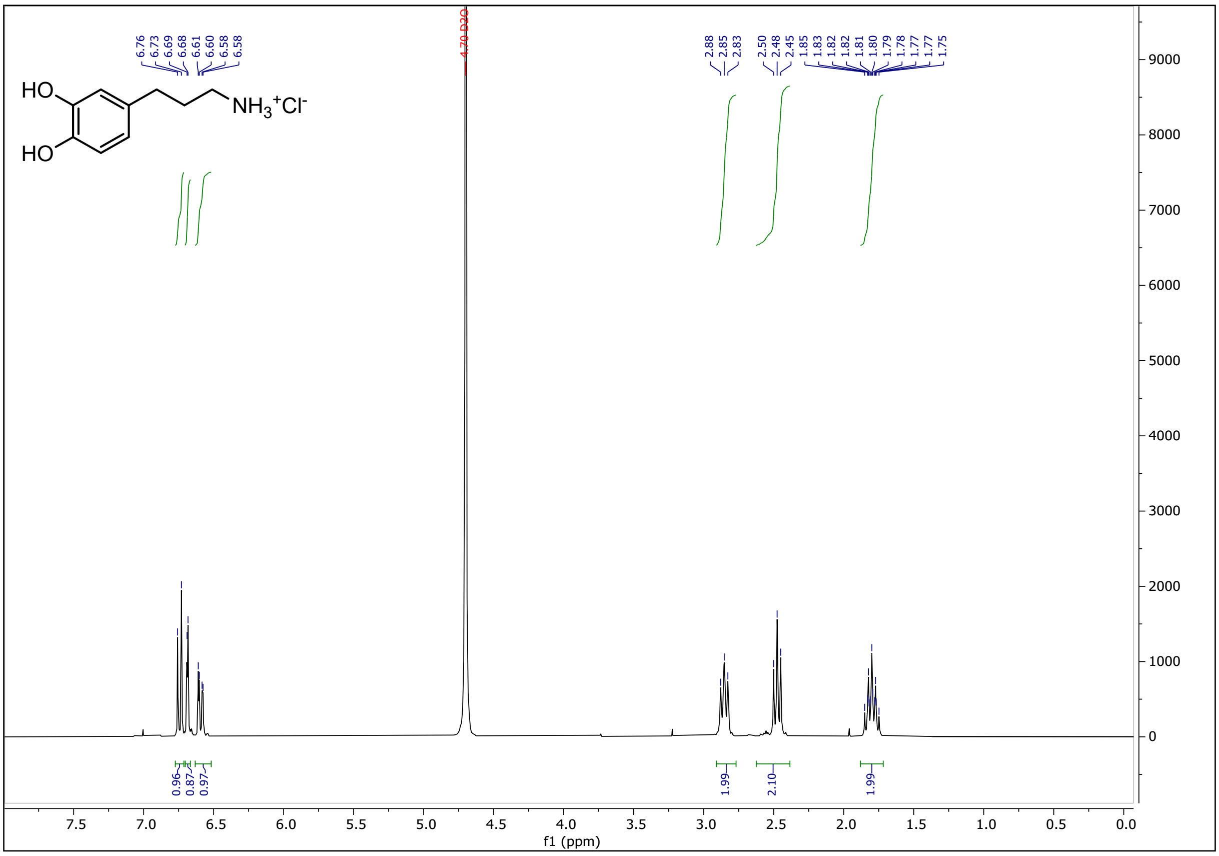

Figure S38. ^{1}H NMR spectrum of **1B** (as the HCl ammonium salt)

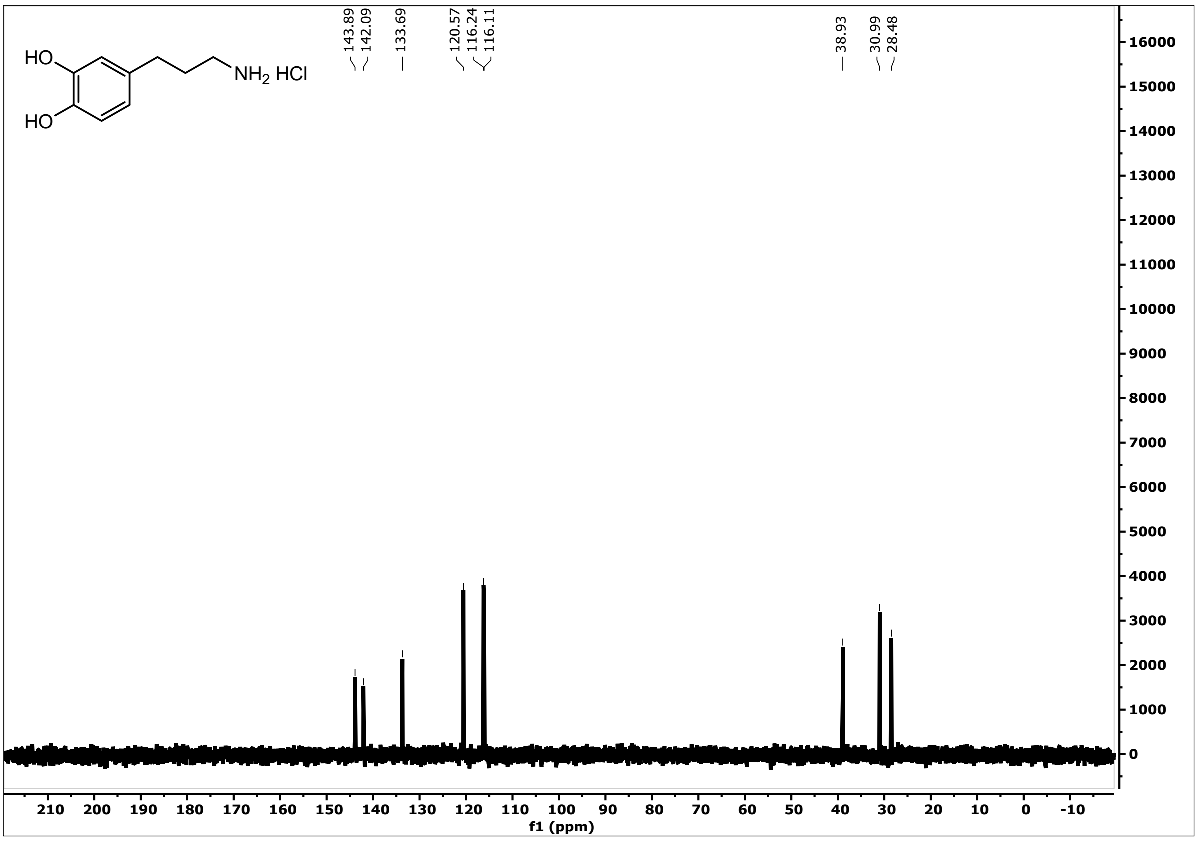

Figure S39. ^{13}C NMR spectrum of **1B** (as the HCl ammonium salt)

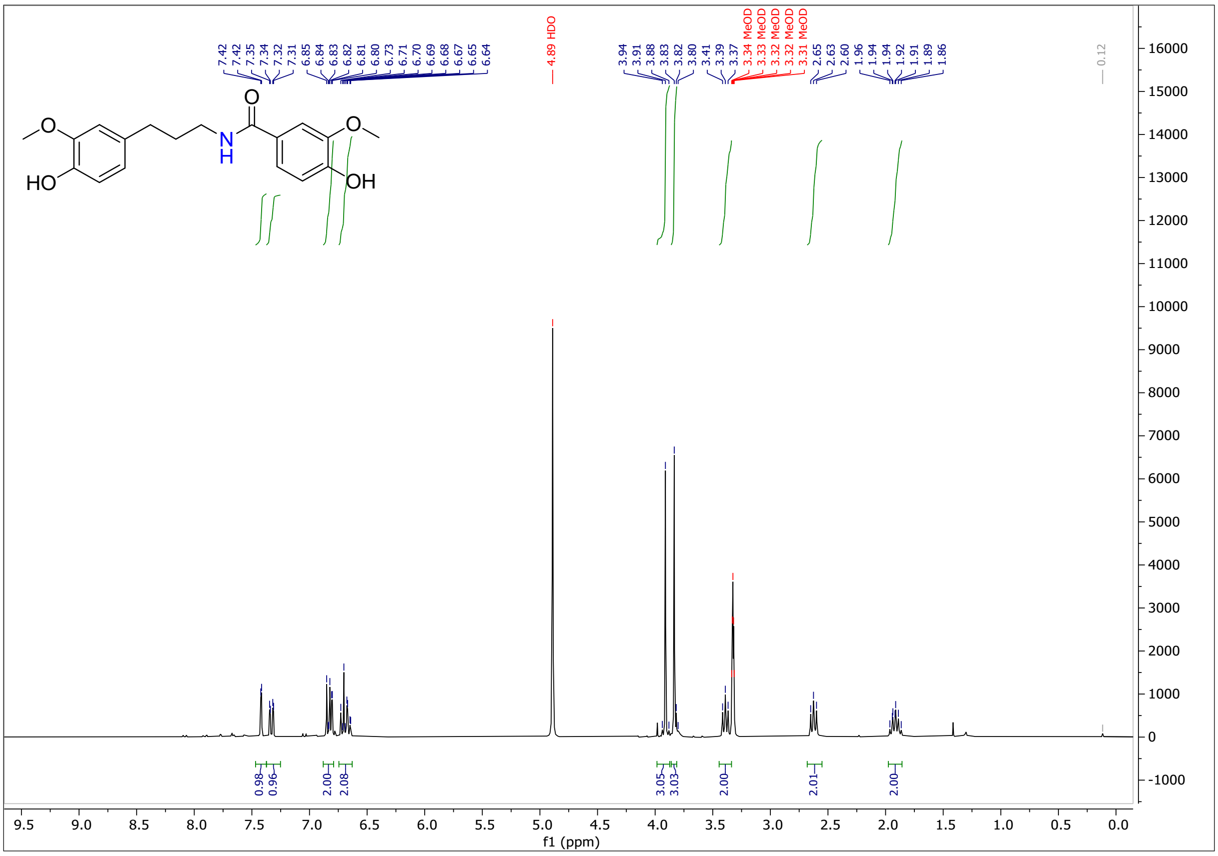

Figure S40. ^{1}H NMR spectrum of **2B**

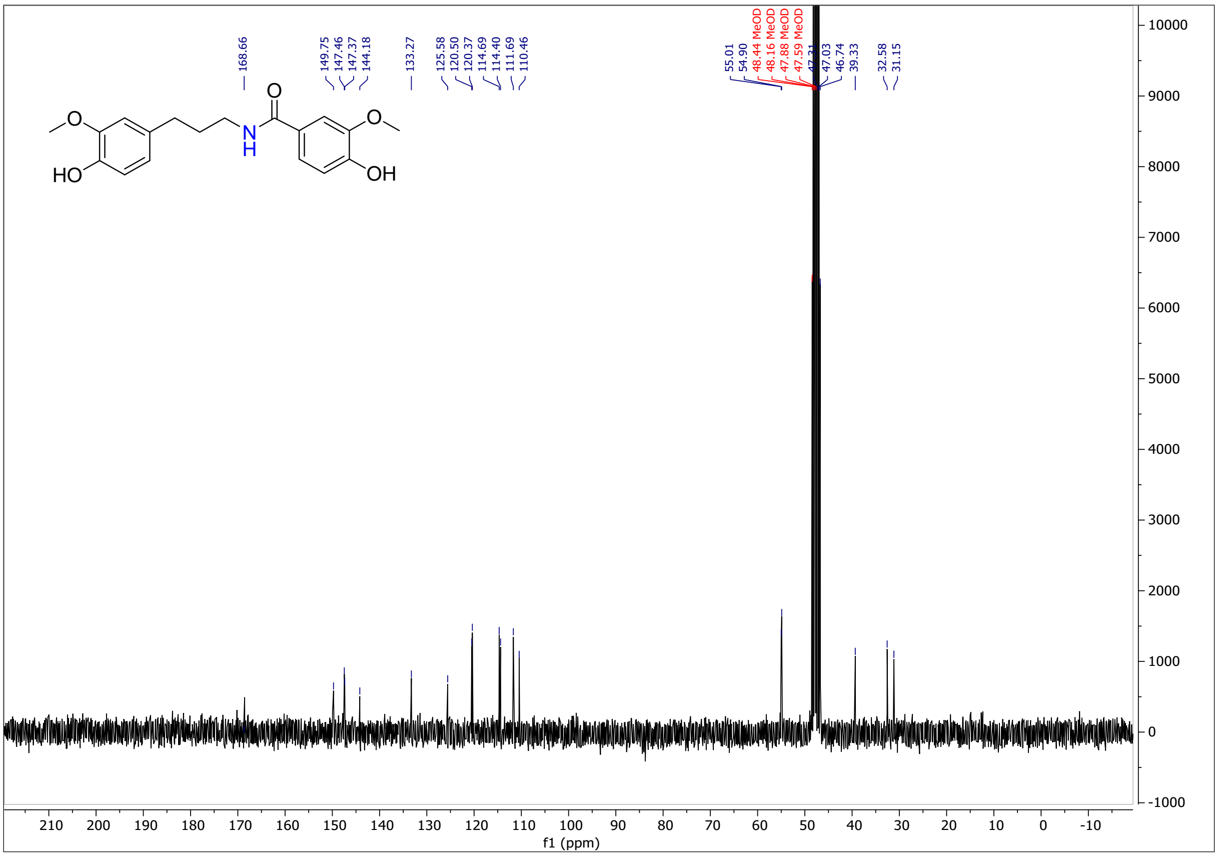

Figure S41. ^{13}C NMR spectrum of **2B**

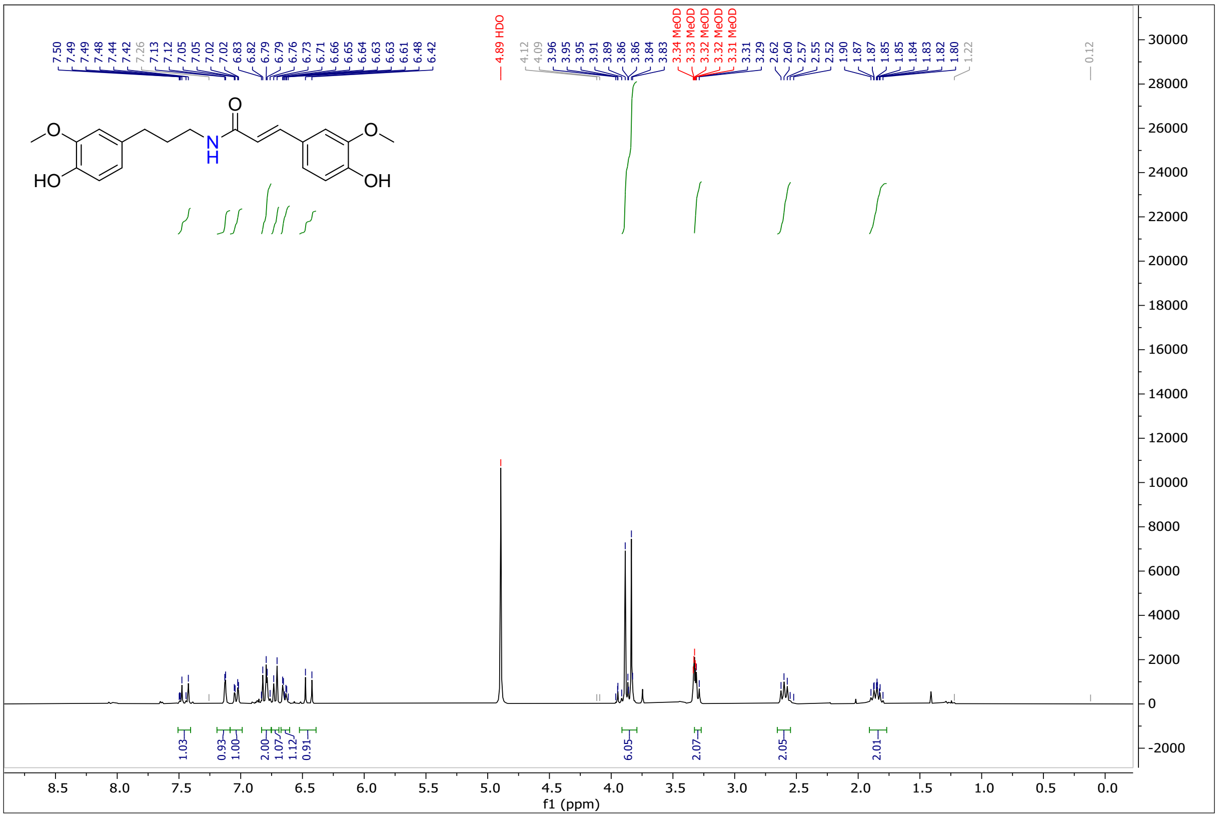

Figure S42. ^{1}H NMR spectrum of **3B**

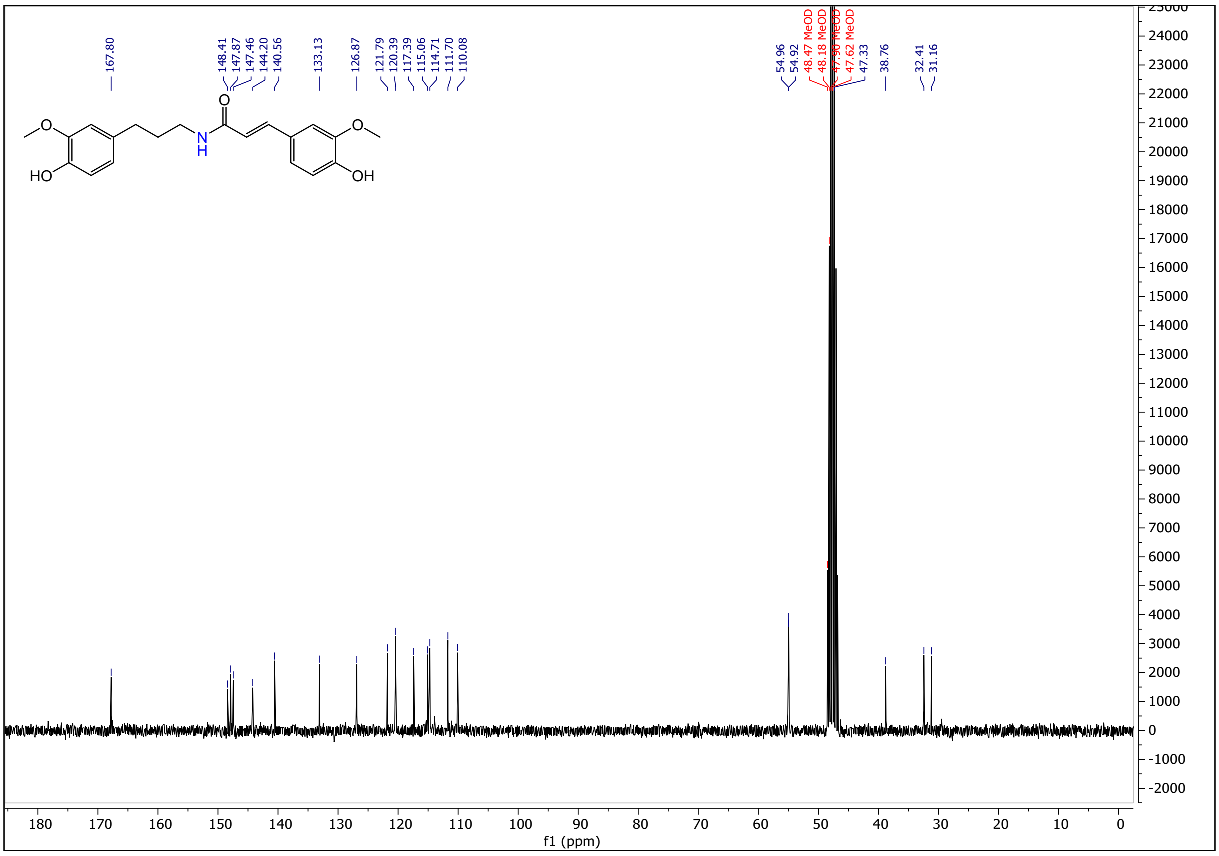

Figure S43. ^{13}C NMR spectrum of **3B**

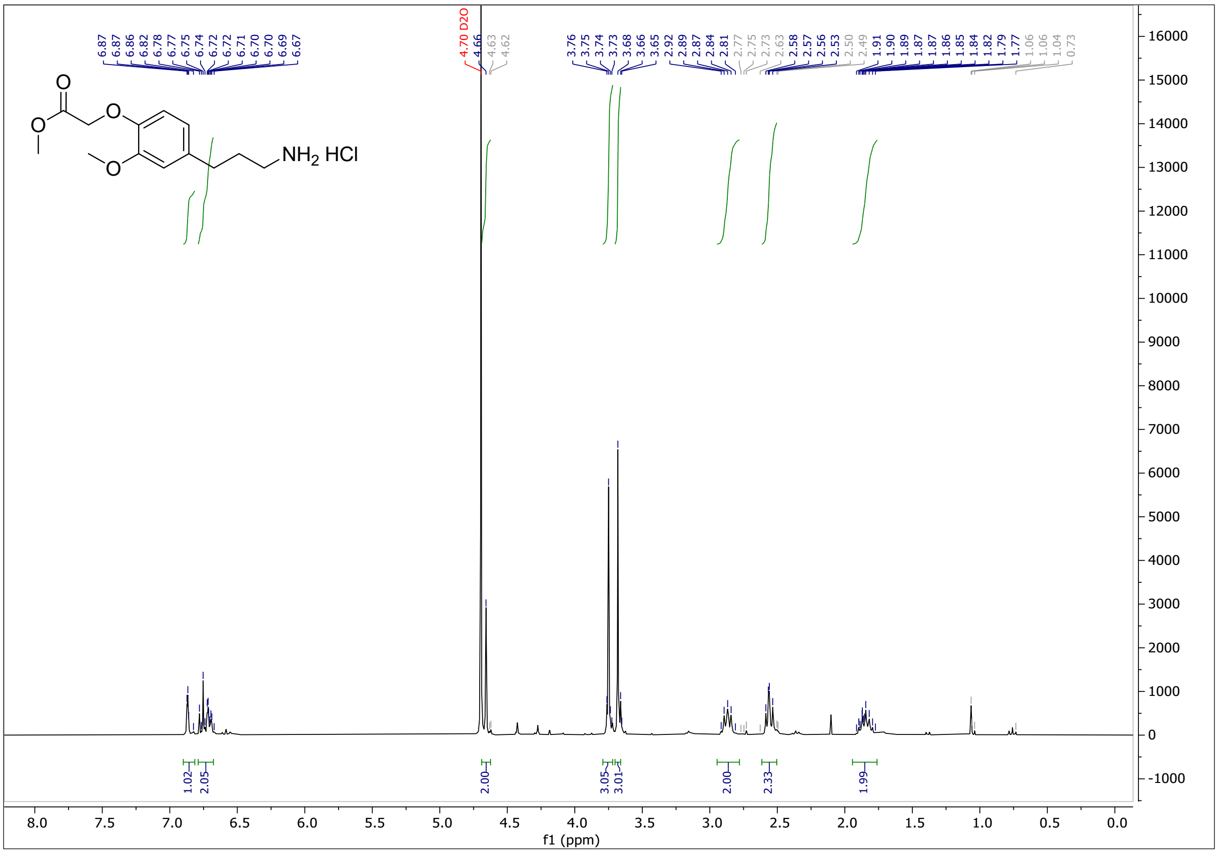

Figure S44. ^{1}H NMR spectrum of **4B** (as the HCl ammonium salt)

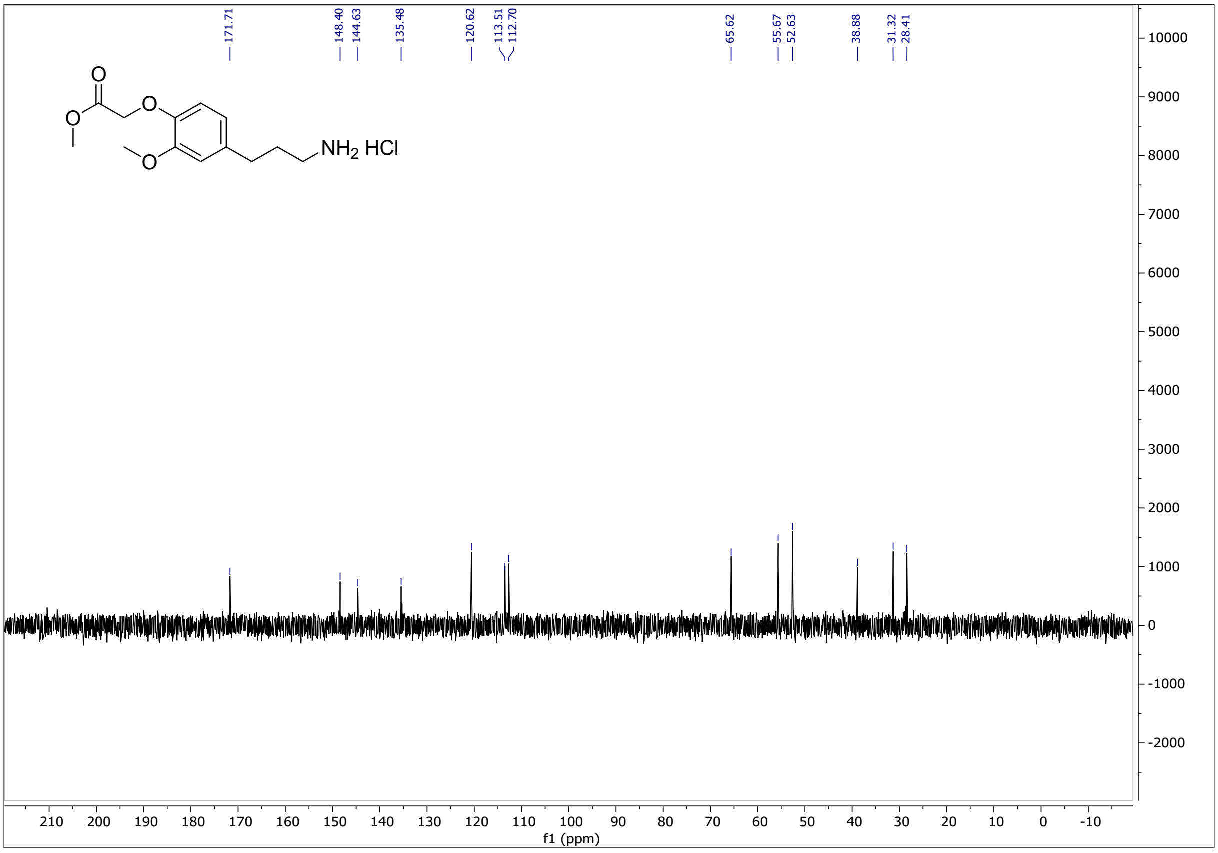

Figure S45. ^{13}C NMR spectrum of **4B** (as the HCl ammonium salt)

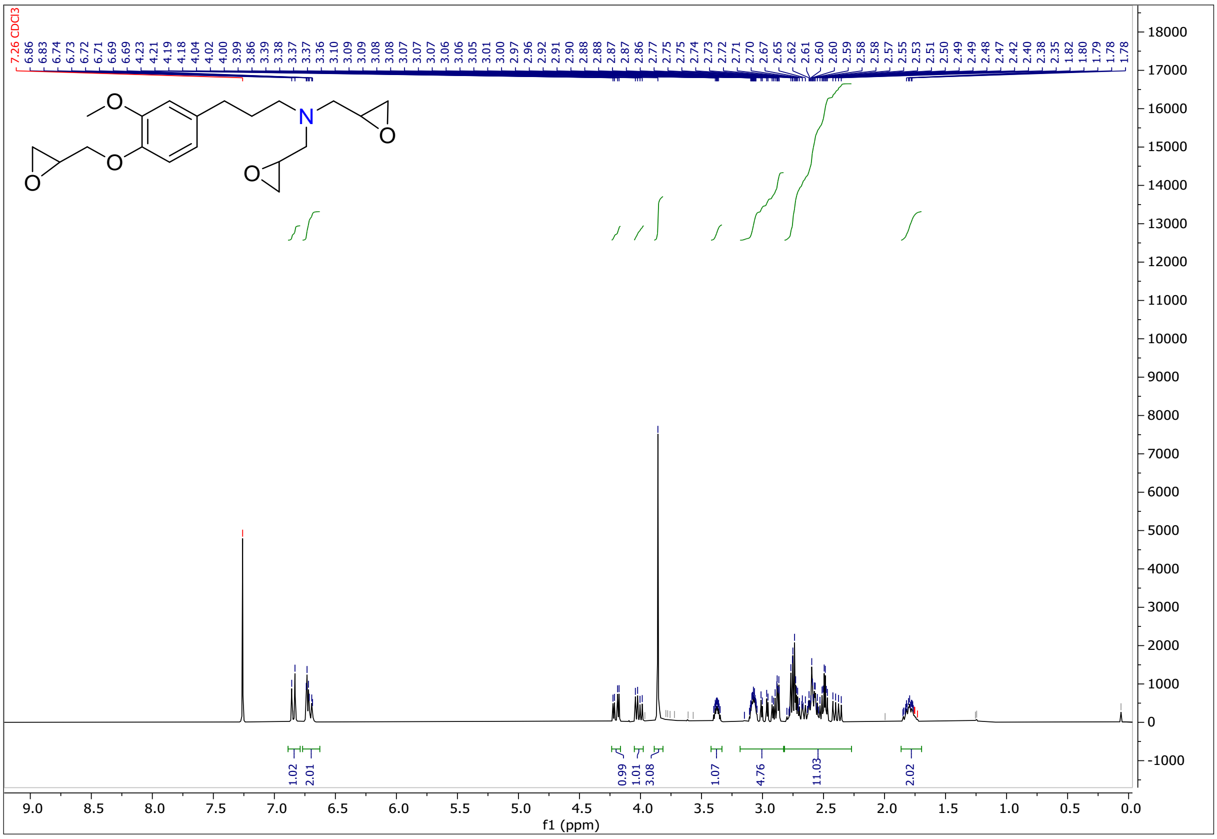

Figure S46. ^{1}H NMR spectrum of **5B**

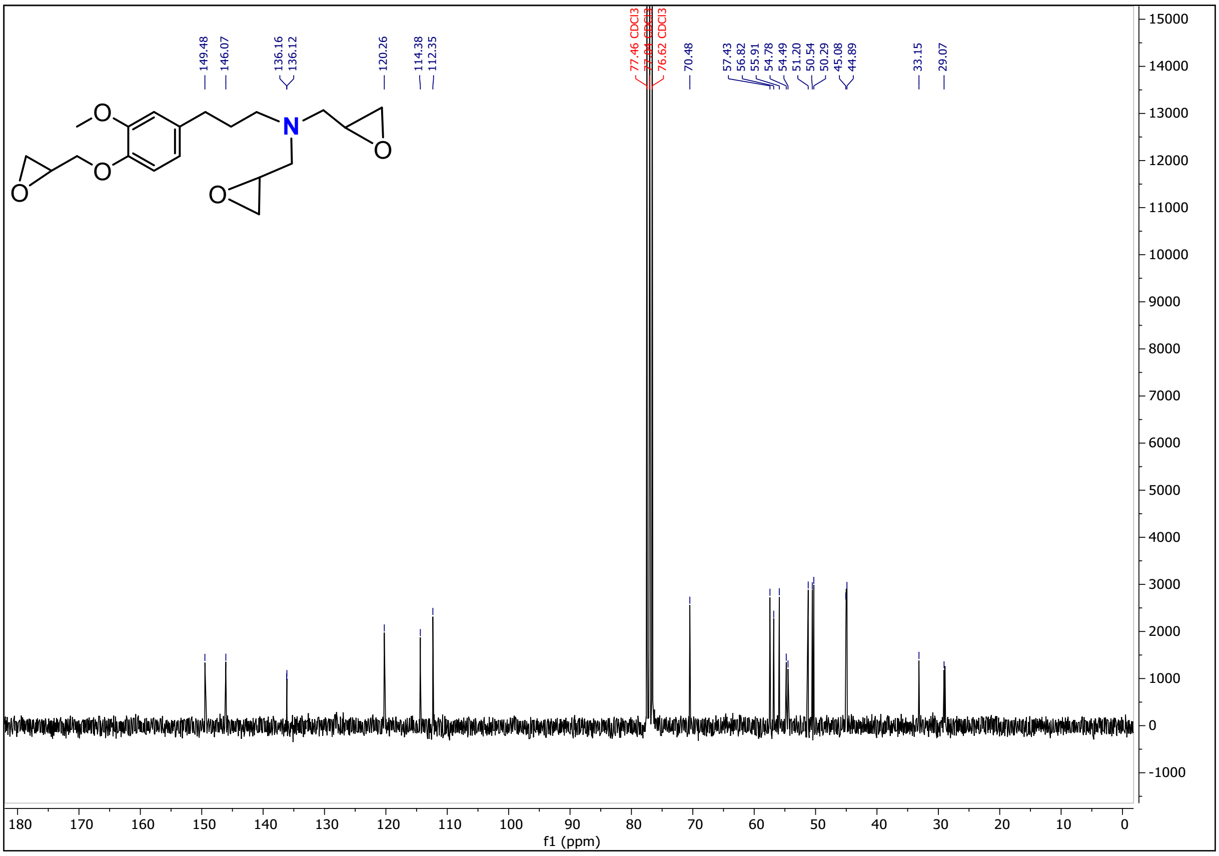

Figure S47. ^{13}C NMR spectrum of **5B**

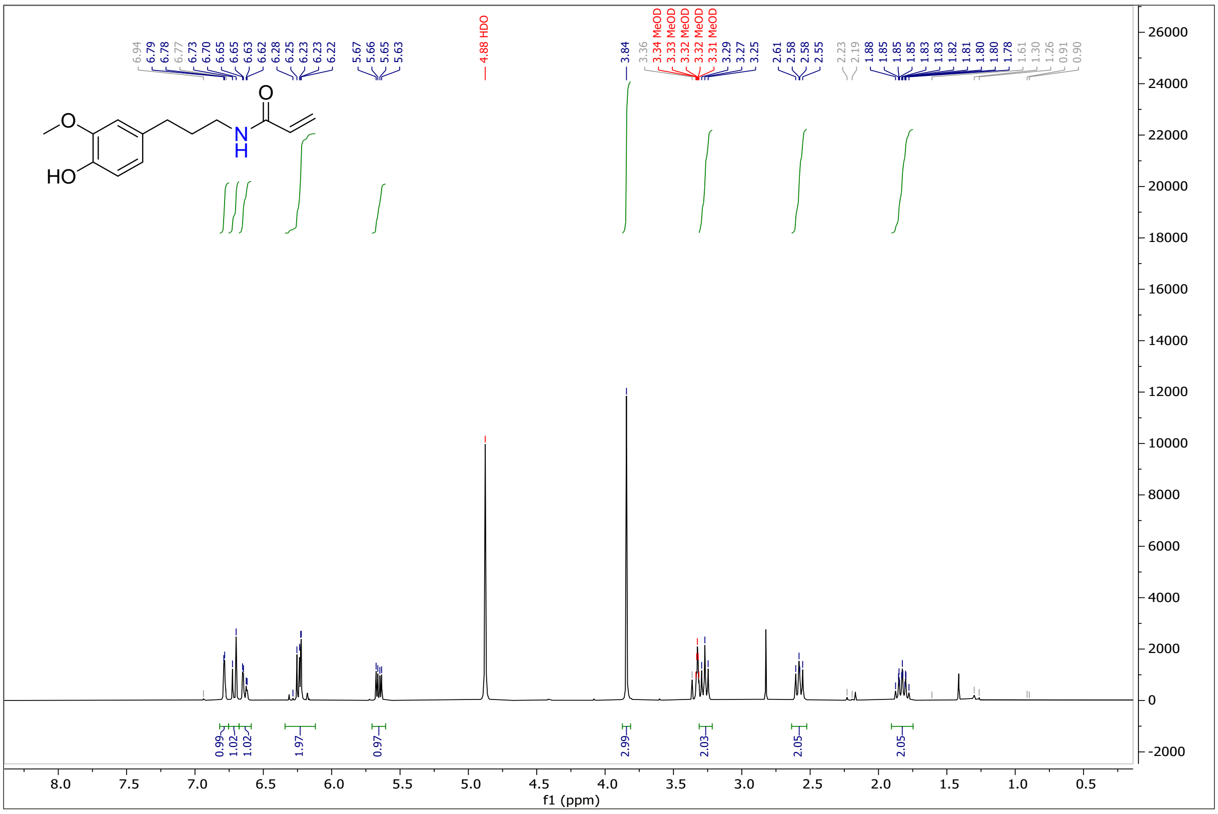

Figure S48. ^{1}H NMR spectrum of **6B**

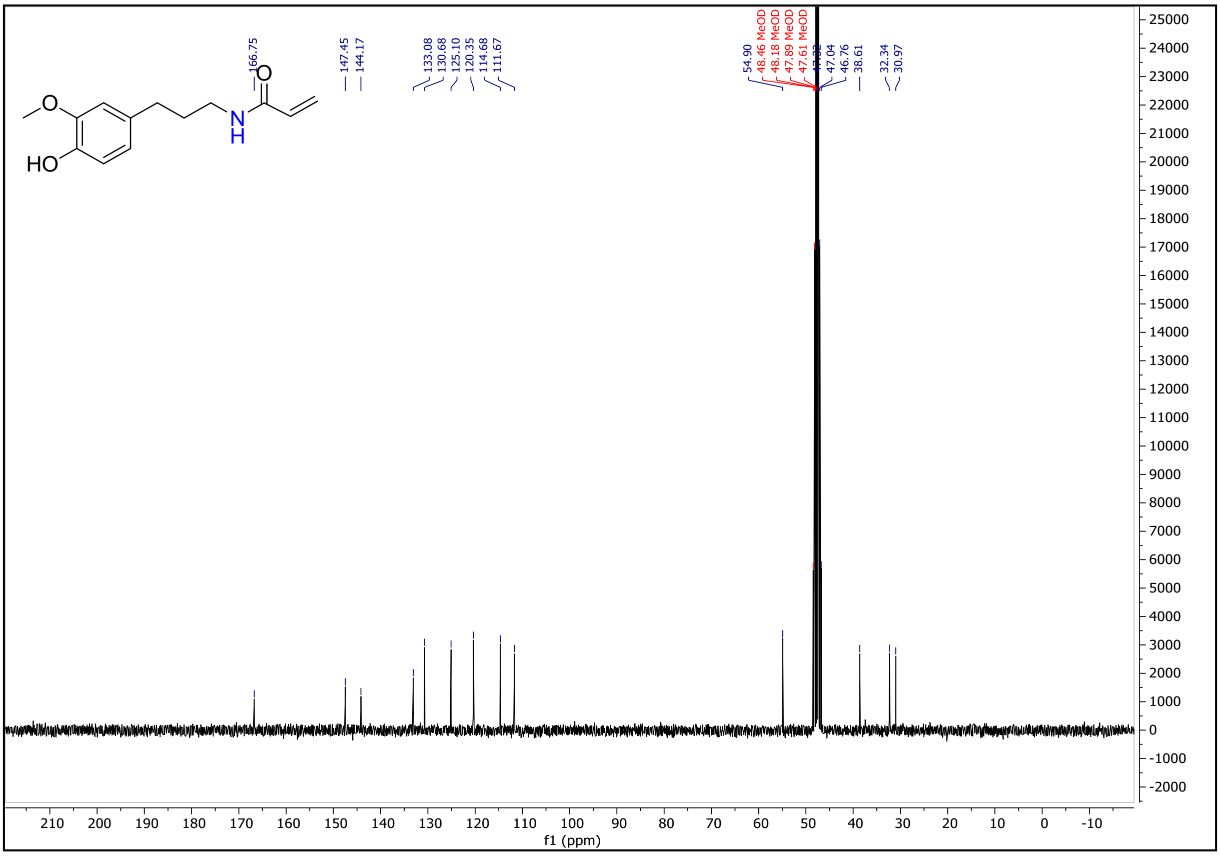

Figure S49. ^{13}C NMR spectrum of **6B**

5. List of the spectral and spectrometric data of the isolated compounds

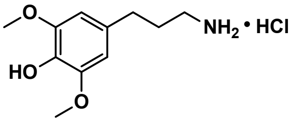

4-(3-Aminopropyl)-2,6-dimethoxyphenol hydrochloride (**1SA**), isolated yield: 64.5 %. ^{1}H NMR (300 MHz, D_2O) δ 6.52 (s, 2H), 3.73 (s, 6H), 2.93 – 2.82 (m, 2H), 2.54 (t, *J* = 7.6 Hz, 2H), 1.85 (h, *J* = 7.6, 7.1 Hz, 2H). ^{13}C NMR (75 MHz, D_2O) δ 147.68, 132.83, 132.08, 105.84, 56.23, 38.90, 31.79, 28.52. **HRMS** (APCI$^+$, m/z) calculated for $(M\text{-}H)^-$ 246.089759; found: 246.090245.

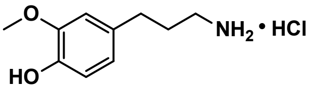

4-(3-Aminopropyl)-2-methoxyphenol hydrochloride (**1GA**), isolated yield: 75.8 %. ^{1}H NMR (300 MHz, D_2O) δ 6.82 (d, *J* = 2.0 Hz, 1H), 6.75 (d, *J* = 8.1 Hz, 1H), 6.65 (dd, J = 8.1, 2.0 Hz, 1H), 3.73 (s, 3H), 2.92 – 2.79 (m, 2H), 2.53 (t, J = 7.6 Hz, 2H), 1.83 (tt, J = 9.1, 6.8 Hz, 2H).^{13}C NMR (75 MHz, D_2O) δ 147.31, 142.98, 133.53, 120.96, 115.43, 112.71, 55.85, 38.91, 31.29, 28.55. **HRMS** (APCI$^+$, m/z) calculated for $(M\text{-}H)^-$ 216.079076; found: 216.07968.

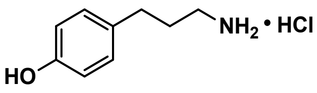

4-(3-Aminopropyl)phenol hydrochloride (**1HA**), isolated yield: 53.4 %. ^{1}H NMR (300 MHz, D_2O) δ 7.10 – 6.97 (d, 2H), 6.79 – 6.68 (d, 2H), 2.85 (t, *J* = 7.7 Hz, 2H), 2.51 (t, *J* = 7.7 Hz, 2H), 1.80 (tt, *J* = 9.2, 6.8 Hz, 2H).

^{13}C NMR (75 MHz, D_2O) δ 153.67, 132.74, 129.65, 115.37, 38.91, 30.83, 28.59. **HRMS** (APCI$^+$, m/z) calculated for $(M\text{-}H)^-$ 186.068999; found: 186.069115.

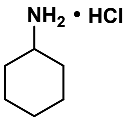

Cyclohexanamine hydrochloride (**1a**), isolated yield: 90.2 %. ^1H NMR (300 MHz, D_2O) δ 3.15 – 2.89 (m, 1H), 1.87 (m, J = 8.7, 4.7 Hz, 2H), 1.78 – 1.61 (m, 2H), 1.59 – 1.47 (m, 1H), 1.32 – 0.98 (m, 5H). ^{13}C NMR (75 MHz, D_2O) δ 50.32, 30.28, 24.24, 23.75. **HRMS** (APCI$^+$, m/z) calculated for $(M+Cl)^-$ 170.05049; found: 170.050878.

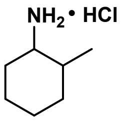

2-Methylcyclohexanamine hydrochloride (**2a**) (20cis/80trans), isolated yield: 68.2 %. ^1H NMR (300 MHz, D_2O) δ 3.27 (m, J = 6.1, 4.1 Hz, 0.2H), 2.70 (m, J = 11.0, 3.9 Hz, 0.8H), 2.01 – 1.78 (m, 1H), 1.73 – 0.79 (m, 11H). ^{13}C NMR (75 MHz, D_2O) δ 56.45, 52.85, 35.78, 33.14, 31.10, 30.58, 24.64, 24.18, 17.56. **HRMS** (APCI$^+$, m/z) calculated for $(M+Cl)^-$ 184.066248; found: 184.066529 (The rest of 5 cis-carbon signals were not detected because of cis in low quantities or overlapping with trans-carbon signals).

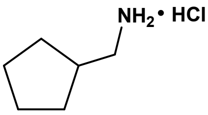

Cyclopentylmethanamine hydrochloride (**3a**), isolated yield: 75.2 %. ^1H NMR (300 MHz, D_2O) δ 2.83 (d, J = 7.6 Hz, 2H), 2.02 (m, J = 7.9 Hz, 1H), 1.80 – 1.65 (m, 2H), 1.59 – 1.41 (m, 4H), 1.24 – 0.94 (m, 2H). ^{13}C NMR (75 MHz, D_2O) δ 44.20, 37.43, 29.66, 24.56. **HRMS** (APCI$^+$, m/z) calculated for $(M+Cl)^-$ 170.05049; found: 170.050878.

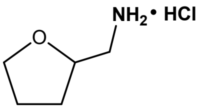

2-Tetrahydrofuranylmethanamine hydrochloride (**4a**), isolated yield: 45.1 %. ^1H NMR (300 MHz, D_2O) δ 4.09 (m, J = 10.0, 6.7, 3.4 Hz, 1H), 3.83 – 3.65 (m, 2H), 3.07 – 2.98 (m, 1H), 2.87 (dd, J = 13.2, 8.7 Hz, 1H), 2.09 – 1.93 (m, 1H), 1.93 – 1.74 (m, 2H), 1.60 – 1.47 (m, 1H). ^{13}C NMR (75 MHz, D_2O) δ 74.80, 68.24, 42.62, 28.20, 24.91. **HRMS** (APCI$^+$, m/z) calculated for $(M+Cl)^-$ 172.066667; found: 172.066529.

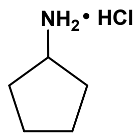

Cyclopentanamine hydrochloride (**5a**), isolated yield: 62.8 %. ^1H NMR (300 MHz, D_2O) δ 3.54 (m, J = 7.2, 5.4 Hz, 1H), 2.01 – 1.87 (m, 2H), 1.69 – 1.43 (m, 6H). ^{13}C NMR (75 MHz, D_2O) δ 52.07, 30.49, 23.48. **HRMS** (APCI$^+$, m/z) calculated for $(M+Cl)^-$ 156.035254; found: 156.035228.

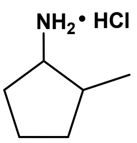

2-Methylcyclopentan-1-amine hydrochloride (**6a**) (22cis/78trans),Isolated yield: 58.6 %. ^1H NMR (300 MHz, D_2O) δ 3.47 (m, J = 6.8, 4.5 Hz, 0.2 H), 3.24 – 2.93 (m, 0.8 H), 2.05 – 0.85 (m, 10H). ^{13}C NMR (75 MHz, D_2O) δ 58.45, 55.27, 38.95, 35.72, 32.23, 30.76, 30.03, 29.50, 21.86, 21.32, 17.12, 12.96. **HRMS** (APCI$^+$, m/z) calculated for $(M+Cl)^-$ 170.050853 found: 170.050878.

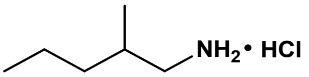

2-Methylpentan-1-amine hydrochloride (**7a**), isolated yield: 54.4 %. ^{1}H NMR (300 MHz, D_2O) δ 2.84 (m, J = 12.7, 6.0 Hz, 1H), 2.68 (m, J = 12.7, 7.8 Hz, 1H), 1.72 (dt, J = 11.8, 6.5 Hz, 1H), 1.33 – 1.04 (m, 4H), 0.80 (dd, J = 29.0, 6.8 Hz, 6H). ^{13}C NMR (75 MHz, D_2O) δ 45.03, 35.26, 30.55, 18.94, 16.08, 13.22. **HRMS** (APCI^{+}, m/z) calculated for $(M+Cl)^{-}$ 172.067; found: 172.066529.

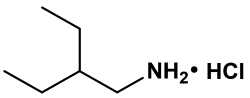

2-Ethylbutanamine hydrochloride (**8a**), isolated yield: 51.6 %. ^{1}H NMR (300 MHz, D_2O) δ 2.83 (d, J = 6.6 Hz, 2H), 1.48 (m, J = 6.5 Hz, 1H), 1.37 – 1.20 (m, 4H), 0.76 (t, J = 7.4 Hz, 6H). ^{13}C NMR (75 MHz, D_2O) δ 41.92, 38.39, 22.31, 9.52. **HRMS** (APCI^{+}, m/z) calculated for $(M+Cl)^{-}$ 172.066667; found: 172.066529.

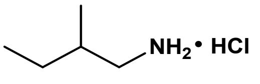

2-Methylbutanamine hydrochloride (**9a**), isolated yield: 43.5 %. ^{1}H NMR (300 MHz, D_2O) δ 2.85 (m, J = 12.7, 6.1 Hz, 1H), 2.68 (m, J = 12.7, 7.8 Hz, 1H), 1.71 – 1.53 (m, 1H), 1.31 (m, J = 13.0, 7.5, 5.5 Hz, 1H), 1.21 – 1.04 (m, 1H), 0.89 – 0.70 (m, 6H). ^{13}C NMR (75 MHz, D_2O) δ 44.68, 32.46, 25.92, 15.71, 10.04. **HRMS** (APCI^{+}, m/z) calculated for $(M+Cl)^{-}$ 158.050786; found: 158.050878.

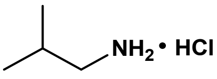

2-Methylpropanamine hydrochloride (**10a**), isolated yield: 43.8 %. ^{1}H NMR (300 MHz, D_2O) δ 2.72 (d, J = 7.2 Hz, 2H), 1.83 (m, J = 13.7, 6.8 Hz, 1H), 0.86 (d, J = 6.7 Hz, 6H). ^{13}C NMR (75 MHz, D_2O) δ 46.28, 26.22, 18.77. **HRMS** (APCI^{+}, m/z) calculated for $(M+Cl)^{-}$ 144.035296; found: 144.035228.

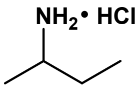

2-Butanamine hydrochloride (**11a**), isolated yield: 44.4 %. ^{1}H NMR (300 MHz, D_2O) δ 3.17 (m, J = 6.7 Hz, 1H), 1.66 – 1.37 (m, 2H), 1.16 (d, J = 6.7 Hz, 3H), 0.84 (t, J = 7.5 Hz, 3H). ^{13}C NMR (75 MHz, D_2O) δ 49.14, 27.10, 17.15, 8.91. **HRMS** (APCI^{+}, m/z) calculated for $(M+Cl)^{-}$ 144.03498; found: 144.035228.

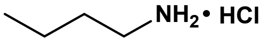

2-Butanamine hydrochloride (**12a**), isolated yield: 51.8 %. ^{1}H NMR (300 MHz, D_2O) δ 2.97 – 2.80 (t, 2H), 1.60 – 1.44 (m, 2H), 1.36 – 1.18 (m, 2H), 0.81 (t, J = 7.4 Hz, 3H). ^{13}C NMR (75 MHz, D_2O) δ 39.17, 28.67, 18.91, 12.66. **HRMS** (APCI^{+}, m/z) calculated for $(M+Cl)^{-}$ 144.035425; found: 144.035228.

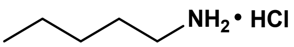

Pentanamine hydrochloride (**13a**), isolated yield: 64.2 %. ^{1}H NMR (300 MHz, D_2O) δ 2.87 (t, J = 7.6 Hz, 2H), 1.54 (m, J = 7.5 Hz, 2H), 1.23 (m, J = 7.7, 4.0 Hz, 4H), 0.86 – 0.69 (m, 3H). ^{13}C NMR (75 MHz, D_2O) δ 39.43, 27.65, 26.30, 21.39, 13.00. **HRMS** (APCI^{+}, m/z) calculated for $(M+Cl)^{-}$ 158.051083; found: 158.050878.

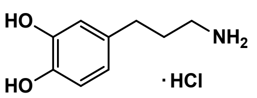

4-(3-aminopropyl)benzene-1,2-diol hydrochloride (**1B)**, isolated yield: 92 %. ^{1}H NMR (300 MHz, D2O) δ 6.74 (d, J = 8.1 Hz, 1H), 6.69 (d, J = 2.1 Hz, 1H), 6.59 (dd, J = 8.0, 2.2 Hz, 1H), 2.91 – 2.77 (m, 2H), 2.48 (t, J = 7.6 Hz, 2H), 1.80 (m, J = 9.2, 6.9 Hz, 2H). ^{13}C NMR (75 MHz, D_2O) δ 143.89, 142.09, 133.69, 120.57, 116.24, 116.11, 38.93, 30.99, 28.48. **HRMS** ($APCI^+$, m/z) calculated for $(M+H)^-$ 168.102073; found: 168.101905

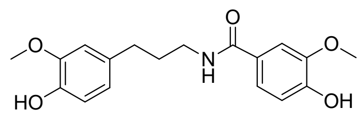

4-hydroxy-N-(3-(4-hydroxy-3-methoxyphenyl)propyl)-3-methoxybenzamide (**2B)**, isolated yield: 94.8 %. ^{1}H NMR (300 MHz, MeOH-d4) δ 7.42 (d, *J* = 2.1 Hz, 1H), 7.33 (dd, *J* = 8.3, 2.1 Hz, 1H), 6.88 – 6.79 (m, 2H), 6.74 – 6.63 (m, 2H), 3.91 (s, 3H), 3.83 (s, 3H), 3.39 (t, *J* = 7.1 Hz, 2H), 2.63 (t, *J* = 7.6 Hz, 2H), 1.98 – 1.86 (m, 2H). ^{13}C NMR (75 MHz, MeOH-d4) δ 168.66, 149.75, 147.46, 147.37, 144.18, 133.27, 133.58, 120.50, 120.37, 114.69, 114.40, 111.69, 110.46, 55.01, 54.90, 39.33, 32.58, 31.15. **HRMS** ($APCI^+$, m/z) calculated for $(M+H)^-$ 332.149507; found: 332.149249.

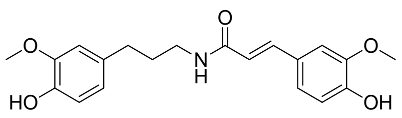

(E)-3-(4-hydroxy-3-methoxyphenyl)-N-(3-(4-hydroxy-3-methoxyphenyl)propyl)acrylamide (**3B)**, isolated yield: 83.0 %. ^{1}H NMR (300 MHz, MeOH-d4) δ 7.51 – 7.41 (d, 1H), 7.13 (d, J = 2.0 Hz, 1H), 7.03 (dd, J = 8.3, 2.0 Hz, 1H), 6.83 -6.64 (dd, 4H), 6.45 (d, J = 15.7 Hz, 1H), 3.86 (d, J = 15.1 Hz, 6H), 3.30 (d, J = 6.9 Hz, 2H), 2.59 (q, J = 7.1 Hz, 2H), 1.85 (m, J = 8.9, 6.8 Hz, 2H). ^{13}C NMR (75 MHz, MeOH-d4) δ 167.80, 148.41, 147.87, 147.46, 144.20, 140.56, 133.13, 126.87, 121.79, 120.39, 117.39, 115.06, 114.71, 111.70, 110.08, 54.96, 54.92, 46.77, 38.76, 32.41. **HRMS** ($APCI^+$, m/z) calculated for $(M+H)^-$ 358.165323; found: 358.164899

.

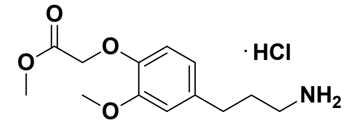

Methyl 2-(4-(3-aminopropyl)-2-methoxyphenoxy)acetate hydrochloride (**4B)**, isolated yield: 61.2 %.^{1}H NMR (300 MHz, D_2O) δ 6.87 (d, *J* = 1.9 Hz, 1H), 6.79 – 6.68 (m, 2H), 4.66 (s, 2H), 3.75 (s, 3H), 3.68 (s, 3H), 2.88 (q, *J* = 7.7, 7.1 Hz, 2H), 2.61 – 2.50 (m, 2H), 1.85 (m, *J* = 7.4 Hz, 2H). ^{13}C NMR (75 MHz, D_2O) δ 171.71, 148.40, 144.63, 135.48, 120.62, 113.51, 112.70, 65.62, 55.67, 52.63, 38.88, 31.32, 28.41. **HRMS** ($APCI^+$, m/z) calculated for $(M+H)^-$ 254.13895; found: 254.138685.

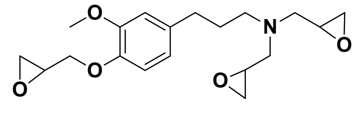

3-(3-methoxy-4-(oxiran-2-ylmethoxy)phenyl)-N,N-bis(oxiran-2-ylmethyl)propan-1-amine (**5B)**, isolated yield: 34.8 %. ^{1}H NMR (300 MHz, $CDCl_3$) δ 6.84 (d, *J* = 8.0 Hz, 1H), 6.77 – 6.63 (m, 2H), 4.20 (dd, *J* = 11.4, 3.6 Hz, 1H), 4.01 (dd, *J* = 11.4, 5.5 Hz, 1H), 3.86 (s, 3H), 3.37 (m, *J* = 5.5, 3.9, 2.6 Hz, 1H), 3.18 – 2.83 (m, 5H), 2.82 – 2.27 (m, 11H), 1.80 (m, *J* = 7.1, 3.2, 1.4 Hz, 2H). ^{13}C NMR (75 MHz, $CDCl_3$) δ 149.48, 146.07, 136.16, 136.12, 120.26, 114.38, 112.35, 77.24, 70.48, 57.43, 56.82, 55.91, 54.78, 54.49, 51.20, 50.54, 50.29, 45.08, 45.01, 44.89, 33.15, 33.11, 29.07, 28.91.

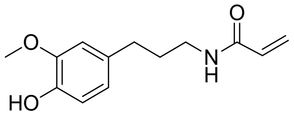

N-(3-(4-hydroxy-3-methoxyphenyl)propyl)acrylamide (**6B)**, isolated yield: 85.2 %. ^{1}H NMR (300 MHz, MeOH-d4) δ 6.79 (d, *J* = 1.9 Hz, 1H), 6.71 (d, *J* = 8.0 Hz, 1H), 6.64 (dd, *J* = 8.0, 2.0 Hz, 1H), 6.34 – 6.12 (m, 2H), 5.65 (dd, *J* = 8.3, 3.7 Hz, 1H), 3.84 (s, 3H), 3.27 (t, *J* = 7.1 Hz, 2H), 2.58 (dd, *J* = 8.5, 6.8 Hz, 2H), 1.83 (m, *J* = 7.6, 6.5 Hz, 2H). ^{13}C NMR (75 MHz, MeOH-d4) ^{13}C NMR (75 MHz, $CDCl_3$) δ 149.48, 146.07, 136.12, 120.26, 114.38, 112.35, 70.48, 57.43, 56.82, 55.91, 54.78, 54.49, 51.20, 50.54, 50.29, 45.08, 44.89, 33.15, 29.07.
